# Dynamic Modeling of CHO Cell Metabolism Using the Hybrid Cybernetic Approach With a Novel Elementary Mode Analysis Strategy

**DOI:** 10.3389/fbioe.2020.00279

**Published:** 2020-04-15

**Authors:** Juan A. Martínez, Dubhe B. Bulté, Martha A. Contreras, Laura A. Palomares, Octavio T. Ramírez

**Affiliations:** Departamento de Medicina Molecular y Bioprocesos, Instituto de Biotecnología, Universidad Nacional de México, Cuernavaca, Mexico

**Keywords:** elementary mode analysis, dynamic modeling, cybernetic modeling, CHO metabolism, metabolic engineering

## Abstract

Chinese hamster ovary (CHO) cell culture has a major importance on the production of biopharmaceuticals, including recombinant therapeutic proteins such as monoclonal antibodies (MAb). Mathematical modeling of biological systems can successfully assess metabolism complexity while providing logical and systematic methods for relevant genetic target and culture parameter identification toward cell growth and productivity improvements. Most modeling approaches on CHO cells have been performed under stationary constraints, and only a few dynamic models have been presented on simplified reaction sets, due to substantial overparameterization problems. The hybrid cybernetic modeling (HCM) approach has been recently used to describe the dynamic behavior by incorporating regulation between different metabolic states by elementary mode participation control, with sets of equations evaluated by objective functions. However, as metabolic networks evaluated are constructed toward a genomic scale, and cell compartmentalization is considered, identification of the active set becomes more difficult as EM number exponentially grows. Thus, the development of robust approaches for EM active set selection and analysis with smaller computational requirements is required to impulse the use of cybernetic modeling on larger up to genome-scale networks. In this report, a novel elementary mode selection strategy, based on a polar representation of the convex solution space is presented and coupled to a cybernetic approach to model the dynamic physiologic and metabolic behavior of CHO-S cell cultures. The proposed Polar Space Yield Analysis (PSYA) was compared to other reported elementary mode selection approaches derived from Common Metabolic Objective Analysis (CMOA) used in Flux Balance Analysis (FBA), Yield Space Analysis (YSA), and Lumped Yield Space Analysis (LYSA). For this purpose, exponential growth phase dynamic metabolic models were calculated using kinetic rate equations based on previously modeled growth parameters. Finally, complete culture dynamic metabolic flux models were constructed using the HCM approach with selected elementary mode sets. The yield space elementary mode- and the polar space elementary mode- hybrid cybernetic models presented the best fits and performances. Also, a flux reaction perturbation prediction approach based on the polar yield solution space resulted useful for metabolic network flux distribution capability analysis and identification of potential genetic modifications targets.

## 1. Introduction

Mammalian cell culture has significant importance on the production of biopharmaceuticals, including recombinant therapeutic proteins such as monoclonal antibodies (MAb) (Ahn and Antoniewicz, 2011; Meshram et al., 2011; Nolan and Lee, 2012; Rodrigues et al., 2012). Almost 70% of MAb for diagnostic and therapeutic applications are obtained from large scale mammalian cell cultures (Naderi et al., 2010). Furthermore, most of all recombinant therapeutic proteins are produced on mammalian cell cultures, and their global sales accounted for between $5 and 14 billion each by 2015 (Nolan and Lee, 2011; Jozala et al., 2016). Within mammalian cell lines, Chinese hamster ovary (CHO) cells have been the standard industrial host due to their well-known gene transfection, amplification, and clone selection technologies (Ahn and Antoniewicz, 2011; Nolan and Lee, 2011; Rodrigues et al., 2012). More so, CHO cell lines have shown to be stable, scalable, high-yield protein expression platforms with proper post-translational processing capabilities for several therapeutic applications (Nolan and Lee, 2011; Rodrigues et al., 2012; Kildegaard et al., 2013). However, productivity optimization is challenging, due to the intricate cellular machinery, compartmentalized metabolism and complex regulation and interconnection between multiple biological and media components that determine not only product quantity, but also quality (Palomares et al., 2004; Serrato et al., 2007; Hagrot et al., 2015). Systematic screening and derived statistical information have been useful, but often do not offer any insight on cell metabolism and regulation, making the exploration of multiple process and genetic modification targets difficult to assess and perform (Nolan and Lee, 2012; Hagrot et al., 2015).

Mathematical modeling of biological systems has been successfully used for assessing the metabolism complexity of many organisms while providing logical and systematic methods for growth and product enhancement (Haag et al., 2004; Naderi et al., 2010; Nolan and Lee, 2011, 2012; Ghorbaniaghdam et al., 2014). On the past two decades, many different models have been introduced for CHO cell lines to study growth (Naderi et al., 2010; Zamorano et al., 2010, 2013; Ahn and Antoniewicz, 2011; Meshram et al., 2011; Nicolae et al., 2015), media composition (Ahn and Antoniewicz, 2011; Nolan and Lee, 2011; Hagrot et al., 2015; Nicolae et al., 2015), culture parameters (Nolan and Lee, 2011; Martínez et al., 2015), determination of key metabolites for by-product accumulations (Nolan and Lee, 2012; Martínez et al., 2015), and amino acid metabolism (Zamorano et al., 2010, 2013; Ahn and Antoniewicz, 2011; Hagrot et al., 2015; Rejc et al., 2017). Within them, the most used modeling approaches, are metabolic flux analysis (MFA) and Flux Balance Analysis (FBA), which are based on pseudo-steady state mass flux balances (Nolan and Lee, 2011). These approaches can provide a reliable snapshot in time for the distribution of internal fluxes on specific conditions (Nolan and Lee, 2011; Ghorbaniaghdam et al., 2014). Nevertheless, it is seldom when steady states are prevalent on biological processes, especially in mammalian cell cultures, where a constant dynamic response to medium changes, concentrations between compartments, and other perturbations are observed and sometimes relied on for biotechnological production purposes (Nolan and Lee, 2012). This characteristic stresses the need to design and construct dynamic approaches to better assess changes in metabolism (Nolan and Lee, 2012; Ghorbaniaghdam et al., 2014).

Up to this day, few dynamic metabolic models have been published for CHO cells with approaches such as mechanistic and hybrid models. Mechanistic models define algebraic expressions for the reaction rates as a function of metabolite concentrations but are often difficult to perform, as they generally rely on the estimation of a large number of kinetic parameters, and therefore usually limited to a small number of reactions (Nolan and Lee, 2012; Hagrot et al., 2015). On the other hand, hybrid models establish different culture stages and calculate the average flux on each step, along with MFA. These models are used to address changes in the metabolic conditions along a culture but often present difficulties for revealing the temporal evolution of individual fluxes providing little information about their regulation (Martínez et al., 2015). Regarding this last-mentioned limitation, the dynamic cybernetic modeling approach has been developed, by Ramkrishna and collaborators, to address dynamic changes even with suboptimal information of the mechanistic regulatory details (Kompala et al., 1986; Varner and Ramkrishna, 1999; Ramkrishna and Song, 2012; Song and Ramkrishna, 2012).

Cybernetic modeling (CM) introduces regulation using two dynamic vector variables, which modify the participation of minimal and unique reaction flux subsets, or elementary modes (EM), obtained from the stoichiometric matrix analysis. These cybernetic variables are regulated by objective functions evaluated with sets of matching law equations. This approach has been proved useful to describe dynamic metabolic state fluxes and regulation even with little information on the mechanistic particularities, allowing reasonable dynamic flux distribution modeling (Song and Ramkrishna, 2010; Ramkrishna and Song, 2012). The hybrid cybernetic approach relies on the selection of a small and feasible subset of active EM that can describe the experimental observations. Therefore, a most critical limitation arises as metabolic networks evaluated are constructed toward a genome-scale, making identification of the active set more difficult and computationally intensive as EM number exponentially grows (Song and Ramkrishna, 2010). On that regard, Song and Ramkrishna have developed EM analysis and selection strategies based on transforming the EM information from the flux solution space to a yield solution space and performing linear programming and integer programming solution calculations to select a subset of active EM or a lumped vector with all the known experimental yield data (Song and Ramkrishna, 2009, 2010, 2012; Ramkrishna and Song, 2012). With these approaches, they have been able to successfully model the metabolism of multiple organisms with more extensive networks but still far from genome-scale. Thus, the development of new robust approaches for EM active set selection and analysis with smaller computational requirements could impulse the possibility of using of cybernetic modeling toward genome-scale networks. The work presented herein pretends to contribute to reduce computational requirements of EM selection analyses and to extend on predictive capabilities with EM Analysis (EMA) for dynamic cybernetic modeling.

In this report, a novel EMA and selection approach is proposed, evaluated, and coupled with hybrid cybernetic models to describe a CHO-S cell line metabolic and phenotype behavior during a batch process fermentation. The presented approach relies on a variable description transformation from the Cartesian convex solution space (flux or yield) to an angular space or polar description, to determine a reduced and feasible EM active set. This novel PSYA was compared to other EMA approaches derived from optimization around CMOA often used in FBA (Schuetz et al., 2007), Yield Space Analysis (YSA), and Lumped Yield Space Analysis (LYSA) (Song and Ramkrishna, 2009, 2012; Ramkrishna and Song, 2012). Kinetic dynamic models were initially calculated for comparison during the exponential growth phase using rate equations derived from descriptive physiological models. Whole bioprocess dynamic models were then built using selected EM and the hybrid cybernetic modeling (HCM) approach. The PSYA- and YSA- HCM approaches were found to have the best performance regarding experimental data fitting and CHO-S cell metabolic characteristics. Also, a reaction flux perturbation prediction approach based on the PSYA is proposed. Such EM Perturbation Analysis (EMPA) was useful for modified reaction flux distribution analysis and the identification of potential genetic targets.

## 2. Materials and Methods

### 2.1. Strains and Cultures

An Invitrogen aneuploid CHO-S® cell line was used to obtain the experimental data for modeling. Cultures were performed by triplicate on 25 mL shake-flasks incubated at 130 rpm at 37°C. Cultures were inoculated to start at 1 × 10^6^ cells/mL on CD FortiCHO (Gibco) media containing 28 mM glucose (GLC) and supplemented with 8 mM glutamine (GLN). Cultures were kept for 144 h and samples were taken every 24 h. Samples were analyzed for viable (*X*_*v*_) and dead (*X*_*d*_) cell concentrations by cell counting with the trypan blue exclusion. Major dissolved components, GLC, lactic acid (LAC), GLN, and glutamic acid (GLU), were determined on culture supernatant, with a YSI 2950D biochemical analyzer equipped with specific membranes for each compound. Specific growth, uptake and production rates approximations were obtained by linear regressions during mid-exponential growth phase and mid stationary/cell-death phase and used as initial values for model parameter determination. Biomass units were transformed from cells/mL to mM with a conversion established by Nolan and Lee (2011).

### 2.2. Physiological Model Construction and Analysis

Models were constructed with differential mass balance equation sets for culture macroscopic characterization. Total biomass (*X*) was set to be the sum of viable (*X*_*v*_) and dead (*X*_*d*_) biomass and described by a classical logistic growth equation (Equation 1). *X*_*d*_ was described by Equation (2). Finally, *X*_*v*_ was obtained by the difference between both previously described equations, such that:

(1) $$\begin{array}{l} \frac{dX}{dt}=\frac{dX_{v}}{dt}+\frac{dX_{d}}{dt}=\mu_{max}X\left(1-\frac{X}{X_{max}}\right) \end{array}$$

(2) $$\begin{array}{l} \frac{dX_{d}}{dt}=k_{d}X_{d} \end{array}$$

(3) $$\begin{array}{l} \Rightarrow \frac{dX_{v}}{dt}=\frac{dX}{dt}-\frac{dX_{d}}{dt}=\mu_{max}X\left(1-\frac{X}{X_{max}}\right)-k_{d}X_{d} \end{array}$$

where μ_*max*_ is the maximum biomass growth rate, *X*_*max*_ is the maximum total biomass and *k*_*d*_ is the maximum biomass death rate. For the major dissolved components on media (substrates or products) models were constructed starting from the following simple differential equation for any external metabolite (*M*_*i*_) concentration:

(4) $$\begin{array}{l} \frac{dM_{i}}{dt}=q_{i}X_{v} \end{array}$$

where *q*_*i*_ refers to its specific rate of consumption/production. To better describe the behavior during cell growth, viable cells can be segregated into two states: a growth metabolic state and a stationary metabolic state (before cell death). Therefore, the two populations with different metabolic characteristics can be described as:

(5) $$\begin{array}{l} X_{v}=X_{v}^{e}+X_{v}^{s} \end{array}$$

where $X_{v}^{e}$ is related to the viable growing cells and $X_{v}^{s}$, to the viable cells in a stationary state. These different populations can then be expressed with respect to the total *X*_*v*_ as:

(6) $$\begin{array}{l} X_{v}^{e}=X_{v}\Psi^{e};dX_{v}^{s}=X_{v}\Psi^{s} \end{array}$$

where Ψ^*e*^ and Ψ^*s*^ represent fractional allocations of cells on the different stages, such that Ψ^*e*^ + Ψ^*s*^ = 1 (Martinez et al., 2018). This fractional allocation can be dynamically represented using the changing ratio between *X* and *X*_*max*_ during the different culture phases:

(7) $$\begin{array}{l} \Psi^{e}=\left(1-\frac{X}{X_{max}}\right) \end{array}$$

(8) $$\begin{array}{l} \Psi^{s}=\left(\frac{X}{X_{max}}\right) \end{array}$$

Substitution of Equations (6) to (8) into Equation (4) allows the expression the external metabolite differential equations as:

(9) $$\begin{array}{l} \frac{dM_{i}}{dt}=q_{i}^{e}X_{v}\Psi^{e}+q_{i}^{s}X_{v}\Psi^{s}=q_{i}^{e}X_{v}\left(1-\frac{X}{X_{max}}\right)+q_{i}^{s}X_{v}\left(\frac{X}{X_{max}}\right) \end{array}$$

Finally, the specific rates *q*_*i*_ for each phase can be defined as Michaelis–Menten equations sets with maximum specific rates and saturation constants for each *M*_*i*_ regarding the key metabolite(s) consumed for its production/consumption. As this equation is difficult to resolve analytically, a numeric approximation for the differential can then be proposed. Therefore, the extended model equations for the CHO-S external metabolites production/consumption constructed for the metabolites measured on this report were:

(10) $$\begin{array}{l} GLC_{\left(t\right)}=GLC_{\left(t-\Delta t\right)}+q_{g}^{e}\left(\frac{{\left[GLC\right]}_{\left(t-\Delta t\right)}}{K_{g}+{\left[GLC\right]}_{\left(t-\Delta t\right)}}\right)X_{v\left(t\right)}\Psi_{t}^{e}\Delta t\\ \;+q_{g}^{s}\left(\frac{{\left[GLC\right]}_{\left(t-\Delta t\right)}}{K_{g}+{\left[GLC\right]}_{\left(t-\Delta t\right)}}\right)X_{v\left(t\right)}\Psi_{t}^{s}\Delta t \end{array}$$

(11) $$\begin{array}{l} GLN_{\left(t\right)}=GLN_{\left(t-\Delta t\right)}+q_{n}^{e}\left(\frac{{\left[GLN\right]}_{\left(t-\Delta t\right)}}{K_{n}+{\left[GLN\right]}_{\left(t-\Delta t\right)}}\right)X_{v\left(t\right)}\Psi_{t}^{e}\Delta t\\ \;+q_{n}^{s}\left(\frac{{\left[GLN\right]}_{\left(t-\Delta t\right)}}{K_{g}+{\left[GLN\right]}_{\left(t-\Delta t\right)}}\right)X_{v\left(t\right)}\Psi_{t}^{s}\Delta t \end{array}$$

(12) $$\begin{array}{l} LAC_{\left(t\right)}=LAC_{\left(t-\Delta t\right)}+q_{l}^{e}\left(\frac{{\left[GLC\right]}_{\left(t-\Delta t\right)}}{K_{g}+{\left[GLC\right]}_{\left(t-\Delta t\right)}}\right)X_{v\left(t\right)}\Psi_{t}^{e}\Delta t\\ \;+q_{l}^{s}\left(\frac{{\left[LAC\right]}_{\left(t-\Delta t\right)}}{K_{l}+{\left[LAC\right]}_{\left(t-\Delta t\right)}}\right)X_{v\left(t\right)}\Psi_{t}^{s}\Delta t \end{array}$$

(13) $$\begin{array}{l} GLU_{\left(t\right)}=GLU_{\left(t-\Delta t\right)}+q_{u}^{e}\left(\frac{{\left[GLN\right]}_{\left(t-\Delta t\right)}}{K_{n}+{\left[GLN\right]}_{\left(t-\Delta t\right)}}\right)X_{v\left(t\right)}\Psi_{t}^{e}\Delta t\\ \;+q_{u}^{s}\left(\frac{{\left[GLN\right]}_{\left(t-\Delta t\right)}}{K_{n}+{\left[GLN\right]}_{\left(t-\Delta t\right)}}\right)X_{v\left(t\right)}\Psi_{t}^{s}\Delta t \end{array}$$

where sub-indexes *g*, *n*, *l*, *u*, and *h* refer to GLC, GLN, LAC, and GLU, respectively. The *q* parameters refer to the maximum specific production/consumption rates and are specific for exponential (*e*) or stationary (*s*) phases. The *K* parameters refer to the Michaelis–Menten saturation constants for each key metabolite. Note that the Michaelis–Menten sections of the presented model can be extended for many key metabolites, but on this report only the main contributions (GLC, GLN, and LAC) were selected in order to reduce the number of parameters. Equations (10) to (13) were numerically integrated with Δ*t* = 0.1*h*, which is at least three orders of magnitude below from those where sensible changes on growth and metabolite concentration occur. Production and consumption rates were approximated by Sum of the Square Error (SSE) against experimental data. Models constructed were evaluated by the Mean Absolute Percentage Error (MAPE), the Prediction Error (PE), and a Mean Prediction Percentage Error (MPPE) calculations (Zhao and Kurata, 2010; Kim and Kim, 2016; Martinez et al., 2018), as follows:

(14) $$\begin{array}{l} SSE=\overset{n}{\underset{1}{\sum }}{\left(e-m\right)}^{2} \end{array}$$

(15) $$\begin{array}{l} PE={\left(\frac{SSE}{n}\right)}^{1/2} \end{array}$$

(16) $$\begin{array}{l} MAPE=\frac{100}{n}\overset{n}{\underset{1}{\sum }}|\frac{e-m}{e}| \end{array}$$

(17) $$\begin{array}{l} MPPE={\left(\frac{SSE}{\left(\sum_{1}^{n}e^{2}\right)n}\right)}^{1/2} \end{array}$$

where *e* and *m* refer to the experimental and modeled data points respectively, and *n* is the number of data points.

### 2.3. Metabolic Models Construction and Analysis

#### 2.3.1. Metabolic Network Construction

**Figure 1:**
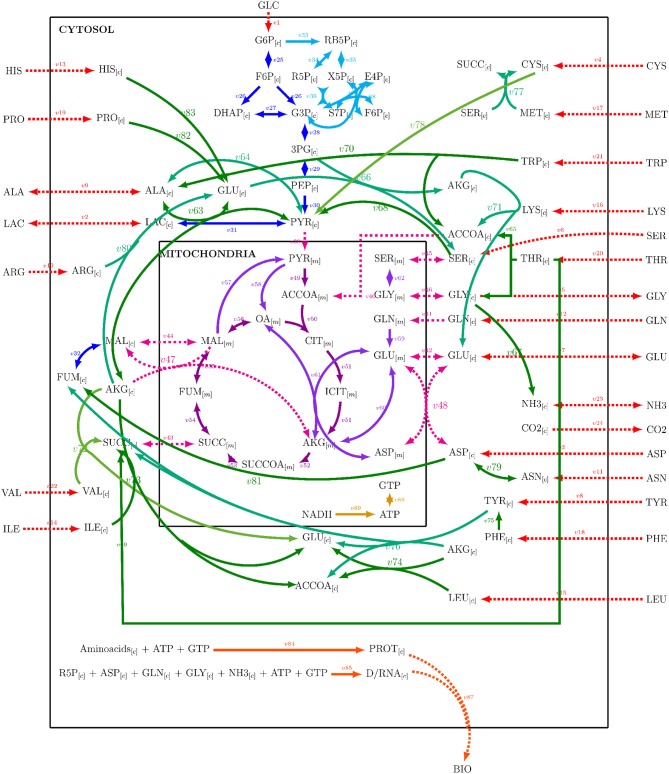
Reaction network used for stoichiometric matrix construction for metabolic modeling.

A metabolic network was constructed from Nolan and Lee (2011), Ahn and Antoniewicz (2011), Zamorano et al. (2010), Nicolae et al. (2015), and Robitaille et al. (2015) networks. The metabolic network was integrated by adding the reactions listed by the cited authors, repeated entries were eliminated, and some reactions were combined. The resulting network comprised 89 reactions with 25 extracellular metabolites and 62 intracellular metabolites and is presented on Supplementary Material 1. Mitochondrial compartmentalization was performed by separating metabolite pools dependent on mitochondrial transport reactions. The metabolic network considered glycolysis, pentose phosphate pathway, tricarboxylic acid cycle, amino acid metabolism, DNA, RNA and protein synthesis, mitochondrial transport, biomass formation, and energetic metabolism reactions. Figure 1 presents a simplified representation of the reaction network used on this work, reaction, and metabolites names will be used as shown forward on, and their complete description can be found on Supplementary Material 1. A stoichiometric matrix was constructed and then expanded to a series of EM computed with the efmtool protocol tool (Terzer and Stelling, 2008) embedded in MATLAB.

#### 2.3.2. Elementary Mode Analysis and Selection

For the dynamic modeling of cell line metabolism, a novel approach for EMA and selection was constructed in this work. This approach consisted on reconstructing the convex solution space from a Cartesian description, where fluxes or yields are described by vertexes positions, to a Polar representation, where angles and modules describe their magnitude and proportion. This reconstruction allows to redistribute the convex solution space and characterize its structure from another perspective, where subsets of consecutive angles and modules represent a pathway across the polytope, compressing network information. For a better description of the approach, it can be stated that every EM on the convex hull can be described by its position on the convex space as a vector consisting on the yields for each coordinate as:

(18) $$\begin{array}{l} EM_{i}=\left[Y_{1}^{i},Y_{2}^{i},Y_{3}^{i},\ldots ,Y_{n-1}^{i},Y_{n}^{i}\right] \end{array}$$

where *Y*_*n*_ represents the yield of the *n*^*th*^ reaction of the network within the *i*^*th*^ EM. However, yield convex solution space hull information can also be described as a module for each EM (λ_*i*_) and angles θ_*i*_ describing its directions across the polytope, as follows:

(19) $$\begin{array}{l} \lambda_{i}={\left(Y_{1}^{2}+Y_{2}^{2}+Y_{3}^{2}+\ldots +Y_{n-1}^{2}+Y_{n}^{2}\right)}^{1/2} \end{array}$$

(20) $$\begin{array}{l} \theta_{i}=cos^{-1}\left[\frac{{\left(\overset{j}{\underset{1}{\sum }}Y_{j}^{2}\right)}^{1/2}}{{\left(\overset{j-1}{\underset{1}{\sum }}Y_{k}^{2}\right)}^{1/2}}\right];2<j\leq n \end{array}$$

where *j* is the number of reactions yields that are being addressed for a θ angle, up to the total *n*^*th*^ reaction. Therefore, the convex solution hull can be transformed from a Cartesian convex hull space to multiple Polar convex spaces depending on different θ_*i*_ subsets, derived from different yield compositions and orders used for calculations. Each subset of angles does not only reflect the information about yields, but their ordered relationship and ratio. As a result, a set of calculated angles can be thought of as a pathway description through the n-dimensional polytope of the solution space.

The modules and angles from any set can then be conformed into two vectors that define the polar convex hull description Λ and Θ. From which also a parallel subset can be calculated containing only the known reaction yields, such that:

(21) $$\begin{array}{l} \Lambda =\left[\lambda_{1},\lambda_{2}\ldots ,\lambda_{n}\right]\|\Lambda^{\epsilon }=\left[\lambda_{1}^{\epsilon },\lambda_{2}^{\epsilon }\ldots ,\lambda_{n}^{\epsilon }\right] \end{array}$$

(22) $$\begin{array}{l} \Theta =\left[\theta_{1},\theta_{2},\ldots ,\theta_{n-1}\right]\|\Theta^{\epsilon }=\left[\theta_{1}^{\epsilon },\theta_{2}^{\epsilon },\ldots ,\theta_{n-1}^{\epsilon }\right] \end{array}$$

where super-index ϵ refers to the vectors calculated using only the EM values for reactions with known experimental yield data. Experimental yields can also be located on the solution space with similar vectors, Φ for the angle information and Γ as the scalar module. This allows an easy comparison of all EM, where at least one can be selected by finding the minimum difference with the experimental data, given by:

(23) $$\begin{array}{l} \Upsilon^{\Lambda }={\left[\frac{\Lambda^{\epsilon }-\Gamma }{\Gamma }\right]}^{2} \end{array}$$

(24) $$\begin{array}{l} \Upsilon_{\left(1..n\right)}^{\Theta }={\left[(\Theta^{\epsilon }-\Phi )\cdot \frac{1}{\Phi }\right]}^{2} \end{array}$$

(25) $$\begin{array}{l} min\left[\Upsilon \right]=min\left[\Upsilon^{\Lambda }+\Upsilon^{\Theta }\right] \end{array}$$

If Υ for the selected EM equals 0, then a single *EM*^ι^ can be used to describe the metabolic behavior. However, this happens seldom, so a minimal active set should be constructed around the experimental data. The next EM are selected consecutively by maximizing the distance to the middle point between the previously selected EM while minimizing the distance to the experimental data point. This procedure allows the construction of a polyhedral structure composed of the minimal set of *EM*^*act*^ containing the polar space experimental data point. Also, this *EM*^*act*^ can be lumped by a linear combination of the selected EM. The algorithmic implementation diagram of the presented approach can be found in Supplementary Material 1.

It's essential to notice that multiple Θ, Θ^ϵ^, and Φ sets can be calculated as stated before, as different starting angle calculation orders can be performed, each one describing a particular path inside the polytope toward the point it is describing, and therefore, representing different information about yield ratios between different reactions. This arises a particularly interesting property of the solution space: if all permutations constructing all the Λ^ϵ^ sets for a particular EM or destination Φ, given they are parallel (meaning being calculated in the same yield order), gives the same results for the closest selected EM, then the solution space may be orthogonal for all its coordinates. However, if the results are different, the solution space must be a non-Euclidean space. In fact, for the data presented on this work, various permutations were performed, and slightly different solutions were found (different closest *EM*_ι_ to Φ, but within the same fist ≈15 closest EM in different orders). However, extended experiments and calculations to this possible conjecture will be presented elsewhere, as it is not in the scope of this report. In this work, yields used for calculation vector definitions were *Y*_*g*/*x*_, *Y*_*n*/*x*_, *Y*_*l*/*x*_, and *Y*_*u*/*x*_, and used in that order as chosen by the following ordering rules: (1) substrates before products, (2) higher consumption/production participation. This definition allowed to select a minimal set of EM with PSYA and Lumped Elementary Mode Analysis (LPSYA) to describe the metabolic behavior.

#### 2.3.3. Elementary Mode Analysis Validation and Comparison

To analyze and compare PSYA and LPSYA approaches, different EM selection approaches were also used for the construction of dynamic exponential growth metabolic models. An EMA selection approach was derived from the minimization analysis of metabolic objectives (CMOA) commonly used in FBA (Schuetz et al., 2007). The used metabolic objectives were max biomass yield over GLC, max biomass yield over GLN, max ATP yield over GLC, max ATP yield over GLN, max ATP yield over flux unit, max biomass over flux unit, max GLC consumption over biomass, max GLN consumption over biomass, max ATP yield over reaction step, minimum reaction steps, and minimum flux units. Also, YSA and LYSA, as developed by Song and Ramkrishna (2009), were used for EMA comparison. All different EMA methods were used on EM sets calculated from the metabolic network reduced by 4, 6, and 8 constraints. These constraints were constructed either from experimental observations or known CHO metabolism and were: (1) v87 > 0 (biomass production), (2) v12 > 0 (GLN consumption), (3) v2r > 0 (LAC production), (4) v7r > 0 (GLU production), (5) 39 > 0 (Pyruvate mitochondrial transport), (6) v53r > 0 (TCA cycle), (7) v41 > 0 (GLN mitochondrial transport), (8) v30 > 0 (PEP to PYR cytosol conversion). They are expressed as on the stoichiometric matrix (Supplementary Material 1), in the most biological sense or prevailing direction (e.g., GLC ext → GLC cytosol), where the sign referrers to the reaction direction (forward or reverse). The three subsets presented sequentially smaller sizes and were used to analyze the EMA selection methods for computational time requirements and dynamic modeling accuracy.

The selected EM from all EMA approaches were used then to render metabolite consumption/productions profiles with the use of the physiological model of biomass growth described previously, where Δ[*X*] were calculated for each Δ*t* of 0.1 h, as follows:

(26) $$\begin{array}{l} \Delta M_{i}=\left[\frac{Z_{i}}{Z_{X}}\right]\Delta X \end{array}$$

where *Z*_*i*_ refers to the stoichiometric flux index for the *i*^*th*^ metabolite, and *Z*_*X*_ refers to the stoichiometric flux index for the biomass on the EM flux vector. This equation allowed to obtain dynamic profiles for the metabolic outputs and internal fluxes within the first 48 h of fermentation (the first section of the exponential growth phase). The dynamic exponential metabolic models constructed were evaluated for their experimental data approximation performance by SSE, PE, MAPE, and MPPE and their CPU usage times. All calculations were performed on MATLAB with a Lenovo, Intel Core i7, 2.5 GHz computer with 16.0 GB RAM, and Windows 10 running only the operative system and MATLAB. CPU usage times were calculated considering only the EMA sections without network loading, any plotting, displaying or other processing, and normalized against EM mode number for each constrained system.

#### 2.3.4. Cybernetic Modeling of CHO-S Metabolic Behavior

HCM approach was used for the final dynamic characterization of the metabolic behavior of CHO-S cells during the complete culture coupled to YSA, LYSA, PSYA, and LPSYA. Mathematical description of the cybernetic approach can be found on Ramkrishna et al. reports (Kompala et al., 1986; Varner and Ramkrishna, 1999; Ramkrishna and Song, 2012; Song and Ramkrishna, 2012). Briefly, this approach states that each EM can be simplified as the consumption of one or more substrates (*S*_*i*_) catalyzed by a critical enzyme (*E*_*i*_) to produce biomass and other products. Recursively, *E*_*i*_ synthesis is upregulated by the presence of its specific substrate *S*_*i*_. Therefore, it can be written :

(27) $$\begin{array}{l} S_{i}+X\overset{E_{i}}{\to }(1+Y_{x/s})X+\ldots \end{array}$$

(28) $$\begin{array}{l} S_{i}+X\overset{S_{i}}{\to }X^{\prime }+E_{i}+\ldots \end{array}$$

Where *X*′ represents the biomass excluding the critical enzyme *E*_*i*_. These two reactions can be described kinetically by Michaelis–Menten equations, which are commonly used to describe enzymatic catalysis, as stated below:

(29) $$\begin{array}{l} r_{i}=\frac{k_{i}e_{i}s_{i}X}{K_{i}+s_{i}} \end{array}$$

(30) $$\begin{array}{l} r_{E_{i}}=\frac{\alpha s_{i}X}{K_{i}^{\prime }+s_{i}} \end{array}$$

By introducing growth, dilution and enzymatic decay, the rate to the kinetic description can be described as:

(31) $$\begin{array}{l} \frac{de_{i}}{dt}=\frac{\alpha_{i}s_{i}}{K_{i}^{\prime }+s_{i}}u_{i}+\frac{d}{dt}\left(lnX\right)e_{i}-\beta_{i}e_{i} \end{array}$$

where α_*i*_ and β_*i*_ are the production and decay constants of the enzyme and have been calculated for various microorganisms and cell lines (Kompala et al., 1986; Ramkrishna and Song, 2012). *e*_*i*_ is the specific concentration of the enzyme *e*_*i*_ such that *e*_*i*_*X* is the total concentration of this enzyme. α is the maximum synthesis rate for this enzyme and *k*_*i*_*e*_*i*_ substitutes the maximum flux rate of the classic Michaelis–Menten equation. Then, cybernetic modeling solves the difficulty of calculating *e*_*i*_, by the assumption that the maximum rate is defined by the maximum quantity of enzyme that can be present in the biomass. Therefore:

(32) $$\begin{array}{l} k_{i}^{max}=k_{i}e_{i}^{max} \end{array}$$

(33) $$\begin{array}{l} e_{i}^{max}=\frac{\alpha_{i}}{k_{i}^{max}+\beta_{i}} \end{array}$$

within these equations, it can be deduced that the enzyme concentration value can be substituted by a relative enzyme value respective to the maximum enzyme concentration given that:

(34) $$\begin{array}{l} k_{i}e_{i}=k_{i}^{max}=\left[\frac{e_{i}}{e_{i}^{max}}\right] \end{array}$$

Finally, the cybernetic modeling introduces the regulation of the inhibition/activation of enzyme expression and repression/induction of enzyme activity by the introduction of the variables υ y ν which regulate enzyme synthesis *r*_*E*_*i*__ and activity *r*_*i*_ along the model. This is made such that:

(35) $$\begin{array}{l} \frac{dE_{i}}{dt}=r_{E_{i}}\upsilon_{i}\;\left(0<\upsilon_{i}<1\;;\;\underset{i}{\sum }\upsilon_{i}=1\right) \end{array}$$

(36) $$\begin{array}{l} \frac{dM_{i}}{dt}=\sum r_{i}\nu_{i}\;\left(0\leq \nu_{i}\leq 1\right) \end{array}$$

The cybernetic variables υ and ν are calculated with the use of matching law equations constructed for specific metabolic objectives such as growth, carbon consumption, oxygen consumption or others. In this way the cybernetic variables can compare the “returns” of each EM and regulate its participation on metabolism across time. The equations used for the cybernetic variables are the following:

(37) $$\begin{array}{l} \upsilon_{i}=\frac{R_{i}}{\sum_{j}R_{j}} \end{array}$$

(38) $$\begin{array}{l} \nu_{i}=\frac{R_{i}}{max_{j}\left(R_{j}\right)} \end{array}$$

where *R*_*i*_ represents the return of each alternative EM calculated from the metabolic objective. In this way, dynamic flux distribution can be calculated to describe metabolic and physiologic behavior and characteristics given by a metabolic network (Kompala et al., 1986; Varner and Ramkrishna, 1999; Ramkrishna and Song, 2012, 2016).

In this work, each cybernetic approach was implemented using two EM selected families constructed by YSA, LYSA, PSYA, and LPSYA performed with yields calculated at the start of the culture (first family, GLC preferential consumption) and time of maximum LAC consumption rate (second family). Cybernetic modeling was then performed as reported by Ramkrishna et al. (Kompala et al., 1986; Varner and Ramkrishna, 1999; Ramkrishna and Song, 2012). The initial relative enzyme concentration ratio was set to 0.9 for the first EM and 0.1 for the EM of the second family. Carbon consumption was selected as the metabolic objective and calculated for each EM for all consumed metabolites. All other cybernetic model parameters for enzyme production and decay rates were set, as described by Ramkrishna et al. (Kompala et al., 1986; Song and Ramkrishna, 2009, 2012; Ramkrishna and Song, 2012). Dynamic model rates for each EM (*r*_ι_) selected were described with rate equations similar to the physiological model.

(39) $$\begin{array}{l} r_{\iota }=k_{\iota }\left(\frac{{\left[M\right]}_{\iota }}{K_{\iota }+{\left[M\right]}_{\iota }}\right) \end{array}$$

where *M*_ι_ refers to the preferential metabolite consumed for each family (GLC, LAC), *k*_ι_ refers to the maximum consumption rate on each fermentation process section. *K*_ι_ refers to the saturation constant for each selected EM. Flux rate equation parameters were approximated to experimental data using a genetic algorithm programmed by Martinez et al. (2018). Briefly, the MATLAB algorithm started with assigning *k*_ι_ and *Kι* initial values of 1 and 10, respectively, for every EM. Then, by perturbation of one parameter at a time by a random numeric factor, new parameter sets were obtained. Subsequently, the sets were used for 200 step SSE driven non-linear numeric minimization algorithms to generate new daughter *k*_ι_ and *K*_ι_ parameter model sets. From these daughter models, the set with the lowest SSE was extracted and crossed with the second-lowest SSE set by acquiring the value of its perturbed parameter (*k*_ι_ or *K*_ι_). This inter-crossed set passed onto the next generation, where another round of individual parameter perturbation was made. The algorithm was cycled until either a constant SSE was obtained or more than ten cycles were performed without finding a smaller SSE value. Finally, these parameters were subjected to a final SSE non-linear numeric minimization to model the flux rates of each EM and the final metabolic dynamic flux model for each fermentation.

### 2.4. Polar Space Analysis Predictors for Genetic Modification and Metabolic Engineering of CHO Cells

The most difficult assessment for metabolic models constructed around EM selection by experimental data, is their prediction capabilities, specifically regarding genetic modifications. This difficulty arises from the fact that no metabolic objective was used for the selection of the principal EM used on the metabolic network. This characteristic makes the straightforward selection of a new EM with reaction perturbations (downregulation, upregulation, knockouts, and more) difficult. In this work, an approach to generate new EM active sets with modified reactions was constructed with the use of the previously described polar solution space. This approach is based on the assumption that cells have redundant and highly regulated metabolic reactions, which translate to the minimal metabolic modification possible but efficient enough to adjust to new conditions, either abiotic or biotic, as used on the Minimization Of Metabolic Adjustment (MOMA) approach (Segrè et al., 2002; Shlomi et al., 2005). With this assumption, it is possible to start on the modeled and validated *EM*_ι_ and select a path of new EM, which has, at the same time, the closest distance to it and minimize or maximize an individual θ_*y*_. This path can be calculated on an iterative process replacing *EM*_ι_ by the new EM determined up until a certain arbitrary or set parameter such as $\theta_{f}^{y}=\frac{\theta_{\iota }^{y}}{2}$ or until reaction knock out which means $\theta_{f}^{y}=0$. The latter can be achieved by calculation of distances between all *EM*_*i* = ι+1…*n*_ and *EM*_ι_, and finding the next EM by:

(40) $$\begin{array}{l} EM_{\iota^{\prime }}=EM\left(\min\left[\Omega_{i}^{\theta_{i}-\theta_{\iota }}\right]{|}_{\left(i=\iota +1\right)}^{n}\right)\;\forall \;\theta_{i}<\theta_{\iota } \end{array}$$

where Ω is the subset of Φ containing only the remaining EM and ι′ refers to the newly selected EM to replace the ι one. This allows to establish a modification path toward a reaction objective set as the prediction query experiment. Changes in the selection of $\theta_{f}^{y}$ parameter would not change the path of the solution in any way, but only on the number of EM selected derived from the desired extent of the analysis. On the proof of concept presented on this work, the maximization of *v*39:*PYR*_[*c*]_ ⇒ *PYR*_[*m*]_ up to a ten-time increase was used as the query.

## 3. Results

### 3.1. Physiologic Model Construction and Analysis

**Figure 2:**
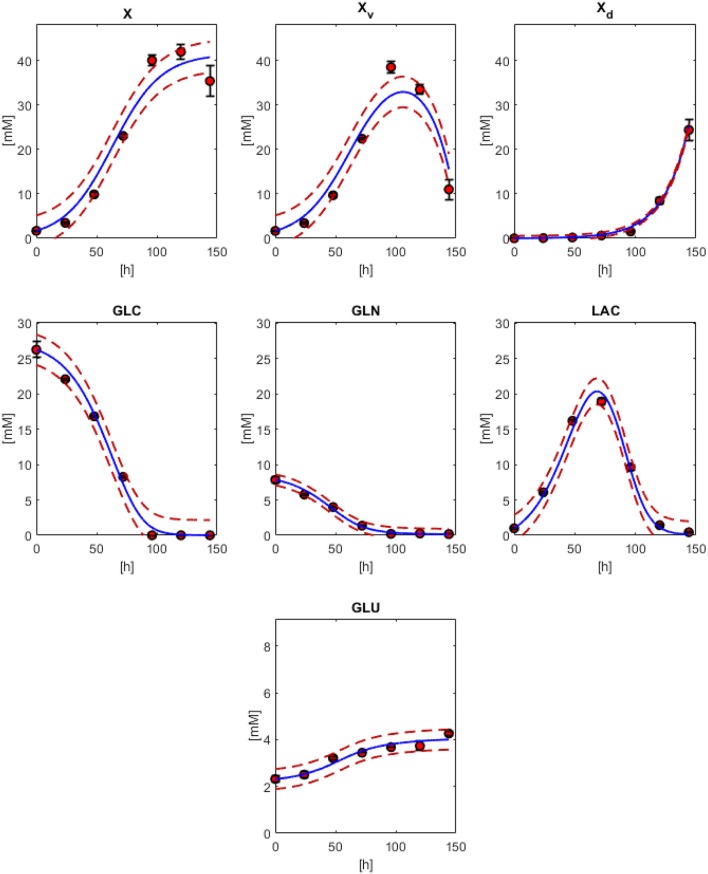
Physiological modeling results compared to experimental data. biomass models with Equations (1) (*X*), 3 (*X*_*v*_) and 2 (*X*_*d*_). Models for GLC (Equation 10), GLN (Equation 11), LAC (Equation 12), and GLU (Equation 13). Red dashed lines are the confidence curves for each model.

**Figure 3:**
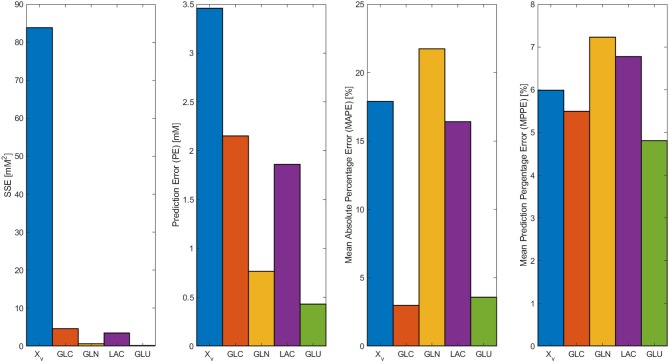
SSE, Prediction error (PE), Mean Absolute Percentage Error (MAPE), and Mean Prediction Percentage Error (MPPE) calculated for biomass, GLC, GLN, LAC, and GLU models.

In Figure 2, the model results along with the experimental data sets are presented. All models followed the experimental behavior in good agreement. All experimental points fall into the PE confidence bounds calculated except for the maximum *X*_*v*_ data point. This late fermentation error increases on biomass calculations are the result of the interaction with the simplified first-order mathematical description of cell death, which results in an overestimation on the *X*_*d*_. This modeling simplification was made to describe cell death as the best possible way without introducing non-biomass terms to the death rate equation, complicating further model calculations. However, the simplification allows for the main dissolved metabolites to be modeled in fair agreement to the experimental behavior avoiding the construction of inseparable partial differential equation systems. As can be observed in Figure 2, GLC, GLN, LAC, and GLU model and error bounds calculated comprise all experimental data points. The accuracy and error evaluation is presented in Figure 3. In these figures, *X*_*v*_ presents the highest SSE and, therefore, the highest PE of all models with values of 83.8 mM^2^ and 3.46 mM, respectively. Comparison of these values between all metabolites is difficult as they are not normalized values. Therefore, MAPE was calculated as it is one of the most utilized parameters used for measuring model accuracy (Kim and Kim, 2016). MAPE calculation produced errors below 20 % for all metabolites except for GLN, presenting a percentage up to 21.7 %. This parameter would suggest a relatively low model accuracy contrasting with the observed behaviors in Figure 3. Therefore, MPPE calculations were obtained and presented values between 10% and 5% for all models, having a similar behavior from MAPE in terms of model accuracy distribution (Figure 3), but with more consistent values with the observations of Figure 2, and without MAPE outlier percentage errors derive from normalization calculations with lower that one values (Kim and Kim, 2016). Therefore, MPPE was preferred as the model accuracy value on this report as it presented a more robust calculation on small values than MAPE, at least for these experimental calculations with many data points having lower than 1 value (Kim and Kim, 2016).

**Figure 4:**
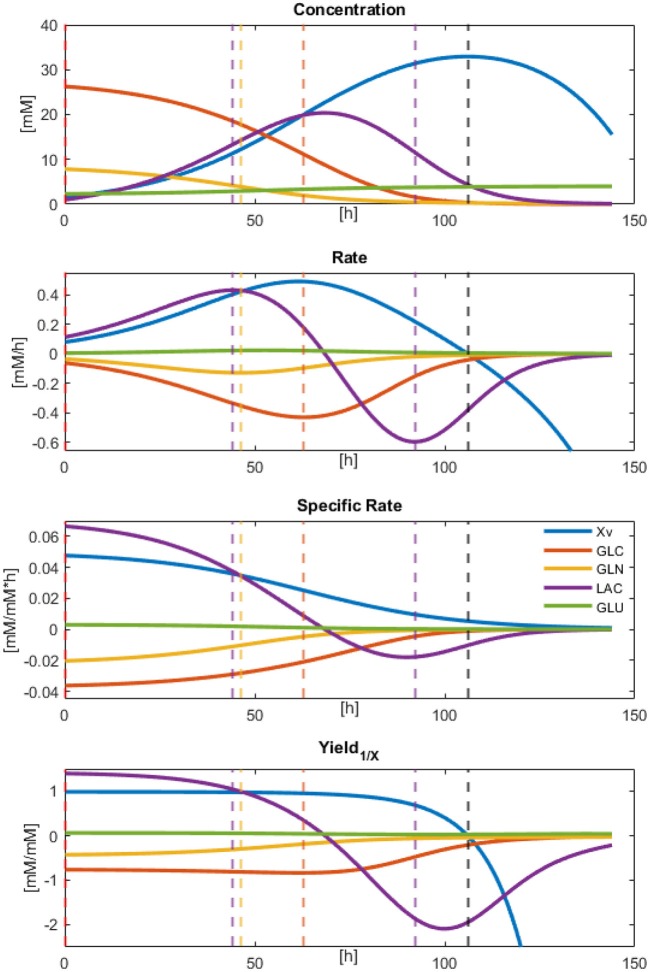
Modeled results for Concentration, Rates, Specific rates, and Yields calculated by numeric approximation with Δ*t* = 0.1.

Result observations and error estimations suggested that all models were able to describe, with acceptable accuracy, the physiological behavior of the cultured CHO-S cells. Therefore, all parameters derived and calculated from them can be used as descriptors to characterize cell line behavior. Since models were numerically integrated, approximate rate values were calculated for the biomass and the main dissolved extracellular metabolites at every Δ*t* = 0.1, from the beginning up to 144 h of culture. These calculations allowed the approximation of effective instantaneous specific rates and apparent biomass yields (using the total biomass dynamic model values). The results for the concentration, rates, specific rates, and yields against *X* for *X*_*v*_, GLC, GLN, LAC, and GLU are presented in Figure 4. In this figure, different rate maxima can be observed for different metabolites. The first process corresponds to the maximum LAC production rate at 44.3 h, closely followed by the maximum GLN consumption at 46.3 h of culture. Meanwhile, GLC maximum consumption rate was obtained at 62.9 h and found to be in the middle of the exponential growth phase. Yields were stable along most of the exponential growth phase, as expected, apart from LAC and GLN. This behavior indicates that a second metabolic state is emerging at 44 h using LAC as the main substrate. Finally, a non-growth consumption of remaining metabolites occurred before cell death was observed starting at *X*_*v*_ rate equal to zero (≈106 h), and having LAC consumption rate decreased to approximately its half. This death process was nearly immediate to substrate exhaustion and suggested high cell maintenance cost. All parameters for specific rates and statistical data from equation models can be found in Supplementary Material 2.

### 3.2. Metabolic Models Construction and Analysis

#### 3.2.1. Metabolic Network and Solution Space Analysis

The metabolic network constructed (Figure 1), is a simplified representation of the actual metabolic complexity (Supplementary Material 1). Yet, stoichiometric matrix calculations resulted in 18,110,823 EM. Expansion of network even by the addition of one more reaction resulted in an exponential growth of calculated EM incapable of being computed by the MATLAB efmtool protocol tool (Terzer and Stelling, 2008) and therefore further analyzed by the herein used computational resources (exhaustion of the 16 RAM capacity used in this work). However, calculations of more extensive networks can be performed with this software on larger computational setups (Terzer and Stelling, 2008). EM can be understood as unique, indivisible metabolic reaction compositions or states conforming the vertexes of a convex solution space for metabolic behavior of the CHO-S cells. In this work, the proposed EMA was used on the constructed network reduced by 4, 6, and 8 sequential and simple constraint sets to evaluate its selection capabilities. Constraints produced subsets with 4,032,330, 153,574, and 7,855 EM for the exponential phase, characterized by GLC and GLN consumption and biomass, LAC, GLU production. The other identified apparent metabolic state constraint composition and EM sizes can be found on Supplementary Material 1.

**Figure 5:**
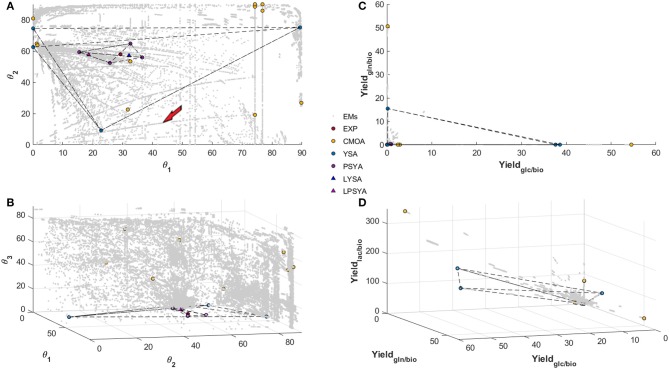
Polar Space reconstruction **(A,B)** and Yield space reconstruction **(C,D)** for EM found with the Reaction network analyzed with 6 simple constraints. Solutions found for EM Analysis with CMOA, YSA, PSYA, and Lumped LYSA and LPSYA are presented on both reconstructed spaces.

Figure 5 shows the EM set of the 6-constraint system on the convex yield solution space (Figures 5C,D) and the polar yields solution space (Figures 5A,B). A gray dot represents each calculated EM on these figures. The polar space allows an expanded visualization of the different EM, whereas, on the yield space, many of the EM are concentrated on a smaller area (Figure 5). This characteristic may allow better discrimination and selection of EM with specific traits. Furthermore, from the top representation (Figure 5), it can be observed that EM construct more explicit cluster structures or cluster-paths, containing even EM with distant discrete angular values. When these cluster-paths were studied, it was found that they consist of EM with similar characteristics, such as changes in some specific set of reactions while maintaining constant all the others. This observation means that they could be described as reaction modification pathways across the solution space and may be used for predictive reaction perturbation analyses, as will be presented and discussed further in this report.

In Figures 5A,B, θ_1_ refers to the angular calculation between GLC and GLN biomass yields, which means that as angle reaches 90° a higher ratio of GLN consumption is preferred and in the limits of 0 degrees only GLC consumption is preferred. θ_2_ represents the angular proportion for the yield of LAC production and the distribution between GLC and GLN consumption. Therefore, it shows the LAC production EM possibilities from no LAC production at 0° to the maximum output at 90°. As it can be observed, there are EM with high production of LAC on every θ_1_ substrate consumption composition, but there are more EM which relate to LAC production with GLC. More than 90 percent of EM found with LAC higher than 20° have less than 45° on θ_1_, which represents a higher GLC consumption reliance as expected. Meanwhile, θ_3_ represents the proportion of GLU production distribution to the ratio of LAC production over GLC and GLN consumption. Therefore, with higher values of θ_3_, higher distribution toward GLU production will be obtained. The EM distribution shows that higher GLU production is possible with higher GLN consumption proportion, as expected, but a tendency of reduction of GLU production with higher LAC production was also observed. Furthermore, at low LAC production (lower than 20° on θ_2_), very few EM are found.

Figure 5A reveals two different path sets, the most abundant one curving on to higher production of LAC as GLC consumption proportion rises and a less populated set (between 3 and 5 paths), that curve into lower LAC production toward higher GLC proportion. The first most abundant path set behavior was expected and readily reported in the literature. However, the second set was less anticipated, and its behavior may be against literature reports. When these latter paths were analyzed, a high GLU production with diminishing mitochondrial glutaminolysis metabolism and with LAC production reduction was observed in all EM. All of them present increasing transport from pyruvate (PYR) into the mitochondria, increasing alpha-keto-glutarate (AKG), and acetyl-CoenzymeA production and transport to the mitochondria, the latter mainly from threonine and other amino acid catabolism. These path set of EM, seem to reduce LAC production while augmenting GLC consumption proportion respective to GLN, and are characterized by a high carbon transport toward the mitochondria with an activated first half of the TCA up to AKG, transforming AKG to GLU and exporting it out of the cell, even on high GLC consumption yields. However, it is essential to note that this simplified example analysis corresponds only to a single path arbitrarily selected and signaled by the red arrow in Figure 5.

#### 3.2.2. Elementary Model Analysis and Selection for Dynamic Modeling

Within the convex solution spaces, the experimental yield position was calculated and located, as shown in Figure 5 by the red filled circles. The PSYA selected EM active set is shown in Figures 5A–D with filled purple circles. The selected EM were able to form a polyhedron containing the experimental condition data point, meaning that they may be able to describe it by a linear combination (Figure 5). The other methodologies used for EM selection are also presented, yellow filled circles for CMOA and blue filled circles for the YSA as described by Song and Ramkrishna (2009). The CMOA approach, based on metabolic objective functions minimization, presented the highest distances toward the experimental data point, and no polyhedron could be constructed containing the experimental data point (Figure 5B). In contrast, YSA was also capable of building a polyhedron containing within its volume the experimental behavior point. Moreover, the LYSA approach achieved the smallest distance to the experimental yields position on the solution space (blue triangle in Figure 5). As a comparison to this approach, the EM selected by the PSYA approach were also lumped into a virtual EM (LPSYA), and its solution presented the second closest distance to the experimental point (red triangle on Figure 5).

**Figure 6:**
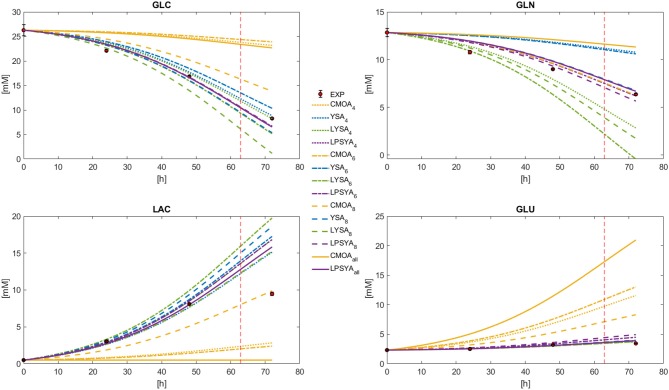
Dynamic model solution for the exponential growth phase according to Equation (26), for the best CMOA, YSA, LYSA, and LPSYA EM found during analysis on different constrained systems.

Dynamic metabolic profiles were calculated with Equation (26) for the best EM selected by each EMA in each constrained network, marked as a sub-index (Figure 6). It can be observed that the profiles derived from the EM selected by the CMOA approach do not follow the experimental behavior. The YSA approach, along with LSYA and LPSYA performed much better in these broad constraints limited systems (Figure 6). The profile shown for YSA was calculated using all its selected EM combined by the weights this approach uses for approximation to the experimental yields as described by Song and Ramkrishna (2009). The mayor deviations for these approaches were found for the GLN profile where YSA and LYSA overestimated and underestimated its consumption, respectively. LPSYA seems to be the better performing approach, not only because their profiles follow in better agreement the experimental data, but because its dispersion across the differently constrained systems was narrower than the YSA and LYSA approaches. This observation is more evident on the GLC and the GLN profiles, suggesting that the proposed PSYA approach selects very similar EM solutions within all constrained systems and therefore performs more robustly. On Figure 6 profiles, the vertical dashed line indicates the point of maximum rate of glucose consumption (Figure 4). It thus shows the shift of predominance for an alternative metabolic state, moving away from the initial exponential growth glucose-based metabolism. The EM used for all approaches in the presented comparison are the ones obtained with the information of this initial metabolic state. Therefore, it is expected that toward this dashed line (and further on), all calculated models will fall farther away from the experimental data. The latter is more evident for LAC, as cells shift to its consumption.

**Figure 7:**
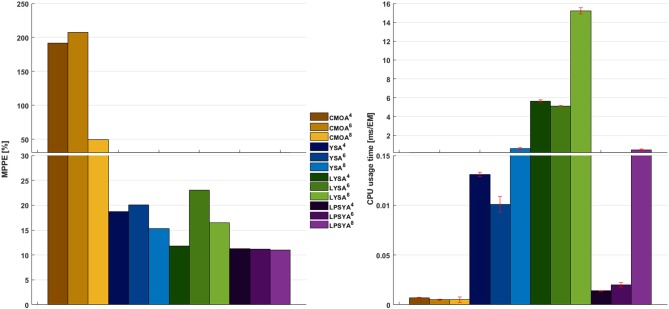
MPPE and CPU usage time over EM analyzed for the best CMOA, YSA, LYSA, and LPSYA exponential growth phase dynamic models, numerical sub-index indicates for the 4-,6-, or 8-contraint systems.

The MPPE and CPU usage times for each approach and EM set are presented in Figure 7. CMOA showed higher percentage errors (between 3 and 10 times higher than the other methods), as expected from previous observations. On the other hand, MPPE for YSA, LYSA, and LPSYA were similar, ranging within 20 to 10 %, being LPSYA the most robust with smaller MPPE deviations among all sets. However, when comparing CPU usage times, the CMOA approach presented the lowest values and exhibited high robustness along all EM constrained sets. Moreover, PSYA and CMOA derived approaches were used to calculate dynamic profiles on the EM set without any constraint (on 18,110,823 EM). Their results are presented in Figure 6 by the purple and yellow continuous lines, respectively. As can be observed, CMOA fell far away from the experimental data set, but PSYA was able to perform with similar accuracy to the results of the constrained system. Furthermore, PSYA was finished within 723.13 s (0.04 ms/EMs of CPU usage time), which is only five times higher than the lowest time determined for CMOA derived calculations. YSA and LYSA were also tried but exceeded the computational capacities. Therefore, dynamic profiles could not be obtained without constraints.

#### 3.2.3. Metabolic Hybrid Cybernetic Modeling

To better evaluate and compare the proposed EMA approach, hybrid cybernetic models were constructed for the whole culture process with the YSA, LYSA, PSYA, and LPSYA methods. Results to be presented correspond to the best performing HCMs. Presented results for YSA-HCM and LYSA-HCM were calculated using the metabolic network with eight constraints, whereas for PSYA-HCM and LPSYA-HCM, the 6-constraints system is shown. For this matter, different EMA were performed on different culture stages to obtain multiple EM to be used by the cybernetic modeling approach, as described in the section 2. Instantaneous apparent Yields and effective rates from the physiological modeling analyses, allowed the identification of two apparent mean metabolic states during culture growth: the first based on GLC consumption favoring growth and LAC production, and a second one composed on a smaller growth rate and using LAC as primary substrate (with GLC co-utilization). Yield data for these two states were calculated at the start of culture and maximum LAC consumption (44.3 and 92.1, respectively) and used for EMA-HCM construction. Constraints for the 92.1 h changed on the LAC reaction, where it was modified to its consumption (v2r > 0, see Supplementary Material 1).

**Figure 8:**
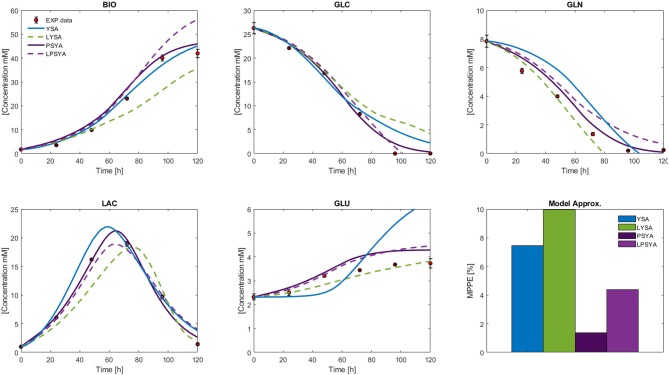
Hybrid Cybernetic Modeling Approach results for YSA, LYSA, PSYA, and LPSYA for the external measured metabolites and its SSE.

The measured external metabolites results for the HCM using YSA, LYSA, PSYA, and LPSYA are presented in Figure 8. All models displayed MPPE values smaller than 10%. YSA-HCM, PSYA-HCM, and LPSYA-HCM gave similar biomass results, but the YSA-HCM and LYSA-HCM continued biomass growth past the 100 h. In most cases, the models slightly overestimated the biomass concentration, except for the LYSA-HCM, which underestimated the biomass by presenting a low growth rate on phases consuming both GLC and LAC (over 44 h). For the GLC profiles, all models presented similar results up to ≈ 80 h where YSA-HCM and LYSA-HCM overestimated its concentration by lower consumption rates. Most of the models underestimated GLN consumption rates and therefore estimated higher GLN concentrations along the culture. LYSA-HCM and PSYA-HCM achieved the closest GLN profile to experimental data. However, in these profiles, PSYA-HCM performed best since LYSA-HCM exhausted GLN before experimental data (≈ 80 h). All models allowed LAC production and later consumption, having peaks on similar times except for LYSA-HCM, which started LAC consumption at later times. This characteristic is related to the underestimation of biomass concentration and growth, with lower GLC consumption and higher GLN consumption. The latter impacts significantly on the GLU produced, where LYSA-HCM and YSA-HCM presented very different profiles, being more evident within the YSA-HCM where the overestimation is about double of the concentration of the experimental data point at 72 h. On its part, LYSA-HCM presents an almost linear GLU production from 20 to 72 h, achieving the closest final titter concentration of all models. However, this behavior slightly underestimated the GLU concentration along the culture since most of it was produced before 44 h, meaning that ≈63 % of all GLU was produced on this first third of culture time. In contrast, the PSYA-HCM and LPSYA-HCM followed GLU experimental data with better accuracy on this same time frame but overestimated its production on the following culture hours resulting in an almost 0.5 mM excess at 72 h. However, regarding all modeled metabolites, PSYA-HCM and LPSYA-HCM seem to have the closest approximations to experimental data presenting lower MPPE values, of about 1.38 and 4.39, respectively. And their mayor deviations were due to their underestimations on GLN consumption and GLU production overestimation.

**Figure 9:**
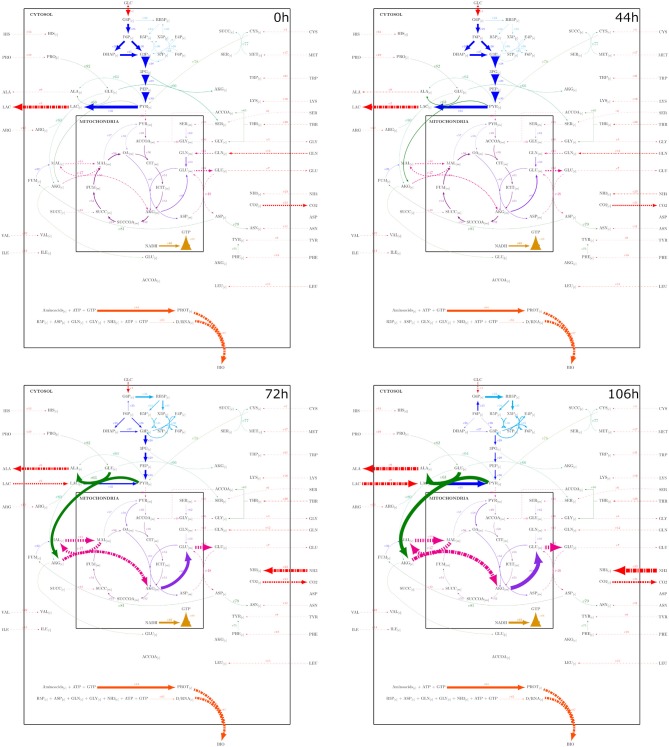
Metabolic fluxes modeled with the PSYA-Hybrid Cybernetic Model across 4 different experimental times: 0, 44, 72, and 106 h, corresponding to important stages identified by the physiological modeling step. Reaction arrow width is in direct correlation with flux distribution normalized to *X*.

To observe if the best performing HCM (PSYA-HCM) was not only approximating to the experimental data, but also modeling plausible metabolic flux distributions, their biomass normalized flux distributions along time were calculated. Figure 9 presents the PSYA-HCM biomass normalized flux distributions at 0, 44, 72, and 106 h, where the arrow width represents higher fluxes (all data is available on Supplementary Material 3). As it can be observed, from time 0 to 44 h, metabolism remained almost constant, as also was found on the instantaneous yield distributions of the physiological models. The flux distribution characteristics were in accordance with the known metabolism characteristics of CHO cells during growth (Ahn and Antoniewicz, 2011; Wahrheit et al., 2014; Galleguillos et al., 2017). The main features found for these initial distributions were low pentose phosphate flux (either oxidative or reductive sections of this pathway), high LAC production, derived from high glycolytic flux with low PYR mitochondrial transport (Warburg effect) (Ahn and Antoniewicz, 2011; Wahrheit et al., 2014). The latter caused a low TCA activation on citrate to isocitrate flux, high conversion from pyruvate to oxaloacetate by anaplerotic reactions that depend on mitochondrial glutaminolysis for TCA second half activation and GTP/NADH production needed for growth. Amino acid catabolism remained almost inexistent for most amino acids and presented alanine production and excretion (Ahn and Antoniewicz, 2011; Wahrheit et al., 2014).

As GLC started to diminish, less glycolytic flux was observed accompanied by an increase of pyruvate transformation to alanine coupled to GLU transformation to AKG (reaction v63) from 72 h and onward (Figure 9). The latter was due to the start of LAC consumption without a PYR mitochondrial transport increase, which is known to be a common limitation (Vacanti et al., 2014; Nicolae et al., 2015). Furthermore, this resulted in a reduction of GLN consumption without the loss of GLU production since carbon seemed to be introduced to the mitochondria via the malate pump in higher proportion, converted to AKG, then GLU, and then exported back to the cytosol. These malate fluxes provided the needed carbon skeletons to maintain the TCA active (Ahn and Antoniewicz, 2011; Wahrheit et al., 2014). As GLN consumption lowered and TCA increased, it was also found that the oxidative pentose phosphate pathway increased, even with carbon cycling through the reaction converting F6P to G6P (Figure 9) (Ahn and Antoniewicz, 2011; Sengupta et al., 2011; Galleguillos et al., 2017).

### 3.3. Polar Space Analysis Predictors for Genetic Modification and Metabolic Engineering of CHO Cells

**Figure 10:**
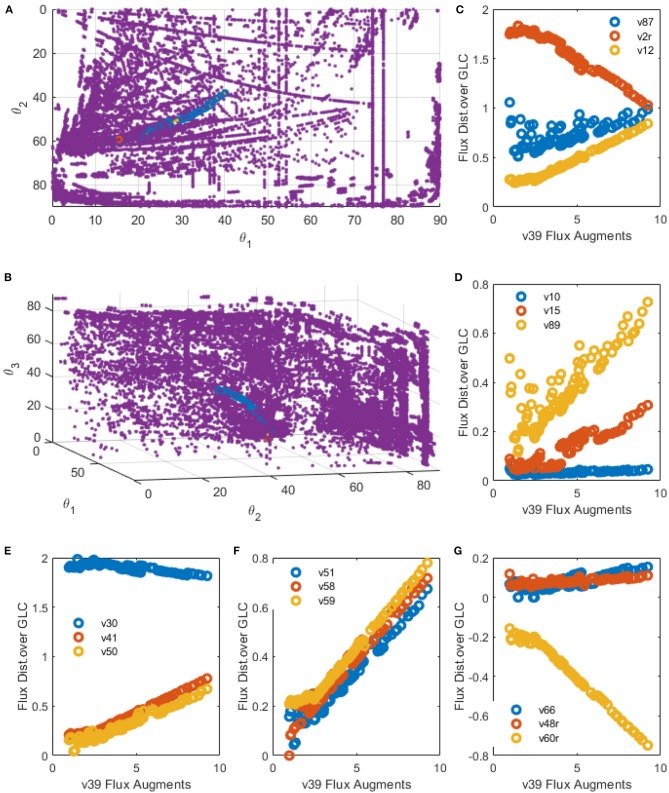
EMPA constructed for R39 increase. **(A,B)** spatial plots: Polar Space EM (purple), Initial EM selected by PSYA (Red) and minimum distance road toward R39 increase across polar Space EM selection (Blue). **(C–G)** Examples of relevant reactions found modified by R39 increase across CHO-S metabolism.

As PSYA showed good metabolic modeling performance, prediction capabilities for this approach were analyzed, as presented in the methods section. Figures 10A,B show the polar yield space and the path created by the EMPA for the increase of the mitochondrial transport of PYR. This reaction was selected as it was identified on the PSYA-HCM model as limiting for TCA activation and therefore increasing LAC production. On these Figures 10A,B, the path followed along the cluster proposes a reduction on the parameter θ_2_, which would reduce LAC production and an increase in θ_1_ and θ_3_, meaning an increase on the proportion of GLN consumption and concomitant GLU production. The latter could be related to a higher need for TCA anaplerotic reactions derived from glutaminolysis as the carbon flux entering the mitochondria as PYR increases. EMPA selected 92 alongside EM that increase the flux of R39 consecutively up to approximately ten times the initial value and reduced LAC production almost by 42% on the best scenario.

Selected reactions behavior along the R39 perturbation are presented in Figures 10C–G. Apparent linear relationships were found, giving information about distribution node modification, allowing predictive analysis to be performed as follows. LAC production was found to be reduced but only after a two-fold increase in R39 flux yield. Interestingly, below such value, some selected EM presented slightly higher LAC productions (v2r, Figure 10C). During this threshold, biomass production seems to be reduced and then increased after this same threshold value but never presenting higher production than the parental PSYA selected EM (v87, Figure 10C). Phospho-enol-pyruvate (PEP) conversion to PYR presented a slight reduction in flux distribution after the same threshold (v30, Figure 10E), suggesting lower glycolytic flux toward PYR even at high mitochondrial transport conditions. However, TCA flux presented an almost 15-fold increase (v50 and v51, Figures 10E,F) accompanied by increases on glutamine transport into the mitochondria (v41, Figure 10E). These increases were related to an equivalent increase on mitochondrial PYR transformation to oxaloacetate (OA) (v58, Figure 10F) and GLN conversion to GLU (v59, Figure 10F). These probably due to the need for increased OA and GLU conversion to AKG and ASP (v61r, Figure 10G) with the aid of the GLU-ASP mitochondrial pump (v48r, Figure 10C) which could help to maintain TCA active. Interestingly, reactions for ATP production from NADH do not increase at the same rate, showing only a two-fold increase after the two-fold increase threshold of reaction v39, and even showing a reduction before this same threshold, having the same behavior as the biomass reaction (Figure 10C). The latter suggests a limitation on the ATP production rates and NADH consumption rates, either by respiration limitations or by ADP availability on the cell.

## 4. Discussion

The physiological extracellular models presented herein were able to describe the culture behavior even for LAC, which presents a production and a later consumption phase during growth. This description was not possible to model with previously given equations for similar metabolic behaviors (acetate on *E. coli*) by Martinez et al. (2018). In this work, this was possible by modifying the model equations with Michaelis–Menten terms, which describe the specific rate behavior in relation to the concentration of key metabolites, whereas in previous reports, inequality constraints were used to limit the maximum specific rate participation (Martinez et al., 2018). This addendum to the physiological model equations previously reported resulted in a better description capability within the same modeling approach. It should be noted that CD Forti CHO complex media amino acids are consumed by CHO cells and integrated into the metabolism. However, glucose and glutamine concentrations account for 6.2 g/L, while maximum biomass is 6.4 g/L (Kurano et al., 1990), which means that these metabolites account for about 96% of the mass. Therefore, the assumption of “key metabolite(s)” driving the rates as mathematical substrates for the instantaneous specific rate determination by the Michaelis–Menten type equations should be enough to derive appropriate initial approximations, parametrize the measured metabolites and acquire an information basis toward EM selection for the metabolic modeling approach.

For all models, MAPE showed relatively high values for model-to-experiment correlations. However, it has been recognized that MAPE produces outlier percentage errors (extremely large) when the experimental or normalizing values are lower than one (Kim and Kim, 2016). This problem is intrinsic to the form of the Equation (16), where dividing by against ever going smaller values increase MAPE values more rapidly than the possible diminution of the error given by the difference of the predicted and the experimental values, presenting the tendency to produce infinite or undefined values as the experimental values approach zero. On the MAPE calculations, only GLC presented actual infinite values on the data points 144, 120, and 96 h, where GLC was already exhausted, and therefore could be ignored. Nonetheless, these MAPE characteristics explain the high GLN and LAC percentage errors, as they present many experimental values lower than 1, producing outlier errors. Many approaches for the solution of this problem can be found in the literature, mainly based on either methods to exclude outliers (most having a problem of arbitrariness) or by multiple modifications to the equation to avoid division by small values (Kim and Kim, 2016). In this report, the MPPE was used, as presented in Equation (17). This calculation solves the problem by normalizing the sum of errors by the sum of experimental values. Thus, it avoids performing divisions with lower than 1 values to normalize the error and renders comparable percentage error indicators between different metabolites. Since the same experimental data sets are used for the comparison of all EM selection approaches, any intrinsic MAPE or MPPE limitations would apply evenly for all methods. However, as the last error parameter performed more robustly than MAPE, it was preferred to qualify model accuracy for further analysis.

Modeled concentration, rates, and yield profiles suggested that GLC is the most important growth rate determinant, even in the multi-amino acid composed media (FortiCHO). This suggestion is derived from the observation that glucose consumption rates follow the Biomass rates, presenting their maxima at the same time with both values being |0.4| mM/mM*h. Even though Biomass continues to accumulate after the GLC consumption rate diminishes and other metabolites are being consumed more rapidly (e.g., LAC), its rate decays after this point. Biomass rate reduction becomes more evident as LAC re-consuming viable cells increase their contribution to the culture metabolism up to the maximum LAC consumption rate found at 92.1 h, just before GLC exhaustion. It is also essential to notice that GLN yield was reduced along with the LAC consumption, which means that LAC production may be correlated to this compound. The fact that GLC apparent yield and rate do not diminish up to ≈72 h, with already having LAC consumption metabolism activated, and furthermore the GLN apparently beginning its rate reduction (probably due to limiting concentrations), suggest that the LAC metabolic shift may be partially regulated or correlated to GLN concentration or its consumption rate. This correlation may be possible due to the known high glutaminolysis mitochondrial metabolism for TCA activation found in CHO cells (Ahn and Antoniewicz, 2011; Altamirano et al., 2013; Zagari et al., 2013).

The parameters and observations obtained from extracellular physiological modeling allowed to have useful starting information toward metabolic modeling based on the use of EM derived approaches. For EM to be useful for dynamic modeling, first, an EMA must be performed to eliminate the unnecessary or unused EM by the cells and select one or a minimal set that better describes the metabolic behavior. As a first step, a gross elimination was performed by imposing constraints on the system, such as the known presence or absence of metabolic reactions, reaction rates, metabolite consumption or production rates, growth rates, or other known parameters. Many metabolic modeling approaches have relied on constraint strategies to calculate flux distributions such as FBA, MFA, and others. However, as the metabolic network gets larger, the more underdetermined the system may become, and more parameters or constraints are needed to render meaningful solutions. Therefore, an EMA that can be performed with limited knowledge and fewer constraints should enhance dynamic metabolic modeling usage along any biotechnology and bioengineering system, and especially with CHO cells. On that regard, PSYA localizes on the Polar solution space the values of known reaction yields (or fluxes) with similar results in different ranges of constrained systems. EM and experimental solutions on PSYA are described by angles and modules instead of Cartesian coordinates, making it easier to determine similarities between EM and known yields. It is important to notice that θ_*i*_ angles represent the yield distributions of a particular reaction in relation to the plane of the previous angle θ_*i*−1_, which means that they contain the information of the reaction across the yield distribution polytope for each angle calculated from previously analyzed reactions. Their analysis can, therefore, give insights into the network characteristics and reaction node elasticity by analyzing their found polar space structure or cluster-paths properties as described for Figure 5. However, it is also important to note that even while the metabolic network is at least stoichiometrically capable of having the flux distributions described by this or any set, they may not be achievable due to cell regulation, or other kinetic limitations, thus the importance of coupling EMA to a dynamic modeling approach such as HCM.

PSYA approach achieved a smaller volume solution polyhedron than other EMA approaches such as YSA. This characteristic alone does not mean that a better dynamic modeling result must be achieved *a priori*. As it has been reported, all minimal active sets on the yield space that contain within the experimental condition point will provide similar internal flux distribution and similar dynamical simulation results (Provvost et al., 2006; Song and Ramkrishna, 2009). However, as the EM fall farther away from the experimental data point, they are more prone to individually misrepresent feasible metabolic flux states useful for cellular behavior insight. This assessment can be better observed on the Polar solution space, where one of the four selected YSA EM is composed of almost only GLN consumption, two of them of only GLC consumption and only one of them with mixed metabolism, as they all fall into highly different cluster-paths subsets of EM (Figure 5). On that matter, YSA methodology analysis is designed to obtain the larger polyhedron volume as it is more reliable and robust for describing a wider spectrum of experimental flux distribution behaviors with the same active EM set and also, for its increased calculation efficiency compared to other methodologies (which search all or many possible sets around the experimental objective) (Song and Ramkrishna, 2009). However, it may be a drawback for straightforward prediction and analysis performed with the selected EM, as all of them are representations of what the network is stoichiometrically capable, but most of them may not be achievable by the cell due to regulation or other limitations. Such problematic increases when EM numbers get higher, either because of less available constraints or by the expansion of the metabolic network, making analysis and selection more difficult. Therefore, methodologies finding efficiently smaller volume polyhedrons by closing into the experimental data set may be useful for extracting biological information regarding specific stoichiometric flux distribution given by EM.

PSYA performance was found to produce similar or better results in comparison to existing EMA strategies, as proved by the dynamic kinetic models regarding approximation capability to experimental points and the computational CPU time used. Analysis of commonly used metabolic objectives, CMOE, performed the farthest from the experimental behavior on all constraint EM sets. The latter is because the system constraints are broad, and no previous knowledge of the system was administered to assure better performance such as FBA and MFA approaches. In other words, as this approach relies on metabolic objective assumptions, such as maximization or minimization of some yields, rates or fluxes, and it requires more and more specific constraints to limit the solution space to obtain reliable solutions to the experimental behavior. This characteristic can be observed in the Figure 6 in which it is clear that while the number of constraints used increases from 4 to 8, the performance of the CMOA also increases substantially. Also, It is important to note that the constraints used in this comparison are too broad for performing FBA, as they only assert production, consumption or existence of some reactions, without constraining them to some values of the flux vector. The latter is important since FBA derived from metabolic objectives are mostly used along with specific constraints such as respiration rates, consumption or growth rates, some production rates, and so on. However, the scope of this report is to address EM selection for dynamic metabolic modeling with the minimum amount of information possible. Approximation to data has also been used as a metabolic objective by FBA, and MFA approaches. However, the use of this objective required the calculation of all the profiles for all EM and its comparison to find the best performance, therefore increases the calculation times and computational requirements significantly. CMOA presented in this report, performed the best regarding low CPU usage times on all sets. In contrast, YSA and LYSA approaches presented CPU usage times ranging from 2 to 3 orders of magnitude higher than CMOA derived approaches. On the other hand, the PSYA approach performed with lower CPU usage times, approaching even close to CMOA used times over EM as the system grows. CPU usage times seem to increase in the 8-constraint set substantially for all EMA approaches but CMOA. This apparent steep increase may be an artifact where function and libraries loading times contribution to CPU usage time is more significant than actual calculation time for this 7,855 EM size set, which is two orders of magnitude lower than the 6-constraint set. This artifact is a result of the CPU usage time normalization against EM set size, where constraints reduce more rapidly the latter number compared to the reduction of actual CPU usage time by each EMA approach. In fact, for LPSYA 6- and 8-constraint calculations took about 4.81 and 4.36 s, a 10% reduction, meanwhile EM number reduction was *around94%*.

While CMOA may have the upper hand on retrieving information on more extensive networks, given adequate knowledge on fluxes rates or yields, YSA derived methodologies can perform better with less available parameters. However, the latter has higher computational requirements and larger calculation times, making its use on large networks demanding. There are reports presenting new methodologies and mathematical approximations toward hierarchical lumping of EM that have proved to be useful in reducing the computational demands of these approaches, making them more accessible to large network modeling (Song and Ramkrishna, 2010). Nonetheless, in this report, the novel approach for Polar yield space analysis performs with similar accuracy to the YSA and LYSA approaches while requiring much shorter calculation times and computational requirements, closing in toward CMOA and common FBA calculation time frames. Moreover, PSYA was able to perform with similar accuracy between all of the constrained systems within CPU usage times only five times higher than CMOA calculations.

Furthermore, PSYA coupled with HCM allowed having the most accurate performance to experimental data for the constructed metabolic models (Figure 8). Metabolic flux distributions were found to be in accordance with previous reports on CHO metabolism. The modeled oxidative pentose phosphate pathway increase after the reduction of GLN extracellular concentration has been reported during stationary culture phases by ^13^C MFA and has been proposed to regenerate NADPH/NADP^+^, compensate oxidative stress during oxidative growth and to cover NADPH requirements as metabolism changes from consuming amino acids to the biosynthesis of building blocks for protein production (Ahn and Antoniewicz, 2011; Sengupta et al., 2011; Wahrheit et al., 2014; Galleguillos et al., 2017). In fact, for the non-growth phase, Sengupta et al. (2011) reported high flux diversion to the pentose phosphate pathway with carbon recycling from F6P to G6P. This effect was also observed on the models constructed herein after 72 h and increased toward the 106 has GLN consumption metabolism diminished by its exhaustion (Figure 9). Finally, it was found that as growth decreased (toward 106 h), glycolytic flux through EMP was reduced, and the pentose pathway proportion augmented, producing more carbon dioxide and GLU as the TCA decreased. All the latter reactions are probably set to produce necessary energy and amino acids for maintenance before entering the death stage (Wahrheit et al., 2014).

As discussed, the PSYA-HCM approach produced reliable flux distributions, which adjusted and helped to describe the physiological and metabolic behavior of CHO-S cells under typical culture conditions. However, it is relevant for an approach to render easy and reliable information upon predicting changes in the system. For that reason, EMPA was constructed over the polar convex solution space. EMPA is sustained on the same assumption as MOMA, that regulation on the cellular machinery has the metabolic objective of minimizing change while maximizing adaptation by minimizing the distance to a previously known solution (Segrè et al., 2002; Shlomi et al., 2005). EMPA finds a perturbed EM node can be calculated using a known EM describing the behavior of the parental metabolic state and then successively search for the closest EM that augments (or reduces) the reaction that one wishes to perturb. This calculation would theoretically provide a path with the lowest metabolic modification rate (as a whole) while providing the highest unique reaction rate modification. Normally, MOMA is performed by the distance minimization for a given flux vector solution previously calculated (commonly FBA) to determine a mutant flux vector (Segrè et al., 2002; Shlomi et al., 2005). Whereas, EMPA performs a sequential search on the EM sets calculated on the polar yield space with the initial EM solution found by PSYA. The Yield Polar Space description can be made to describe and distribute EM around a particular reaction node to be studied. As the angular data for each EM on the polar space is calculated from consecutive ratios between reaction yields, its spatial position contains the information about their node yield distribution, with out losing other yields cumulative information. This spatial transformation seems to cluster EM in closely related sets. This sets also seem to provide information of node behavior, such as sequential reaction yield diminution or augment (EM paths), elasticity (when set or path breaks), or others. However, more studies have to be perform to increase the knowledge and possible analysis that could be performed on this sets and solution space. It is noteworthy that this approach is only valid (at least theoretically) under the same EM constrained set structure on the polar convex solution space (as defined in the previous section). That is because the initial assumption could fail upon changing apparent structured EM sets on the polar space. It should be emphasized that this approach was only tested as a proof of concept, while more needs to be done to describe polar space characteristics (Euclidean or non-Euclidean, clustering, nodes, dispersion among others) to devise a better prediction methodology.

Despite this, EMPA provided useful information toward metabolic and bioprocess engineering, as shown in Figure 10, and allowed to review possible reaction interactions and relationships, which may be attractive as genetic modification targets toward reducing LAC production. EMPA suggested a limitation on the ATP production rates, either by respiration limitations or by ADP availability. On that matter, Nicolae et al. (2015) presented a modeling approach for determining active elementary flux modes on mitochondria using data from selectively permeabilized CHO cells. They found that adenosine diphosphate (ADP) augmented the uptake rate of most metabolites by respiratory chain stimulation (Nicolae et al., 2015). This effect was exacerbated by media fortification with pyruvate (PYR), citrate (CIT), AKG and glutamic acid (GLU) (Nicolae et al., 2015). Furthermore, they found that Isocitrate dehydrogenase (ICdh) and the α-ketoglutarate dehydrogenase (AKGdh) were the key regulators of the tricarboxylic acid cycle (TCA) (Nicolae et al., 2015). The findings provided by the proposed EMPA agree with these reports. A similar EMPA was performed but changing the query toward LAC export minimization as a query (v2r minimization) and presented similar findings. EMPA results suggest the overexpression of reactions v47 and v44 to transport cytosolic AKG to TCA by a malate pump cycle instead of the GLN-GLU pump cycle during growth. Also suggest upregulating the v66 reaction to transport part of the glycolytic flux and remnant GLU production toward AKG, which could help reduce the LAC production without reducing growth or product yields. And finally, other amino acid catabolic reactions consuming ATP and producing AKG, succinate, and ACCOA, such as threonine, valine, or leucine reactions (v69, v72, v74) are also suggested as upregulation targets toward LAC production reduction and protein production enhancement.

## 5. Conclusions

Polar Space Yield Analysis (PSYA) can be summarized as a derived approach from the Yield Analysis presented by Song and Ramkrishna (2009). With this novel approach, it was possible to reconstruct the convex solution space and reveal, extract and define subsets of EM capable of describing CHO-S cells cultures and meaningful information and about its metabolism. The critical aspect of PSYA is that it helps to simplify the solution space description by rendering a pathway through the multi-dimensional polytope bounding important information about reaction relationships and yields. Moreover, PSYA can provide critical information about specific node distributions required for the study of reaction node elasticity, regulation, and other relevant characteristics. PSYA and LPSYA performed with high accuracy, can be coupled to dynamic HCM (Ramkrishna and Song, 2012), and can be constructed with lower CPU usage times (close to FBA analysis). Despite this, the presented approach remains to be tested on large or even genome-scale networks to better characterize its limitations.

In the case study considered in this report, PSYA-HCM and LPSYA-HCM produced dynamic metabolic models that follow with reasonable accuracy the experimental behavior of the CHO-S cell culture. Moreover, flux distributions obtained with the PSYA-HCM were in agreement with the metabolism knowledge available in the literature, presenting low pentose phosphate flux, high LAC production, and low PYR on cytosol to PYR on mitochondria conversion, low TCA activation on isocitrate dehydrogenase and high recirculation toward mitochondrial PYR by malic enzymes, while catabolism of many amino acids remained low.

The presented polar solution space also allowed to perform a perturbation analysis based on finding the closest EM sequentially to the previous parental EM, which maximizes or minimizes a particular reaction. With EMPA analysis, we were able to find and propose attractive modification reaction targets that could reduce LAC production in CHO-S cells culture. The findings regarding mitochondrial metabolism were found to agree with previous reports from other research groups, while some other not so evident targets were proposed. It is important to stress that these analyses were performed as proofs of concept for the further development of the presented PSYA, LPSYA, and EMPA approaches, being this report the first to propose a shift from Cartesian to Polar coordinates and tools for their analysis, resulting as an apparently reliable and useful approach for metabolic modeling by EM analysis.

## Data Availability Statement

The data generated or analyzed during this study are included in this published article [and its supplementary information files]. PSYA MATLAB example codes and data matrices are available at https://github.com/JuanAndresMtz/PSYA. Other requests can be obtained from the corresponding authors on reasonable request.

## Author Contributions

JM, DB, MC, LP, and OR conceived the study and designed the experiments. DB and MC carried out all culture experiments. JM constructed models, parameter calculations, and model implementations and programming. JM, DB, LP, and OR collaborated with strain and results analysis and characterization. All authors collaborated on manuscript construction and critically revised it. All authors have read and approved the final manuscript.

### Conflict of Interest

The authors declare that the research was conducted in the absence of any commercial or financial relationships that could be construed as a potential conflict of interest.

## Abbreviations

EM: Elementary Mode(s)
EMA: EM Analysis
CMOA: Common Metabolic Objective Analysis
PSYA: Polar Space Yield Analysis
LPSYA: Lumped Polar Space Yield Analysis
YSA: Yield Space Analysis
LYSA: Lumped Yield Space Analysis
MOMA: Minimization Of Metabolic Adjustment
EMPA: EM Perturbation Analysis
SSE: Sum of the Square Error
MAPE: Mean Absolute Percentage Error
PE: Prediction Error
MPPE: Mean Prediction Percentage Error
HCM: Hybrid Cybernetic Modeling.
