# Supplementary material for: Dynamic Modeling of CHO Cell Metabolism Using the Hybrid Cybernetic Approach With a Novel Elementary Mode Analysis Strategy
Source: Front Bioeng Biotechnol. 2020 Apr 15;8:279. doi: 10.3389/fbioe.2020.00279 (PMC7174696; doi:10.3389/fbioe.2020.00279)
Supplement: Supplementary file 1 [file Data_Sheet_1.PDF]

Supplementary material 1:Metabolic network definition and constraints for dynamic models

1 SUPPLEMENTARY TABLES AND FIGURES

Table S1. Metabolic network constructed for CHO metabolism modeling

Index	Reactions	Index	Reactions
	Extracellular transport reactions		Mitochondrial transport
$v1$	$GLC + ATP \rightarrow G6P_{[c]}$	$v39_r$	$PYR[c] \rightarrow PYR_{[}m]$
$v2_r$	$LAC_{[c]} \rightarrow LAC$	$v40_r$	$ACCOA[c] \rightarrow ACCOA_{[}m]$
$v3$	$ASP \rightarrow ASP_{[c]}$	$v41_r$	$GLN[c] \rightarrow GLN_{[}m]$
$v4$	$CYS \rightarrow CYS_{[c]}$	$v42_r$	$GLU[c] \rightarrow GLU_{[}m]$
$v5_r$	$GLY_{[c]} \rightarrow GLY$	$v43_r$	$SUCC[c] \rightarrow SUCC_{[}m]$
$v6$	$SER \rightarrow SER_{[c]}$	$v44_r$	$MAL[c] \rightarrow MAL_{[}m]$
$v7_r$	$GLU_{[c]} \rightarrow GLU$	$v45_r$	$SER[c] \rightarrow SER_{[}m]$
$v8$	$TYR \rightarrow TYR_{[c]}$	$v46_r$	$GLY_{[}m] \rightarrow GLY_{[}c]$
$v9_r$	$ALA_{[c]} \rightarrow ALA$	$v47$	$AKG[c] + MAL_{[}m] \rightarrow MAL[c] + AKG_{[}m]$
$v10$	$ARG \rightarrow ARG_{[c]}$	$v48_r$	$GLU[c] + ASP_{[}m] \rightarrow GLU_{[}m] + ASP[c]$
$v11$	$ASN \rightarrow ASN_{[c]}$		
$v12$	$GLN \rightarrow GLN_{[c]}$		Mitochondrial TCA
$v13$	$HIS \rightarrow HIS_{[c]}$	$v49$	$PYR_{[}m] \rightarrow ACCOA_{[}m] + CO2i + NADH$
$v14$	$ILE \rightarrow ILE_{[c]}$	$v50$	$ACCOA_{[}m] + OAC_{[}m] \rightarrow CIT_{[}m]$
$v15$	$LEU \rightarrow LEU_{[c]}$	$v51$	$CIT_{[}m] \rightarrow AKG_{[}m] + CO2i + NADPH$
$v16$	$LYS \rightarrow LYS_{[c]}$	$v52$	$AKG_{[}m] \rightarrow SUCCOA_{[}m] + CO2i + NADH$
$v17$	$MET \rightarrow MET_{[c]}$	$v53_r$	$SUCCOA_{[}m] \rightarrow SUCC_{[}m] + GTP$
$v18$	$PHE \rightarrow PHE_{[c]}$	$v54_r$	$SUCC_{[}m] \rightarrow FUM_{[}m] + FADH2$
$v19$	$PRO \rightarrow PRO_{[c]}$	$v55_r$	$FUM_{[}m] \rightarrow MAL_{[}m]$
$v20$	$THR \rightarrow THR_{[c]}$	$v56_r$	$MAL_{[}m] \rightarrow OAC_{[}m] + NADH$
$v21$	$TRP \rightarrow TRP_{[c]}$		
$v22$	$VAL \rightarrow VAL_{[c]}$		Mitochondrial anaplerotic
$v23_r$	$NH3_i \rightarrow NH3$	$v57$	$MAL_{[}m] \rightarrow PYR_{[}m] + NADPH + CO2i$
$v24_r$	$CO2_i \rightarrow CO2$	$v58$	$PYR_{[}m] + CO2i + ATP \rightarrow OAC_{[}m]$
		$v59$	$GLN_{[}m] \rightarrow GLU_{[}m] + NH3i$
	Cytosolic glycolysis	$v60_r$	$GLU_{[}m] \rightarrow AKG_{[}m] + NADPH$
$v25_r$	$G6P[c] \rightarrow F6P[c]$	$v61_r$	$ASP_{[}m] + AKG_{[}m] \rightarrow OAC_{[}m] + GLU_{[}m]$
$v26$	$F6P[c] + ATP \rightarrow DHAP[c] + G3P[c]$	$v62_r$	$SER_{[}m] \rightarrow GLY_{[}m] + NADPH$
$v27_r$	$DHAP[c] \rightarrow G3P[c]$		
$v28_r$	$G3P[c] \rightarrow 3PG[c] + NADH + ATP$		
$v29_r$	$3PG[c] \rightarrow PEP[c]$		
$v30$	$PEP[c] \rightarrow PYR[c] + ATP$		
$v31_r$	$PYR[c] + NADH \rightarrow LAC[c]$		
$v32_r$	$FUM[c] \rightarrow MAL[c]$		
	Cytosolic pentose phosphate pathway		
$v33$	$G6P[c] \rightarrow RB5P[c] + 2NADPH + CO2i$		
$v34_r$	$RB5P[c] \rightarrow R5P[c]$		
$v35_r$	$RB5P[c] \rightarrow X5P[c]$		
$v36_r$	$X5P[c] + R5P[c] \rightarrow S7P[c] + G3P[c]$		
$v37_r$	$S7P[c] + G3P[c] \rightarrow F6P[c] + E4P[c]$		
$v38_r$	$X5P[c] + E4P[c] \rightarrow F6P[c] + G3P[c]$		

Table S2. Metabolic network constructed for CHO metabolism modeling (cont.)

Index	Reaction
	Cytosolic amino acid metabolism
$v63_r$	$PYR_[c] + GLU_[c] \rightarrow ALA_[c] + AKG_[c]$
$v64_r$	$ALA_[c] \rightarrow PYR_[c]$
$v65$	$THR_[c] \rightarrow GLY_[c] + NADH + ACCOA_[c]$
$v66$	$3PG_[c] + GLU_[c] \rightarrow SER_[c] + AKG_[c] + NADH$
$v67$	$GLY_[c] \rightarrow CO2i + NH3i + NADH$
$v68$	$SER_[c] \rightarrow PYR_[c] + NH3_i$
$v69$	$THR_[c] + ATP \rightarrow SUCCOA_[m] + NADH + CO2i + NH3i$
$v70$	$TRP_[c] \rightarrow ALA_[c] + 4CO2i + 2ACCOA_[c] + 2NADH$
$v71$	$LYS_[c] + 2AKG_[c] \rightarrow$
	$2ACCOA_[c] + 2NADH + 2CO2i + 2GLU_[c] + 3NADPH + FADH2$
$v72$	$VAL_[c] + AKG_[c] + ATP \rightarrow$
	$SUCC_[c] + GTP + GLU_[c] + 2CO2i + 3NADH + FADH2$
$v73$	$ILE_[c] + AKG_[c] + ATP \rightarrow$
	$ACCOA_[c] + SUCC_[c] + GTP + GLU_[c] + CO2i + 2NADH + FADH2$
$v74$	$LEU_[c] + AKG_[c] + ATP + GTP \rightarrow$
	$3ACCOA_[c] + GLU_[c] + CO2i + NADH + FADH2$
$v75$	$PHE_[c] + NADH \rightarrow TYR_[c]$
$v76$	$TYR_[c] + AKG_[c] \rightarrow FUM_[c] + 2ACCOA_[c] + SUCC_[c] + GLU_[c] + CO2i$
$v77$	$MET_[c] + SER_[c] + 3ATP \rightarrow CYS_[c] + SUC_[c] + GTP + NADH + CO2i + NH3i$
$v78$	$CYS_[c] \rightarrow PYR_[c] + NH3_i$
$v79_r$	$ASN_[c] \rightarrow ASP_[c] + NH3_i$
$v80$	$ARG_[c] + AKG_[c] \rightarrow 2GLU_[c] + NADPH$
$v81$	$ASP_[c] + 2ATP \rightarrow FUM_[c]$
$v82$	$PRO_[c] \rightarrow GLU_[c] + NADPH$
$v83$	$HIS_[c] \rightarrow GLU_[c] + NH3_i$
	Protein Synthesis
$v84$	$0.078ALA_[c] + 0.019CYS_[c] + 0.053ASP_[c] + 0.063GLU_[c] + 0.039PHE_[c] + 0.072GLY_[c]$
	$+0.023HIS_[c] + 0.053ILE_[c] + 0.059LYS_[c] + 0.091LEU_[c] + 0.023MET_[c] + 0.043ASN_[c]$
	$+0.052PRO_[c] + 0.042GLN_[c] + 0.051ARG_[c] + 0.068SER_[c] + 0.059THR_[c] + 0.066VAL_[c]$
	$+0.014TRP_[c] + 0.032TYR_[c] + 4ATP \rightarrow PROT_[c]$
	DNA and RNA Synthesis
$v85$	$R5P_[c] + 1.285ASP_[c] + 1.43GLN_[c] + 0.5GLY_[c] + 0.5NH3i + 7.145ATP + 0.285GTP$
	$\rightarrow DRNA[c] + 0.785FUM[m]$
	Biomass formation and death
$v87$	$0.9226PROT[c] + 0.0182DRNA[c] \rightarrow BIO$
	Energy Reactions
$v88_r$	$GTP \rightarrow ATP$
$v89$	$2NADH \rightarrow 5ATP$
$v90$	$2FADH2 \rightarrow 3ATP$

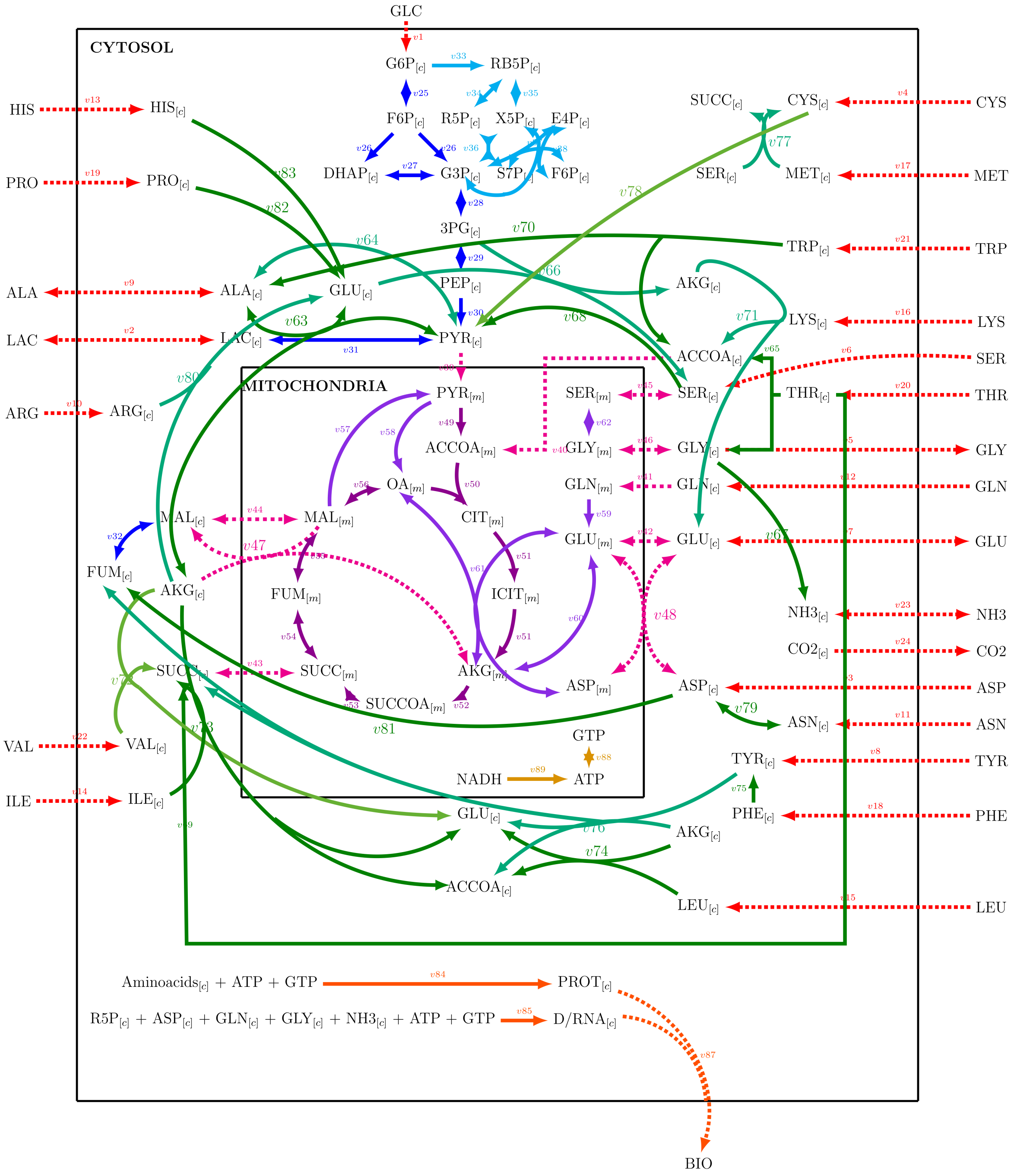

Figure S1. Reaction network used for stoichiometrical matrix construction for metabolic modeling.

Table S3. Metabolic network constraints and Elementary Mode subset sizes

GLC main consumption phase									
Constraints	EMS	BIO	LAC	GLU	GLN	v39	v53r	v41	v30
No	18,110,823	-	-	-	-	-	-	-	-
4	4,032,330	> 0	> 0	> 0	< 0	-	-	-	-
6	153,574	> 0	> 0	> 0	< 0	> 0	> 0	-	-
8	7,855	> 0	> 0	> 0	< 0	> 0	> 0	> 0	> 0
LAC main consumption phase									
No	18,110,823	-	-	-	-	-	-	-	-
4	2,129,710	= 0	< 0	>= 0	<= 0	-	-	-	-
6	164,737	= 0	< 0	>= 0	<= 0	> 0		-	-
8	99	= 0	< 0	>= 0	<= 0	> 0	> 0	> 0	> 0

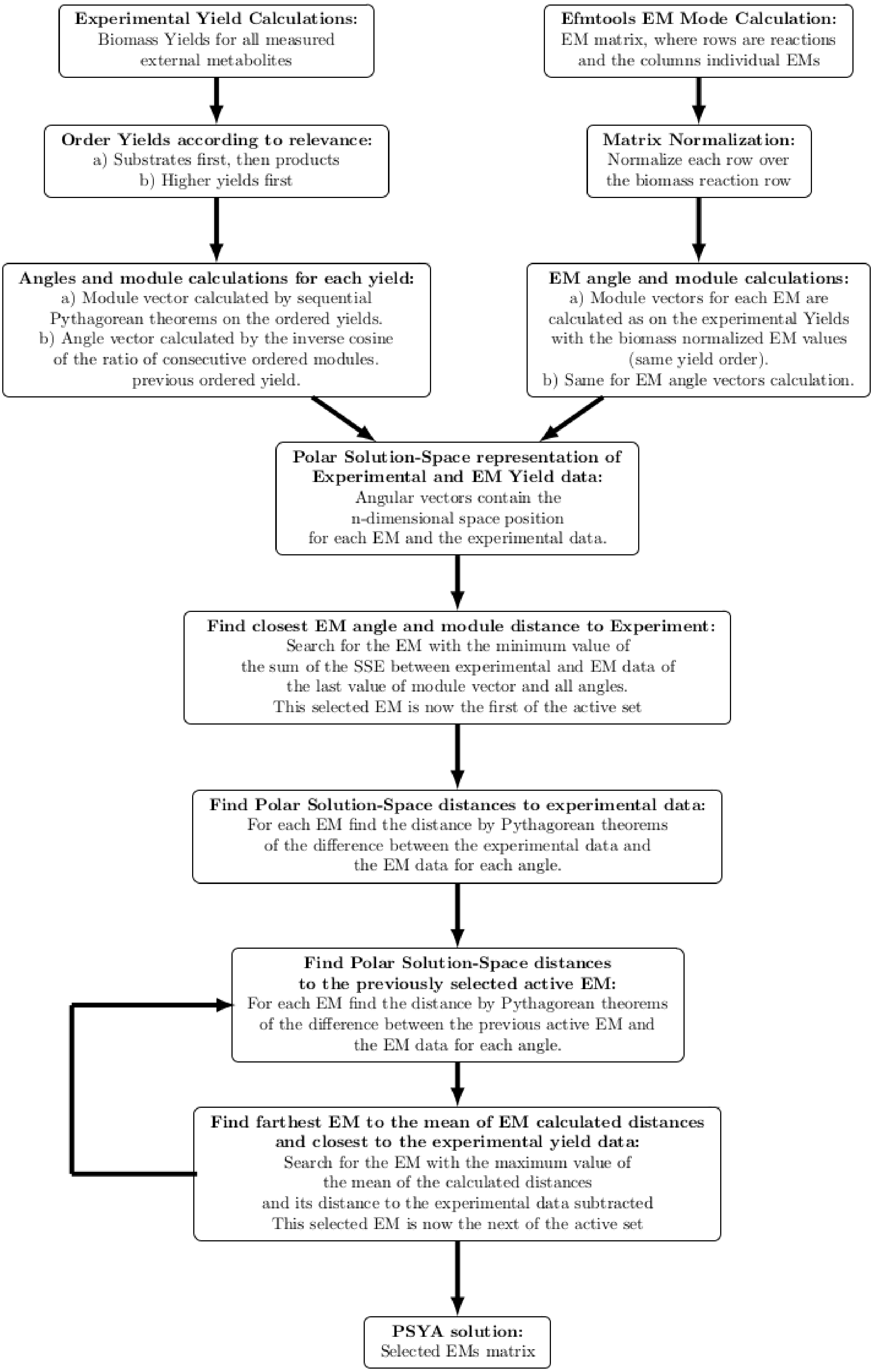

Figure S2. Algorithmic implementation diagram used for the presented PSYA (Descriptions, pt1.)

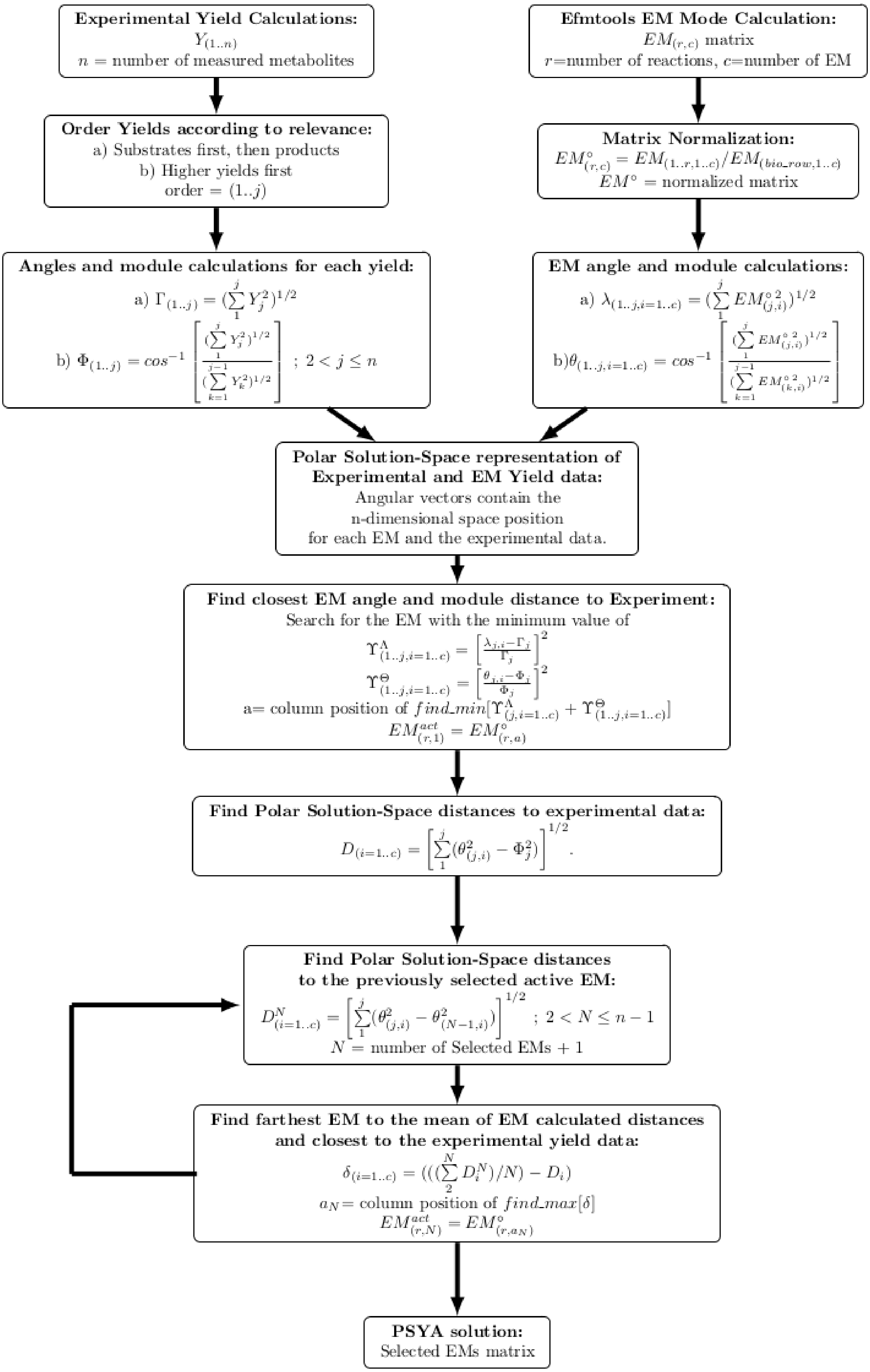

Figure S3. Algorithmic implementation diagram used for the presented PSYA (Calculations,pt2.)

```matlab
%*************************************************************************
%**** Polar Space Yield Analysis (PSYA) program solution example ********
%*************************************************************************
%                          by J.A.Martinez, L.A.Palomares and O.T.Ramirez
%                                          contact:andres.amdg@gmail.com
%*************************************************************************
% THIS IS AN EXAMPLE CODE ON MATLAB FOR THE USE OF THE PSYA APPROACH
% IT WAS MADE FOR SOLVING THE SUBMITED PRESENTED DATA BY MARTINEZ ET. AL
% IT IS IMPLEMENTED FOR CCM NETWORK CHO-S CELL ELEMENTARY MODE SELECTION
% ITS USE IS HEREIN AUTHORIZED WITH OR WITHOUT MODIFICATIONS
% EITHER ON THE SAME NETWORK, OTHER SYSTEMS OR OTHERS INTENDED METABOLIC
% MODELING NEEDS, PLEASE CITE THE IF TOTAL OR FRACTIONAL SECTIONS
% USED OR MODIFIED. IF YOU NEED MORE DATA PLEASE CONTACT US.
%*************************************************************************
%*************************************************************************


%%%%%%%%%%%%%% Initial Data allocation and normalization %%%%%%%%%%%%%%%%%
% EmSoftwaretool was used for EM calculation
% EM Matrix Loading from Efmtools software is performed on this script
% with its output diverted to the following variables:
%  efmMatrix.int_met = names of intracellular metabolites (array)
%  efmMatrix.ext_met = names of extracellular metabolites (array)
%  efmMatrix.react_name = reaction names (array)
%  efmMatrix.st = stoichiometric matrix (rows correspond to internal
%                 metabolites, columns to reactions)
%  efmMatrix.ext = same structure as st, but rows correspond to
%                  external metabolites
%  efmMatrix.ems = Elementary modes data (EMs), (rows correspond to
%                  reactions, columns to EM)
%  efmMatrix.ext_out = EMs output matrix (efmMatrix.ext*efmMatrix.ems)
%%%%%%%%%%%%%%%%%%%%%%%%%%%%%%%%%%%%%%%%%%%%%%%%%%%%%%%%%%%%%%%%%%%%%%%%%%

%** Matrix Loading *******************************************************

%%% matrices EM_Matrix,EM_Matrix_4, EM_Matrix_6 and EM_Matrix_8
%%% correspond to the efmtools output, and the reduced EM matrices outputs
%%% by 4, 6 and 8 constraints respectively.
load('EM_Matrix_6.mat') %%% efmMatrix structs as described previously.

%** Matrix Biomass Normalization******************************************

% array from 1 to number of EMs
k=1:numel(efmMatrix.ext_out(1,:));

% Normalization of EM rows against biomass row (55 on presented work)
Norm.ems(:,k)=efmMatrix.ems(:,k)./efmMatrix.ems(55,k);

% Normalization of ext_out against biomass row (25 on presented work)
Norm.ext_out(:,k)=efmMatrix.ext_out(:,k)./efmMatrix.ext_out(25,k);

efmMatrix.ems=Norm.ems; % Replace Matrix data
efmMatrix.ext_out=Norm.ext_out; % Replace Matrix data
clear Norm k %%% Clear no further used variables and struct

%***Modules and angles according to experimental Yields parametrization***
```

```
% Four experimental yields were used on this report; they were derived
% of physiological extracellular modeling.
% This section can be extended to the number of yields experimentally
% determined, Order can be changed according to relevance determined by
% experimenter. In this report, order was set by Substrate > Products and
% within them ordered by biomass yields; therefore, the order was:
% Glucose, Glutamine, Lactate, and Glutamate.
%**************************************************************************

% Experimental Yields definition found by physiological characterization
Yg=0.808; % Glucose Yield
Yn=0.308; % Glutamine Yield
Yl=1.040; %  Lactate Yield
Yu=0.054; %  Glutamate Yield
% ... continue as fit

Y=sqrt((Yg)^2+(Yn)^2+(Yl)^2+(Yu)^2); % Experimental module
y1=sqrt((Yg)^2);  % 1-dimensional module
y2=sqrt((Yg)^2+(Yn)^2);  % 2-dimensional module
y3=sqrt((Yg)^2+(Yn)^2+(Yl)^2);  % 3-dimensional module
y4=sqrt((Yg)^2+(Yn)^2+(Yl)^2+(Yu)^2);  % 4-dimensional module
% ... continue as fit

Phy_1=acosd(y1/y2); % angle over 1-dimensional line
Phy_2=acosd(y2/y3); % angle over 2-dimensional plane
Phy_3=acosd(y3/y4); % angle over 3-dimensional plane
% ... continue as fit

%********** Modules and angles according to EM Yields calculation*********
% IN THIS WORK .ems were used but .ext_out can also be used
% changing their respective indexes, as only one transport
% reaction is used for each metabolite meaning that .ext_out and their
% respective .ems is the same if the stoichiometry is also 1:1
%**************************************************************************

i=1:numel(efmMatrix.ems(1,:)); % array from 1 to number of EMs

% All dimensional Module (for all measured species)
    M(i,1)=sqrt(efmMatrix.ems(1,i).^2+efmMatrix.ems(9,i).^2+efmMatrix.ems(58,i).↙
^2+efmMatrix.ems(59,i).^2);
% 1-dimensional module (Glucose row index number 1, in this work)
    m1(i,1)=sqrt(efmMatrix.ems(1,i).^2);
% 2-dimensional module (Glutamine row index number 9, in this work)
    m2(i,1)=sqrt(efmMatrix.ems(1,i).^2+efmMatrix.ems(9,i).^2);
% 3-dimensional module (Lactate row index number 58, in this work)
    m3(i,1)=sqrt(efmMatrix.ems(1,i).^2+efmMatrix.ems(9,i).^2+efmMatrix.ems(58,i).^2);
% 4-dimensional module (Glutamate row index number 59, in this work)
    m4(i,1)=sqrt(efmMatrix.ems(1,i).^2+efmMatrix.ems(9,i).^2+efmMatrix.ems(58,i).↙
^2+efmMatrix.ems(59,i).^2);
% ... continue as fit

Theta_1(i,1)=acosd(m1(i)./m2(i)); % angle over 1-dimensional line
Theta_2(i,1)=acosd(m2(i)./m3(i)); % angle over 2-dimensional line
Theta_3(i,1)=acosd(m3(i)./m4(i)); % angle over 3-dimensional line
% ... continue as fit
```

```
%************Find closest EM to Yield Experimental Data *****************
% Other equations to stablish difference to experimental data might be
% used in this work we used the described below
%*************************************************************************

%%% Find distances to Experimental data
MSE=((M-Y)./Y).^2; % All dimensional Module Squared Error (SE)
Theta_1_SE=((Theta_1-Phy_1)./Phy_1).^2; % 1-dimensional Angle SE
Theta_2_SE=((Theta_2-Phy_2)./Phy_2).^2; % 2-dimensional Angle SE
Theta_3_SE=((Theta_3-Phy_3)./Phy_3).^2; % 3-dimensional Angle SE
% ...  continue as fit

%%% Order results from closest to farthest
SCE=MSE+Theta_1_SE+Theta_2_SE+Theta_3_SE; % Sum of Calculated SE
[PosValue,PosIndex]=sort(SCE,'ascend'); % Sort in ascending order

Orderer_M=M(PosIndex);
Ordered_Theta_1=Theta_1(PosIndex); % 1-dimensional oredered Angles
Ordered_Theta_2=Theta_2(PosIndex); % 2-dimensional oredered Angles
Ordered_Theta_3=Theta_3(PosIndex); % 3-dimensional oredered Angles
% ... continue as fit

% Establishment closest EM and the polyhedron around experimental data
i=1:numel(Ordered_Theta_1); % array from 1 to number of EMs
D(i)=sqrt((Ordered_Theta_1(i)-Phy_1).^2+(Ordered_Theta_2(i)-Phy_2).^2+(Ordered_Theta_3(i)↙
-Phy_3).^2); % Distance from each EMs to Experimental data on the angular solution space↙
(Pythagorean)
Pos1=1; % The closest EMs the closest must be the first position on the Ordered data set;↙
therefore, its position is the first.

i=1:numel(D); % array from 1 to number of distances (EMs)
DV(i)=sqrt((Ordered_Theta_1(i)-Ordered_Theta_1(1)).^2+(Ordered_Theta_2(i)-Ordered_Theta_2↙
(1)).^2+(Ordered_Theta_3(i)-Ordered_Theta_3(1)).^2); % Distance from each EMs to the↙
first selected EM on the angular solution space (Pythagorean)
Rdd=(((DV+D(1))./2)-D); % Establishment of an index for maximum distance from the mean of↙
the selected EMs and the minimum distance to the experimental data
Rdd(1)=NaN; % first position is the first selected EMs, so it is removed from the search
Pos2=find(Rdd==max(Rdd)); % The second selected EMs is the maximum index value

DV2(i)=sqrt((Ordered_Theta_1(i)-Ordered_Theta_1(Pos2(1))).^2+(Ordered_Theta_2(i)-↙
Ordered_Theta_2(Pos2(1))).^2+(Ordered_Theta_3(i)-Ordered_Theta_3(Pos2(1))).^2); %↙
Distance from each EMs to the second selected EM on the angular solution space↙
(Pythagorean)
Rdd2=(((DV2+DV)./2)-D); % Establishment of an index for maximum distance from the mean of↙
the selected EMs and the minimum distance to the experimental data
Rdd2(Pos1)=NaN; % the first selected EMs is removed from the search
Rdd2(Pos2)=NaN; % the second selected EMs is removed from the search
Pos3=find(Rdd2==max(Rdd2)); % The third selected EMs is the maximum index value

DV3(i)=sqrt((Ordered_Theta_1(i)-Ordered_Theta_1(Pos3(1))).^2+(Ordered_Theta_2(i)-↙
Ordered_Theta_2(Pos3(1))).^2+(Ordered_Theta_3(i)-Ordered_Theta_3(Pos3(1))).^2); %↙
Distance from each EMs to the third selected EM on the angular solution space↙
(Pythagorean)
Rdd3=(((DV3+DV2+DV)./3)-D); % Establishment of an index for maximum distance from the↙
mean of the selected EMs and the minimum distance to the experimental data
Rdd3(Pos1)=NaN; % the first selected EMs is removed from the search
```

```
Rdd3(Pos2)=NaN; % the second selected EMs is removed from the search
Rdd3(Pos3)=NaN; % the third selected EMs is removed from the search
Pos4=find(Rdd3==max(Rdd3)); % The fourth selected EMs is the maximum index value
clear i

%********Selected EM matrix by PSYA approach construction ****************

Positions=[PosIndex(Pos1(1)),PosIndex(Pos2(1)),PosIndex(Pos3(1)),PosIndex(Pos4(1))]; %↙
Selected EM positions on initial Efmtool EM matrices

%%%%%%%%%%%%%%%% PSYA selected EM matrix struct%%%%%%%%%%%%%%%%%%%%%%%%%%%
PSYAMatrix.ext_met=efmMatrix.ext_met; %%% External metabolites names
PSYAMatrix.react_name=efmMatrix.react_name; %%% Reaction names
PSYAMatrix.st=efmMatrix.st; %%% Internal stoichiometric matrix
PSYAMatrix.sx=efmMatrix.ext; %%% Output stoichiometric matrix
l=1:numel(Positions); %%%  array from 1 to number of selected EMs
PSYAMatrix.ems(:,l)=efmMatrix.ems(:,Positions(l)); %%%%  Selected elementary modes data↙
(EMs), (rows correspond to reactions, columns to EM)
PSYAMatrix.ext_out(:,l)=efmMatrix.ext_out(:,Positions(l)); %%%%  Selected EMs output↙
matrix (efmMatrix.ext*efmMatrix.ems);
clear l %%% clear of further unused variables

%%%%%%%%%%%%%%%% Lumping of Selected EMs LPSYA %%%%%%%%%%%%%%%%%%%%%%%%%%%

P=[0.25 0.25 0.25 0.25]; % initial linear participation coefficients for each EMs

% optimization of coefficients against experimental data
options=[]; % fmincon MATLAB options on blank
load('Exp_Data.mat') % load experimental data matrix (T, GLC, GLN, LAC, GLN columns)
funLO =@(P)lumpObj(P,PSYAMatrix,Exp_Data); % function to be minimized (constructed below↙
on functions section)
[aopt,fval,exitflag,output]=fmincon(funLO,P,[0,0,0,0],0,[1,1,1,1],1,[0,0,0,0],[1,1,1,1]);↙
%%% constrained minimization fmincon
P=aopt; % final linear participation coefficients for each EMs.
PSYAMatrix.Lsxz(:,1)=P(1).*PSYAMatrix.ext_out(:,1)+P(2).*PSYAMatrix.ext_out(:,2)+P(3).↙
*PSYAMatrix.ext_out(:,3)+P(4).*PSYAMatrix.ext_out(:,4); % Lumped elementary mode data↙
(EM), (rows correspond to reactions, columns to EM)
PSYAMatrix.Lz(:,1)=P(1).*PSYAMatrix.ems(:,1)+P(2).*PSYAMatrix.ems(:,2)+P(3).*PSYAMatrix.↙
ems(:,3)+P(4).*PSYAMatrix.ems(:,4); % Lumped EM output matrix (efmMatrix.sx*efmMatrix.↙
ems);

%******************Solution Space plotting******************************

figure %%% Polar Yield Space, experimental data and selected EMs plot
    scatter3(Ordered_Theta_1(1),Ordered_Theta_2(1),Ordered_Theta_3(1),'filled')
    hold on
    scatter3(Phy_1,Phy_2,Phy_3,'filled')
    scatter3(Ordered_Theta_1(Pos2(1)),Ordered_Theta_2(Pos2(1)),Ordered_Theta_3(Pos2↙
(1)),'filled')
    scatter3(Ordered_Theta_1(Pos3(1)),Ordered_Theta_2(Pos3(1)),Ordered_Theta_3(Pos3↙
(1)),'filled')
    scatter3(Ordered_Theta_1(Pos4(1)),Ordered_Theta_2(Pos4(1)),Ordered_Theta_3(Pos4↙
(1)),'filled')
    PirmA=[Ordered_Theta_1(Pos1(1)) Ordered_Theta_1(Pos2(1)) Ordered_Theta_1(Pos3(1))↙
Ordered_Theta_1(Pos1(1)) Ordered_Theta_1(Pos2(1)) Ordered_Theta_1(Pos4(1))↙
Ordered_Theta_1(Pos1(1)) Ordered_Theta_1(Pos3(1)) Ordered_Theta_1(Pos4(1))↙
```

```
Ordered_Theta_1(Pos1(1))];
    PirmB=[Ordered_Theta_2(Pos1(1)) Ordered_Theta_2(Pos2(1)) Ordered_Theta_2(Pos3(1))↙
Ordered_Theta_2(Pos1(1)) Ordered_Theta_2(Pos2(1)) Ordered_Theta_2(Pos4(1))↙
Ordered_Theta_2(Pos1(1)) Ordered_Theta_2(Pos3(1)) Ordered_Theta_2(Pos4(1))↙
Ordered_Theta_2(Pos1(1))];
    PirmG=[Ordered_Theta_3(Pos1(1)) Ordered_Theta_3(Pos2(1)) Ordered_Theta_3(Pos3(1))↙
Ordered_Theta_3(Pos1(1)) Ordered_Theta_3(Pos2(1)) Ordered_Theta_3(Pos4(1))↙
Ordered_Theta_3(Pos1(1)) Ordered_Theta_3(Pos3(1)) Ordered_Theta_3(Pos4(1))↙
Ordered_Theta_3(Pos1(1))];
    plot3(PirmA,PirmB,PirmG,'k--')
    scatter3(Ordered_Theta_1,Ordered_Theta_2,Ordered_Theta_3,'+')

figure %%% Experimental data and selected EMs plot
    scatter3(Ordered_Theta_1(1),Ordered_Theta_2(1),Ordered_Theta_3(1),'filled')
    hold on
    scatter3(Phy_1,Phy_2,Phy_3,'filled')
    scatter3(Ordered_Theta_1(Pos2(1)),Ordered_Theta_2(Pos2(1)),Ordered_Theta_3(Pos2↙
(1)),'filled')
    scatter3(Ordered_Theta_1(Pos3(1)),Ordered_Theta_2(Pos3(1)),Ordered_Theta_3(Pos3↙
(1)),'filled')
    scatter3(Ordered_Theta_1(Pos4(1)),Ordered_Theta_2(Pos4(1)),Ordered_Theta_3(Pos4↙
(1)),'filled')
    PirmA=[Ordered_Theta_1(Pos1(1)) Ordered_Theta_1(Pos2(1)) Ordered_Theta_1(Pos3(1))↙
Ordered_Theta_1(Pos1(1)) Ordered_Theta_1(Pos2(1)) Ordered_Theta_1(Pos4(1))↙
Ordered_Theta_1(Pos1(1)) Ordered_Theta_1(Pos3(1)) Ordered_Theta_1(Pos4(1))↙
Ordered_Theta_1(Pos1(1))];
    PirmB=[Ordered_Theta_2(Pos1(1)) Ordered_Theta_2(Pos2(1)) Ordered_Theta_2(Pos3(1))↙
Ordered_Theta_2(Pos1(1)) Ordered_Theta_2(Pos2(1)) Ordered_Theta_2(Pos4(1))↙
Ordered_Theta_2(Pos1(1)) Ordered_Theta_2(Pos3(1)) Ordered_Theta_2(Pos4(1))↙
Ordered_Theta_2(Pos1(1))];
    PirmG=[Ordered_Theta_3(Pos1(1)) Ordered_Theta_3(Pos2(1)) Ordered_Theta_3(Pos3(1))↙
Ordered_Theta_3(Pos1(1)) Ordered_Theta_3(Pos2(1)) Ordered_Theta_3(Pos4(1))↙
Ordered_Theta_3(Pos1(1)) Ordered_Theta_3(Pos3(1)) Ordered_Theta_3(Pos4(1))↙
Ordered_Theta_3(Pos1(1))];
    plot3(PirmA,PirmB,PirmG,'k--')

%% FUNCTIONS %%%%%%%%%%%%%%%%%%%%%%%%%%%%%%%%%%%%%%%%%%%%%%%%%%%%%%%%%%%%%%%
%%%%%%%%%%%%%%%%%%%%%%%%%%%%%%%%%%%%%%%%%%%%%%%%%%%%%%%%%%%%%%%%%%%%%%%%%%%

function f = lumpObj(P,PSYAMatrix,Exp_Data) % Function variable definition
m.ext_out(:,1)=P(1).*PSYAMatrix.ext_out(:,1)+P(2).*PSYAMatrix.ext_out(:,2)+P(3).↙
*PSYAMatrix.ext_out(:,3)+P(4).*PSYAMatrix.ext_out(:,4); %% Linear sum of selected EMs↙
function
load('Qbiom_Data','Qbiom_Data') % Loading of biomass production rate
                                % array of data of delta Bio /Delta t
                                % at delta t = 0.1
t=(0:0.1:72); % time array construction
t1=find(t==72); % find 72 hours(exponential growth section) on Qbiom_Data
FBIO=Qbiom_Data(1:t1)./0.1; % Calculate Biomass change on each time step
FBIO(1)=0; % eliminate first NaN value on Qbiom_Data

BIO(1,1)=Exp_Data(1,2); % Initial Kinetic Model data as Experimental data
GLC(1,1)=Exp_Data(1,3); % Initial Kinetic Model data as Experimental data
GLN(1,1)=Exp_Data(1,4); % Initial Kinetic Model data as Experimental data
LAC(1,1)=Exp_Data(1,5); % Initial Kinetic Model data as Experimental data
GLU(1,1)=Exp_Data(1,6); % Initial Kinetic Model data as Experimental data
```

```
% steps additions according to biomass growth and Selected EMs yield
% calculations for each metabolite Loop
for i=2:numel(FBIO)
GLC(i,1)=GLC(i-1,1)+m.ext_out(1,1).*FBIO(i).*0.1; %
LAC(i,1)=LAC(i-1,1)+m.ext_out(2,1).*FBIO(i).*0.1; %
GLU(i,1)=GLU(i-1,1)+m.ext_out(7,1).*FBIO(i).*0.1; %
GLN(i,1)=GLN(i-1,1)+m.ext_out(12,1).*FBIO(i).*0.1; %
BIO(i,1)=BIO(i-1,1)+m.ext_out(25,1).*FBIO(i).*0.1; %
end

% Find the positions of same modeled times and experimental time data
T=Exp_Data(1:3,1);
ExPos=zeros(numel(T),1);
for i=1:numel(T)
ExPos(i)=find(t==T(i));
end

% Comparison of experimental data and modeled metabolites on each data
% time point
BIOSE(:,1)=atand((BIO(ExPos,1)-Exp_Data(1:3,2))./Exp_Data(1:3,2));
BIOSSE(1)=abs((1/numel(T))*nansum(BIOSE(:,1)));
GLCSE(:,1)=atand((GLC(ExPos,1)-Exp_Data(1:3,3))./Exp_Data(1:3,3));
GLCSSE(1)=abs((1/numel(T))*nansum(GLCSE(:,1)));
GLNSE(:,1)=atand((GLN(ExPos,1)-Exp_Data(1:3,4))./Exp_Data(1:3,4));
GLNSSE(1)=abs((1/numel(T))*nansum(GLNSE(:,1)));
LACSE(:,1)=atand((LAC(ExPos,1)-Exp_Data(1:3,5))./Exp_Data(1:3,5));
LACSSE(1)=abs((1/numel(T))*nansum(LACSE(:,1)));
GLUSE(:,1)=atand((GLU(ExPos,1)-Exp_Data(1:3,6))./Exp_Data(1:3,6));
GLUSSE(1)=abs((1/numel(T))*nansum(GLUSE(:,1)));

% Construction of index function output to minimize
f(1)=(BIOSSE(1)+GLCSSE(1)+GLNSSE(1)+LACSSE(1)+GLUSSE(1))/5;
end
```
