# Supplementary material for: Dynamic Modeling of CHO Cell Metabolism Using the Hybrid Cybernetic Approach With a Novel Elementary Mode Analysis Strategy
Source: Front Bioeng Biotechnol. 2020 Apr 15;8:279. doi: 10.3389/fbioe.2020.00279 (PMC7174696; doi:10.3389/fbioe.2020.00279)
Supplement: Supplementary file 2 [file Data_Sheet_2.PDF]

Supplementary material 2: Physiological Model data, parameters and statistical values

1 SUPPLEMENTARY TABLES AND FIGURES

Table S1. Initial CHO culture data parameters

Variable	Value	Std.dev	Units
$T_{(0)}$	0.00	$\pm$0.00	h
$T_{(f)}$	144.00	$\pm$0.00	h
$X_{(0)}$	1.70	$\pm$0.07	mM
$X_{(f)}$	35.39	$\pm$3.48	mM
X^{max}	42.12	$\pm$1.57	mM
$Xv_{(0)}$	1.69	$\pm$0.06	mM
$Xv_{(f)}$	11.01	$\pm$2.22	mM
Xv^{max}	38.50	$\pm$1.33	mM
$Xd_{(0)}$	0.02	$\pm$0.01	mM
$Xd_{(f)}$	24.38	$\pm$2.31	mM
Xd^{max}	24.38	$\pm$2.31	mM
$GLC_{(t0)}$	26.27	$\pm$1.12	mM
$GLC_{(f)}$	0.00	$\pm$0.00	mM
ΔGLC	-26.27	$\pm$1.12	mM
$GLN_{(t0)}$	7.84	$\pm$0.43	mM
$GLN_{(f)}$	0.17	$\pm$0.02	mM
ΔGLN	-7.68	$\pm$0.41	mM
$LAC_{(t0)}$	0.99	$\pm$0.10	mM
$LAC_{(f)}$	0.43	$\pm$0.06	mM
LAC^{max}	18.95	$\pm$0.45	mM
$GLU_{(t0)}$	2.32	$\pm$0.13	mM
$GLU_{(f)}$	4.26	$\pm$0.08	mM
GLU^{max}	4.26	$\pm$0.08	mM

Table S2. Physiological Model Parameters and Std.dev

Par	Value	Std.dev	Units
μ	0.0498	0.0012	h_{-1}
k_d	0.0476	0.0027	h_{-1}
BIO^{max}	41.4873	1.7890	mM
K_{glc}	8.2705	0.1469	mM
K_{gln}	6.8245	2.1693	mM
K_{lac}	18.9461	0.4487	mM
q_{glc}^{exp}	-0.0495	0.0092	$mM/mM*h$
q_{gln}^{exp}	-0.0403	0.0111	$mM/mM*h$
q_{lac}^{exp}	0.0929	0.0030	$mM/mM*h$
q_{glu}^{exp}	0.0057	0.0013	$mM/mM*h$
q_{glc}^{sta}	-0.0252	0.0089	$mM/mM*h$
q_{gln}^{sta}	-0.0022	0.0019	$mM/mM*h$
q_{lac}^{sta}	-0.0714	0.0027	$mM/mM*h$
q_{glu}^{sta}	0.0041	0.0036	$mM/mM*h$

Table S3. Physiological Model Errors

Model	SSE	Pred.Err	MAPE	MPPE
X_v	83.786	3.460	17.885	5.990
GLC	4.624	2.150	5.194	5.497
GLN	0.589	0.768	21.743	7.232
LAC	3.467	1.862	16.424	6.779
GLU	0.183	0.428	3.574	4.806

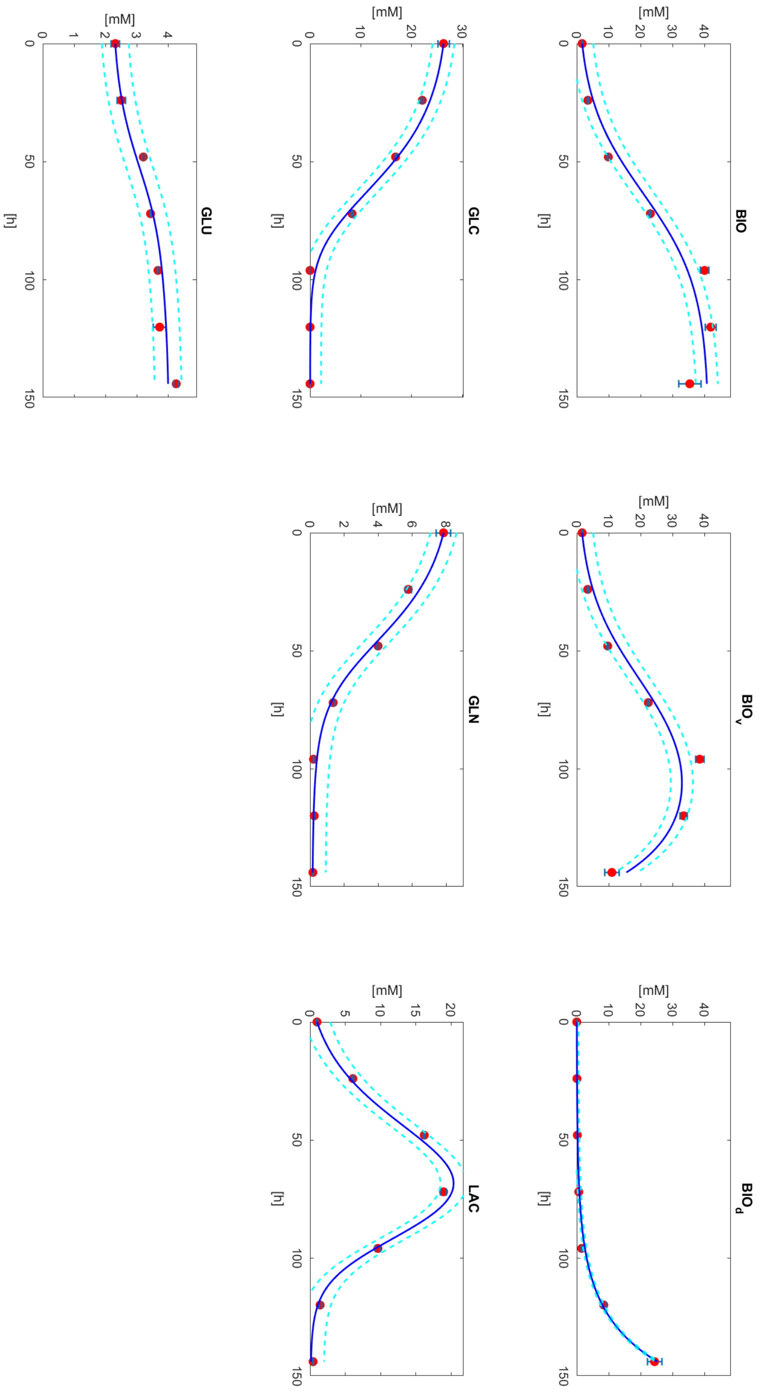

Figure S1. Calculated models with predicted error confidence bands.

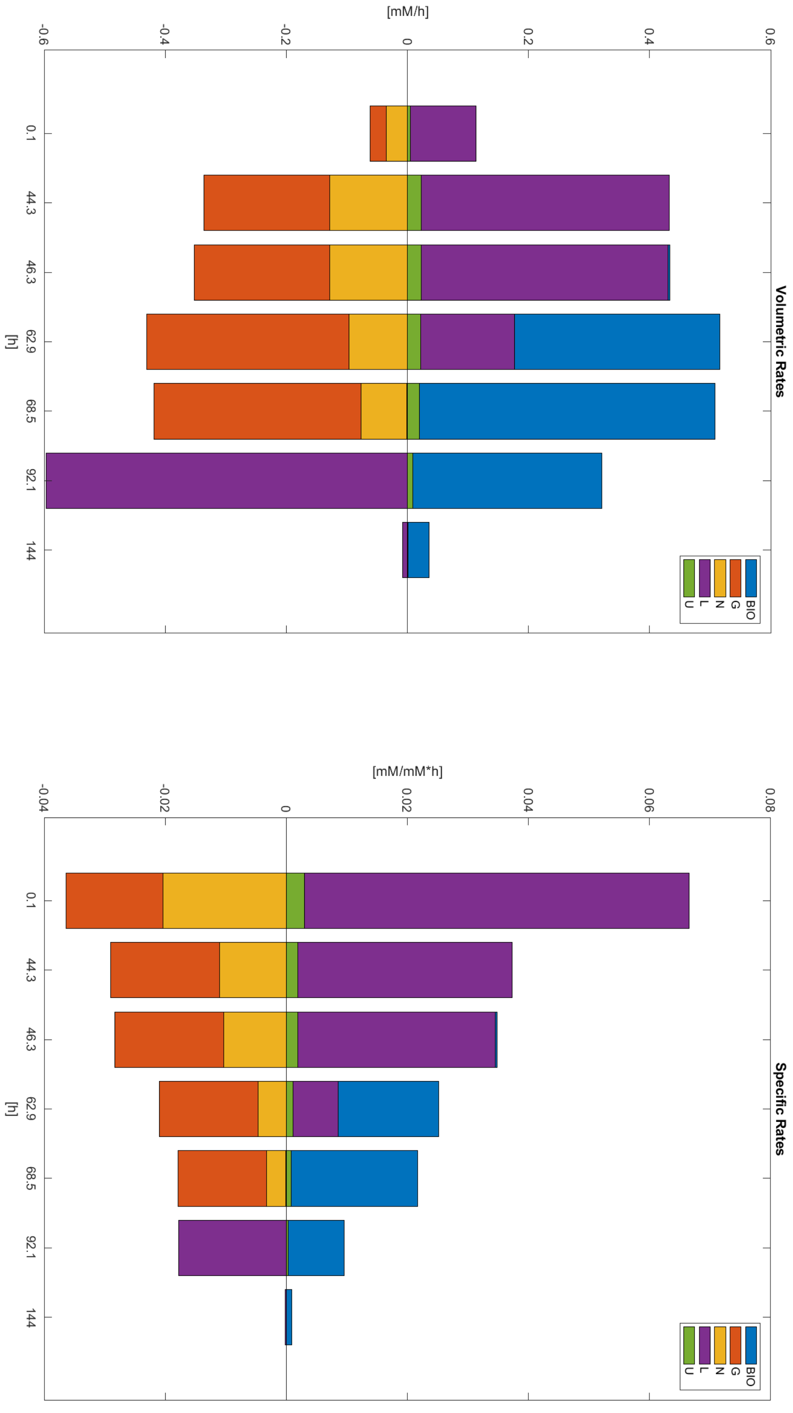

Figure S2. Calculated model effective rates across time.

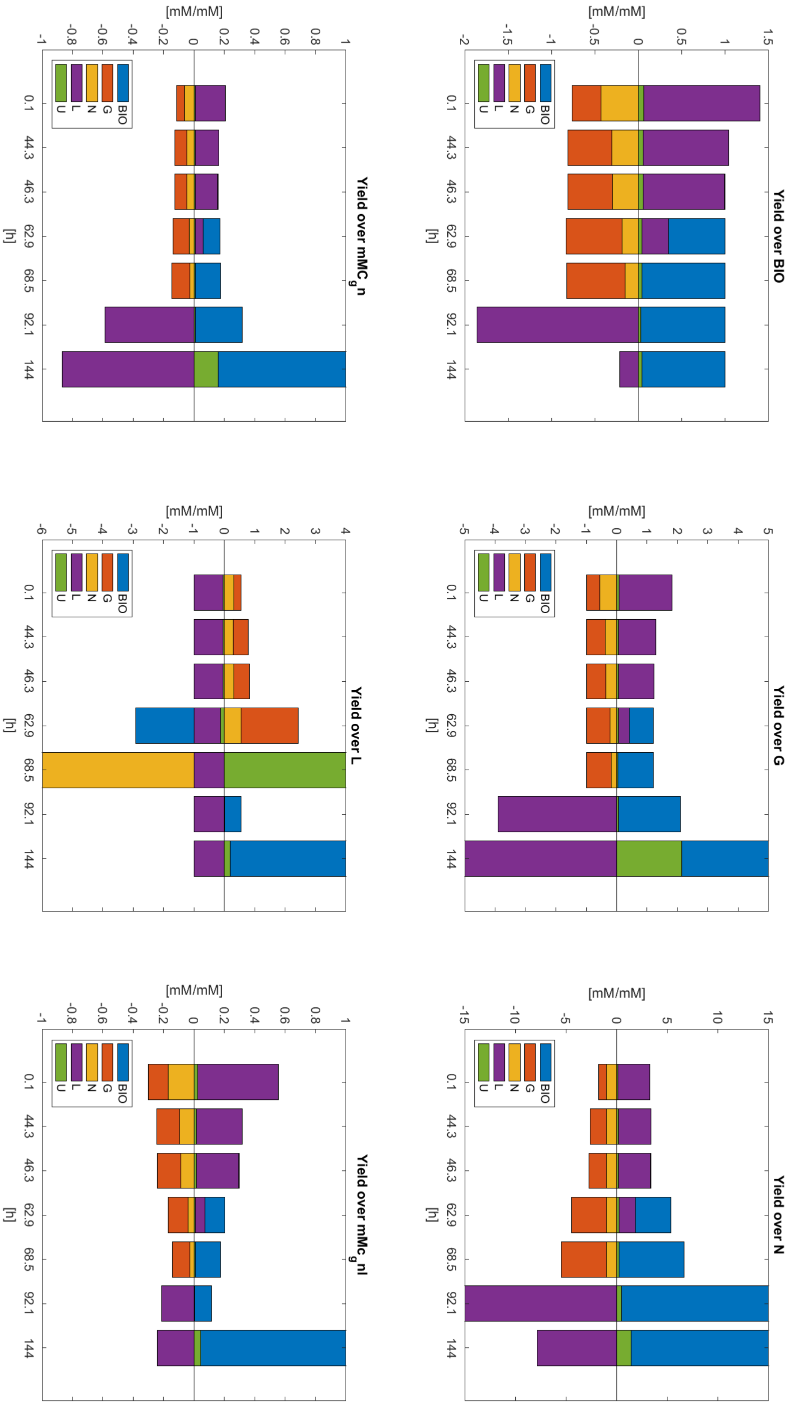

Figure S3. Calculated model effective yields across time.
