# Supplementary material for: Dynamic Modeling of CHO Cell Metabolism Using the Hybrid Cybernetic Approach With a Novel Elementary Mode Analysis Strategy
Source: Front Bioeng Biotechnol. 2020 Apr 15;8:279. doi: 10.3389/fbioe.2020.00279 (PMC7174696; doi:10.3389/fbioe.2020.00279)
Supplement: Supplementary file 3 [file Data_Sheet_3.PDF]

Supplementary material 3: Metabolic Model data, parameters and error values

1 SUPPLEMENTARY TABLES AND FIGURES

Table S1. Metabolic models kinetic parameters

Parameter	YSA	LYSA	PSYA	LPSYA
k_1	2.56E-11	0.2002	0.0613	0.202
k_2	0.1584	0.0335	0.0014	0.247
k_3	1.6793	—	8.140E-09	—
k_4	—	—	0.1221	—
k_5	—	—	0.4003	—
k_6	—	—	0.0054	—
k_7	—	—	0.0016	—
k_8	—	—	0.0022	—
K_1	0.0067	53.2704	6.6130	48.726
K_2	31.9663	3.7068	218.1463	138.611
K_3	1498.4422	—	70.1337	—
K_4	—	—	26.7886	—
K_5	—	—	89.1361	—
K_6	—	—	171.3228	—
K_7	—	—	141.4275	—
K_8	—	—	97.4006	—

Table S2. Metabolic models error parameters

	MSSE	MAPE	PE	MPPE
YSA	11.83	66.03	14.20	7.46
LYSA	15.79	88.13	18.95	9.96
PSYA	2.19	12.23	2.63	1.38
LPSYA	6.97	38.90	8.36	4.39

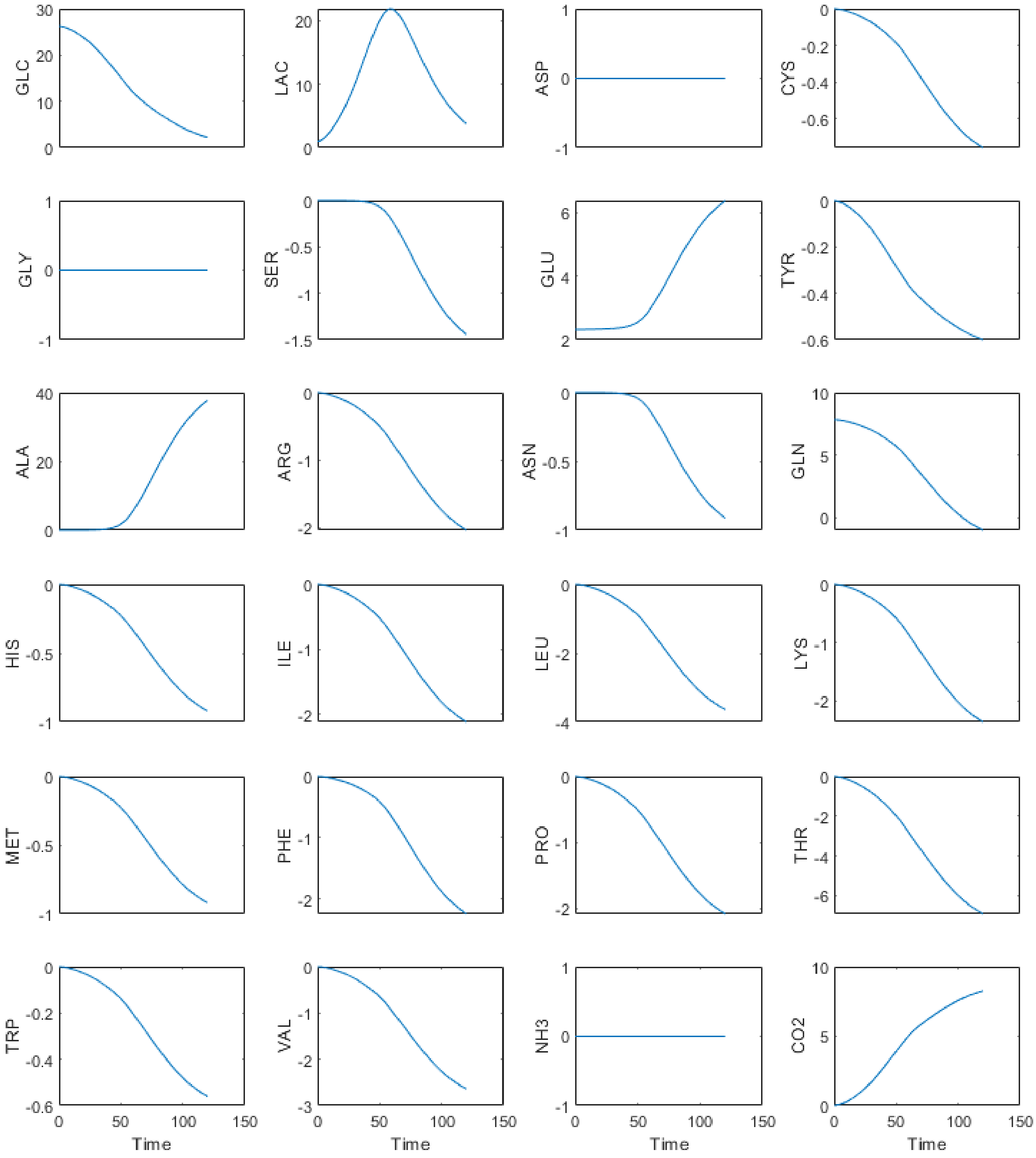

Figure S1. Calculated YSA-HCM model extracellular metabolites in mM, non measured metabolites present 0 initial value and therefore are understood as ΔM.

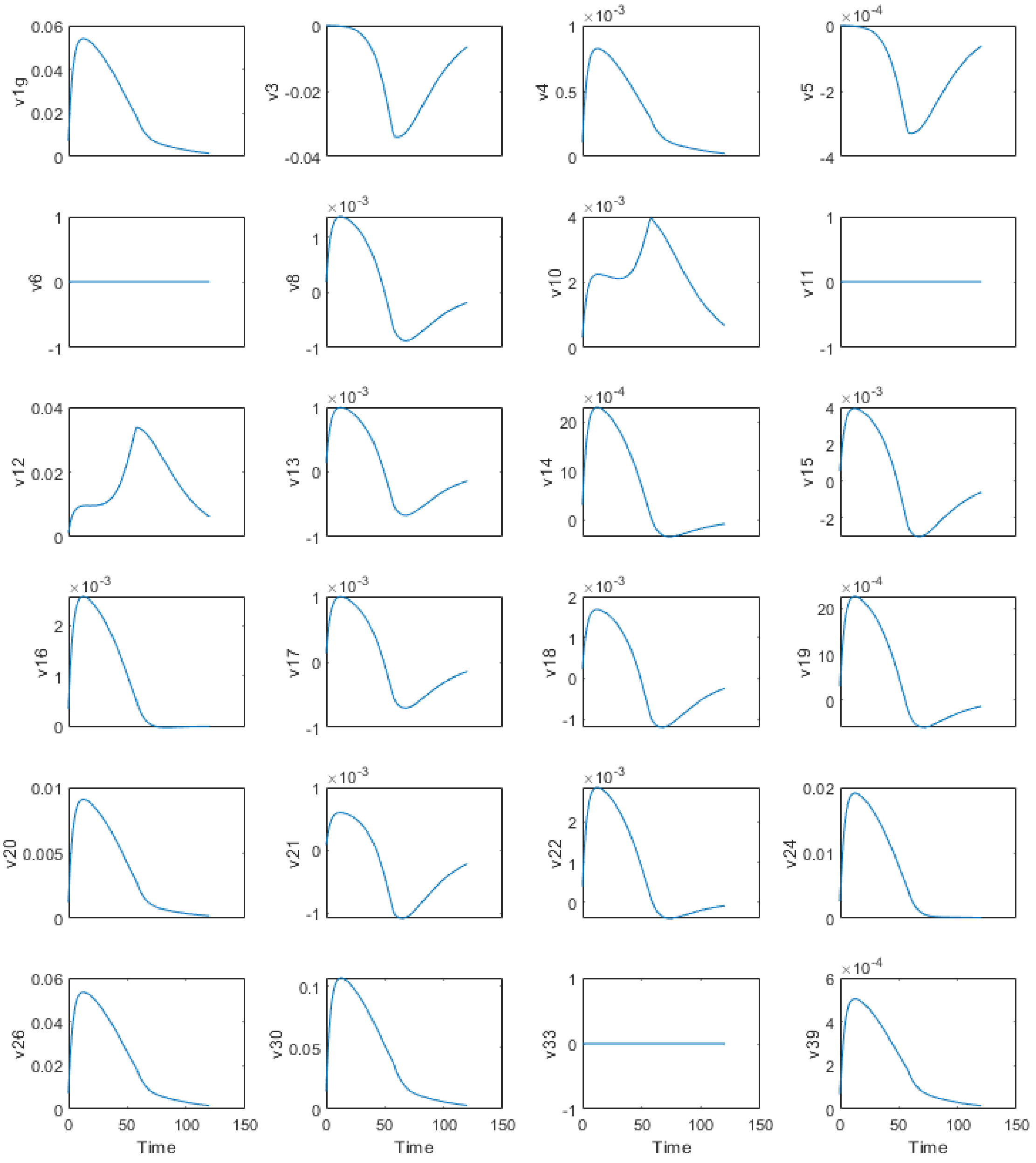

Figure S2. Calculated YSA-HCM model fluxes in mM/h pt1.

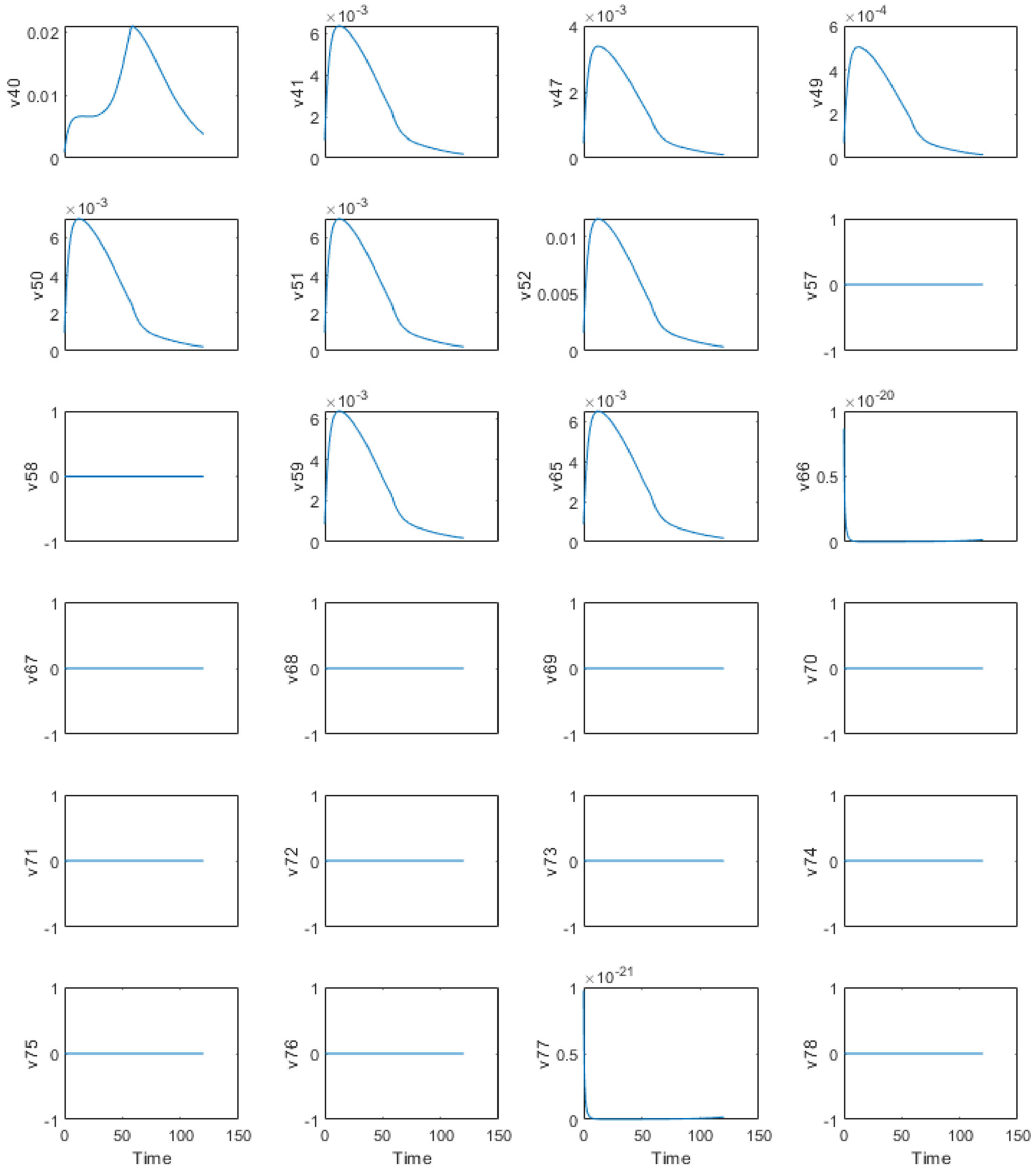

Figure S3. Calculated YSA-HCM model fluxes in mM/h pt2.

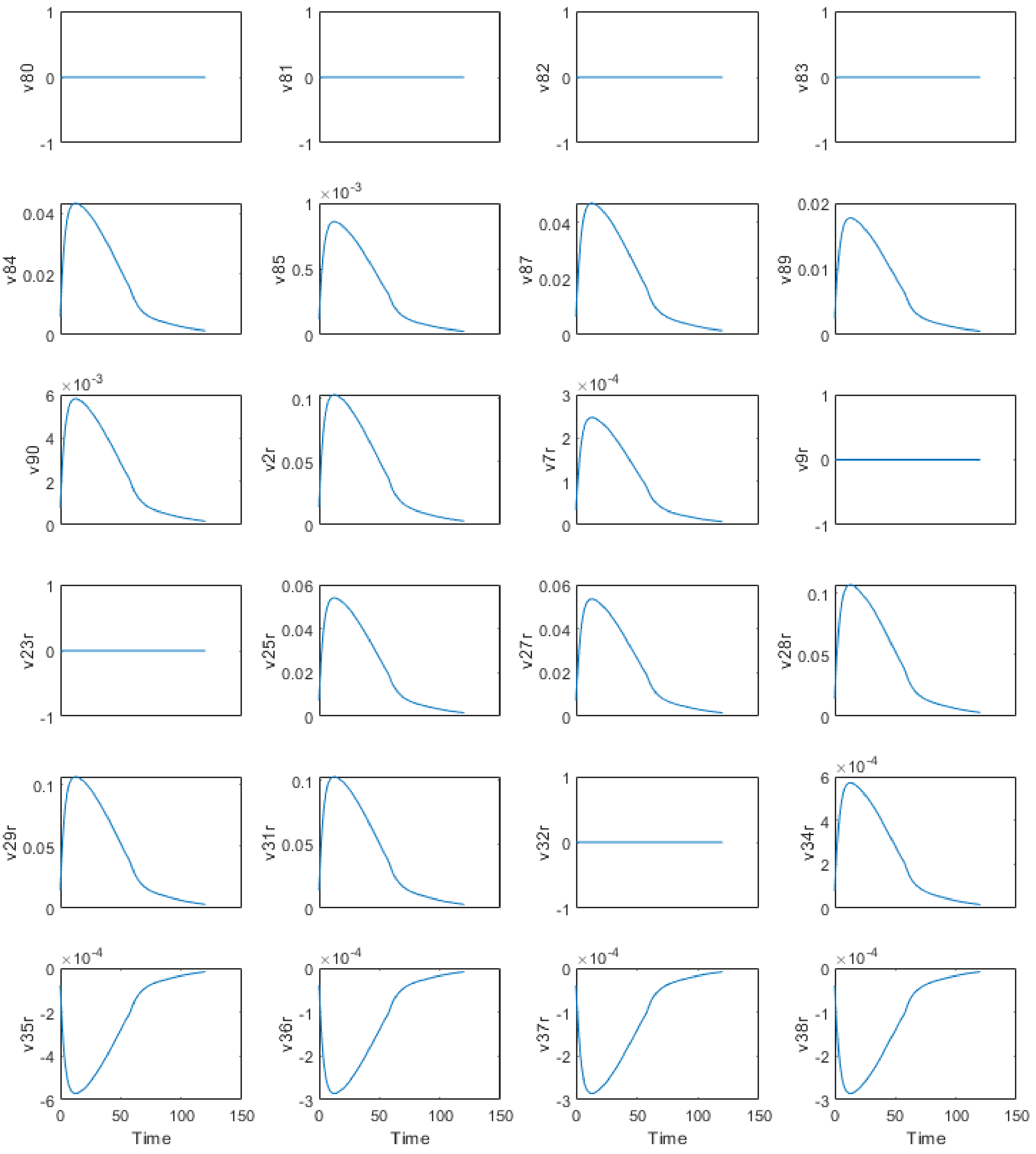

Figure S4. Calculated YSA-HCM model fluxes in mM/h pt3.

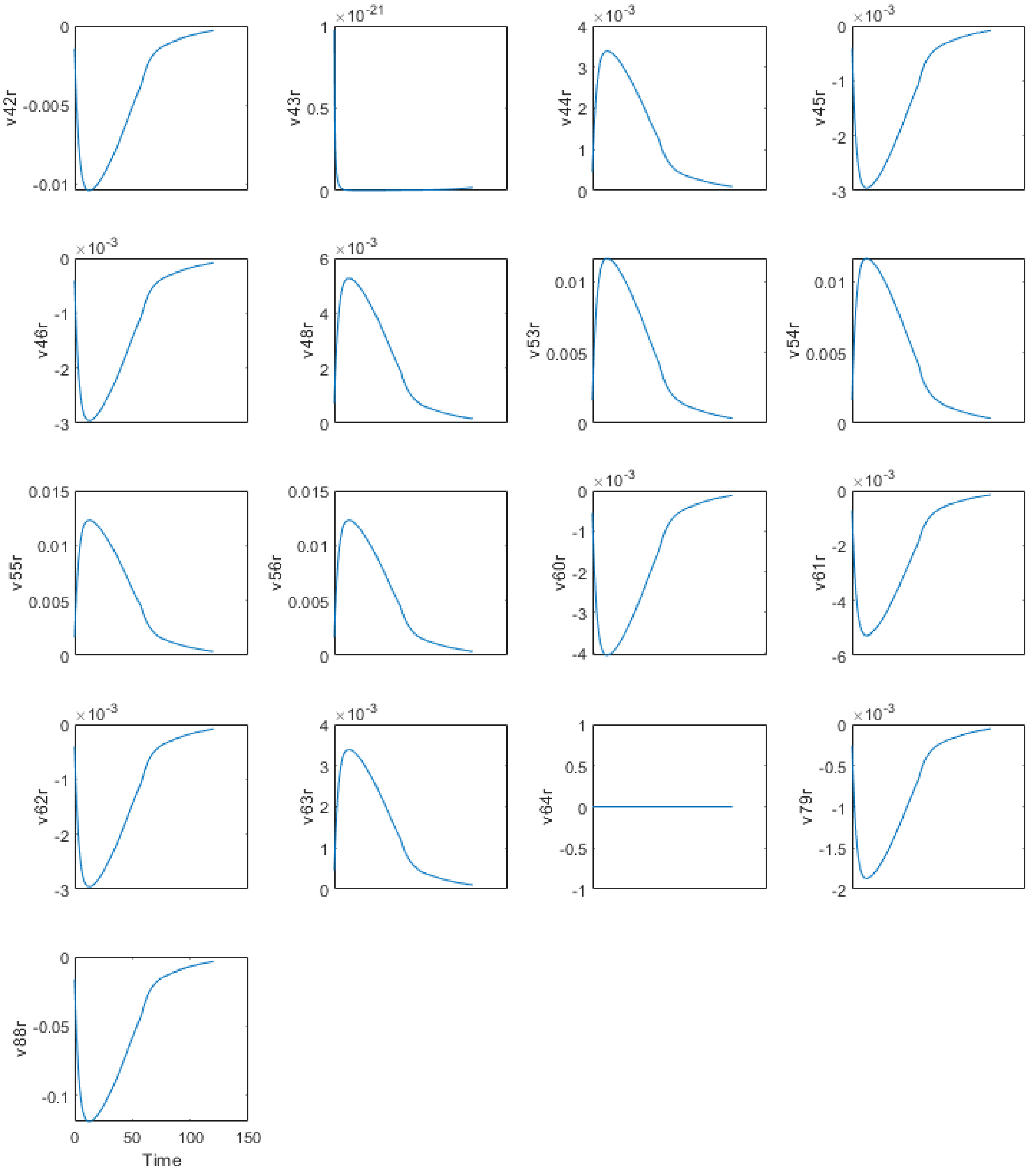

Figure S5. Calculated YSA-HCM model fluxes in mM/h pt4.

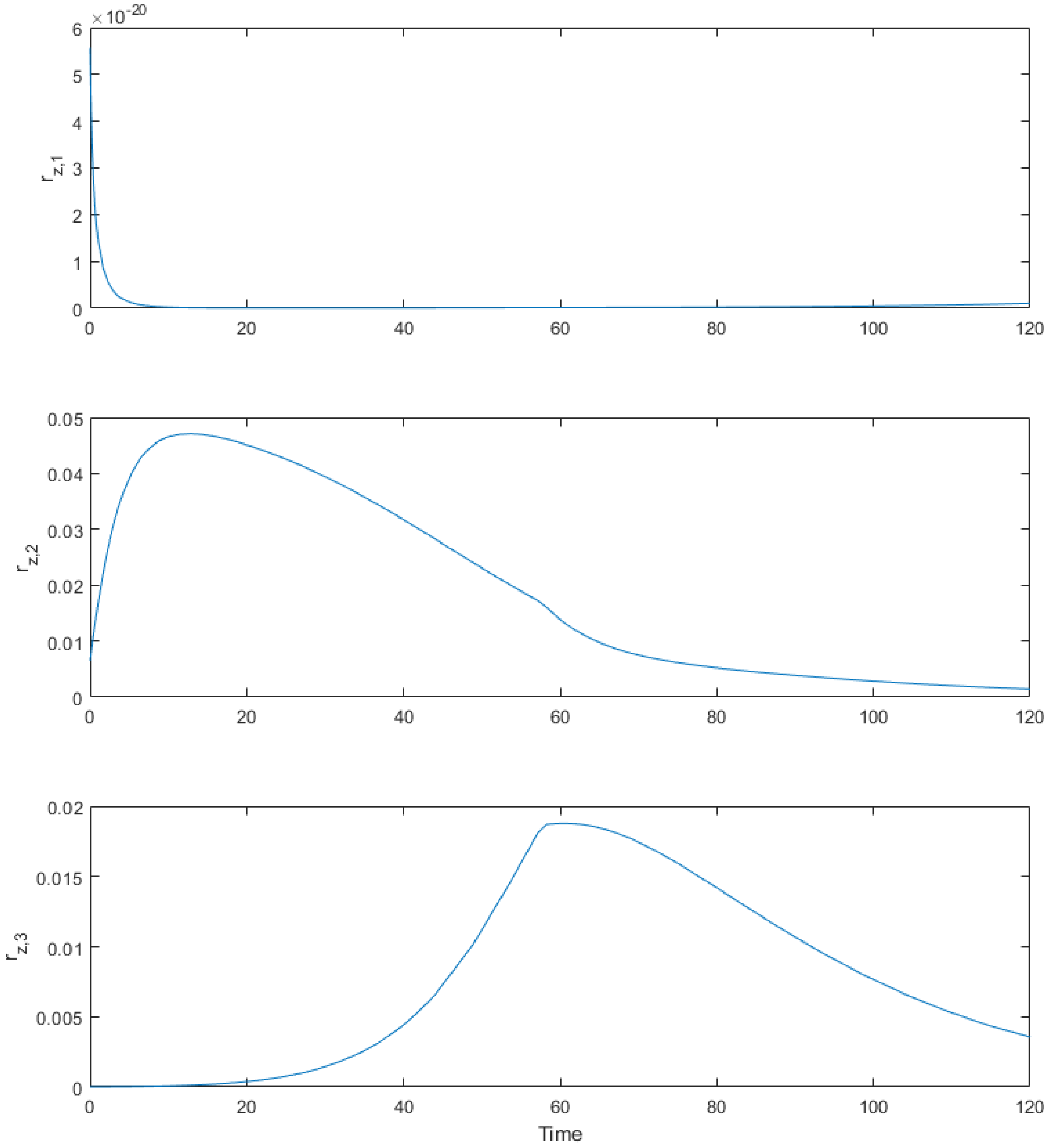

Figure S6. Calculated YSA-HCM model rates across active EMs in mM/h.

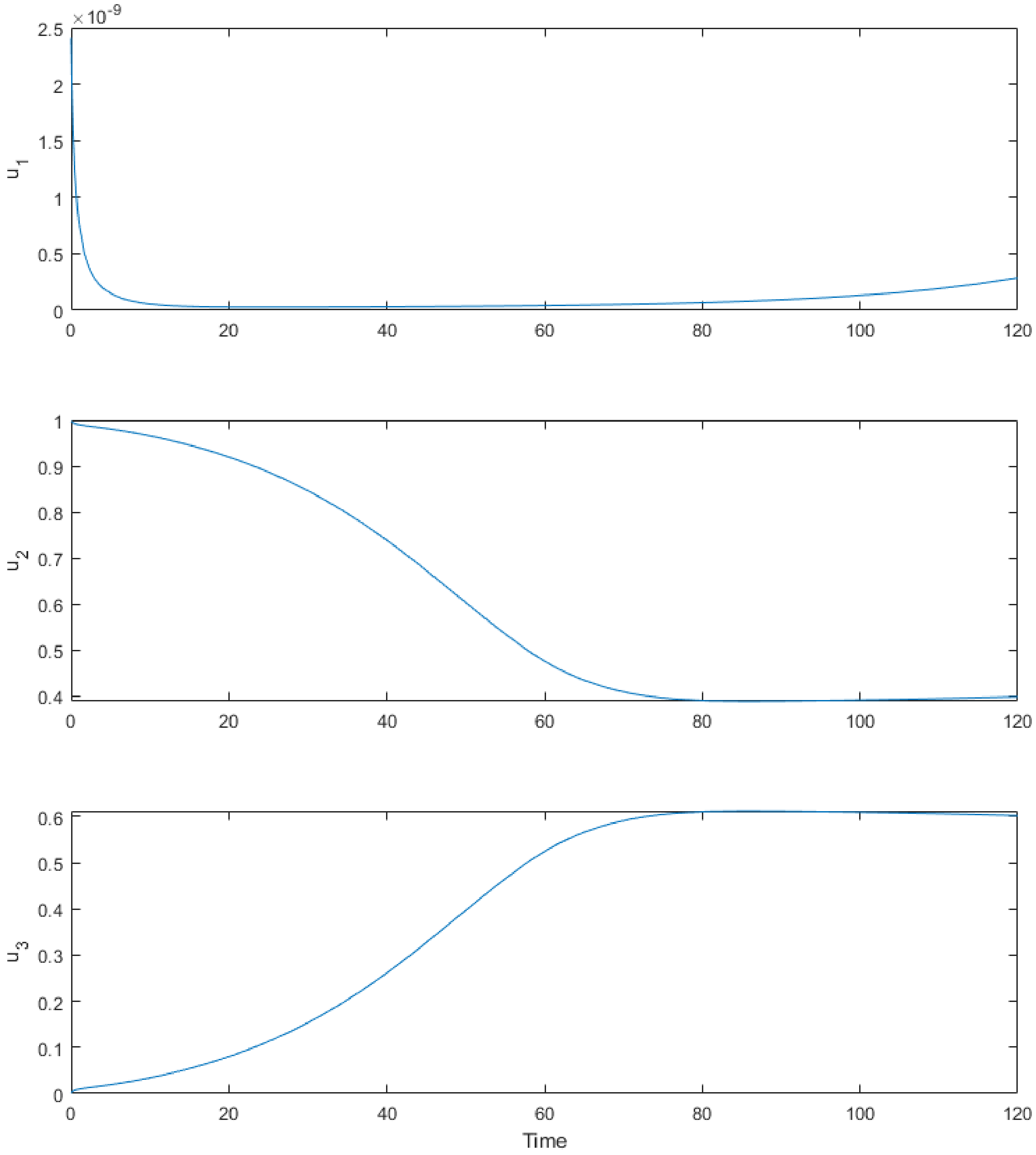

Figure S7. Calculated YSA-HCM model calculated cybernetic variable u.

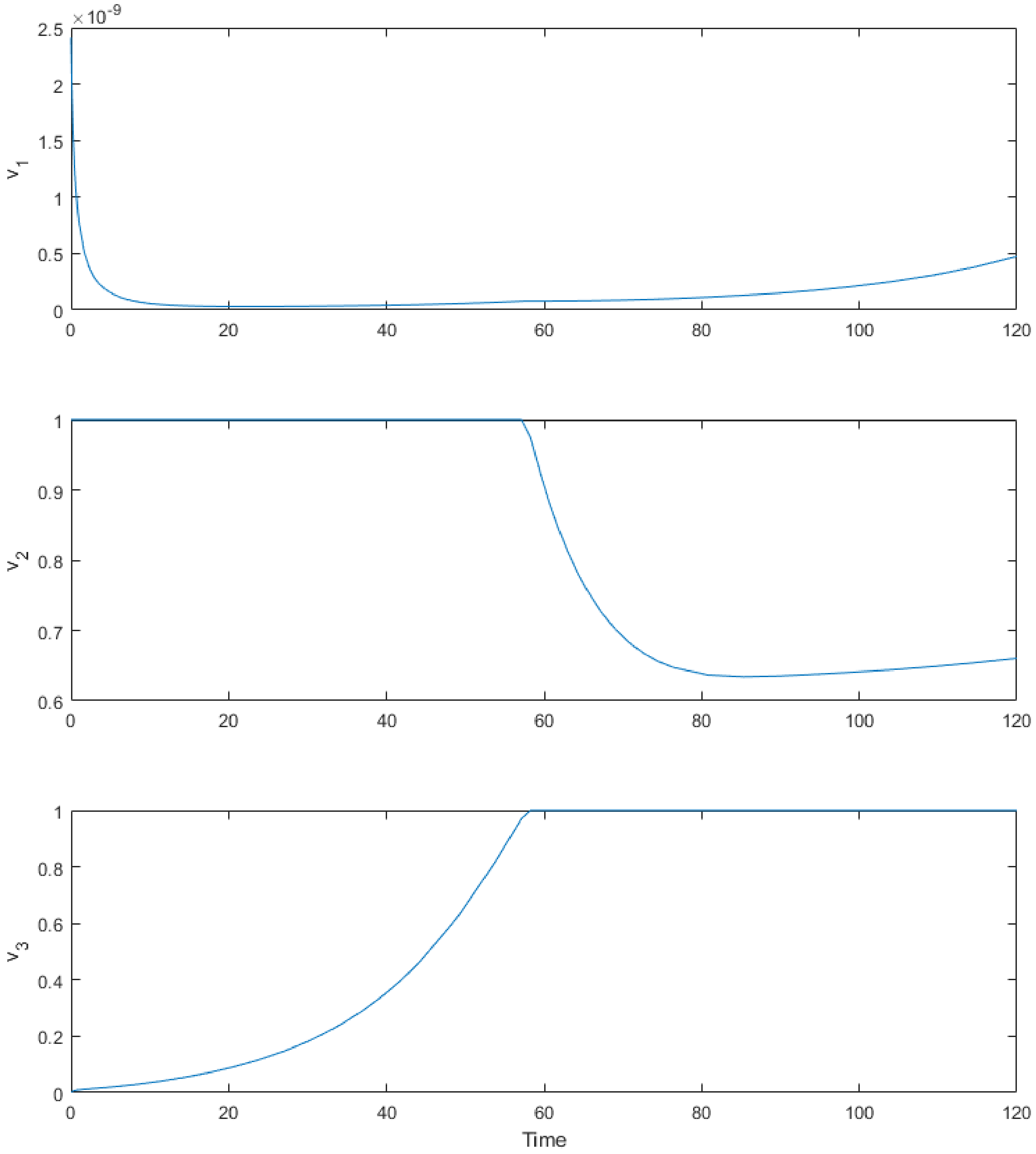

Figure S8. Calculated YSA-HCM model calculated cybernetic variable v.

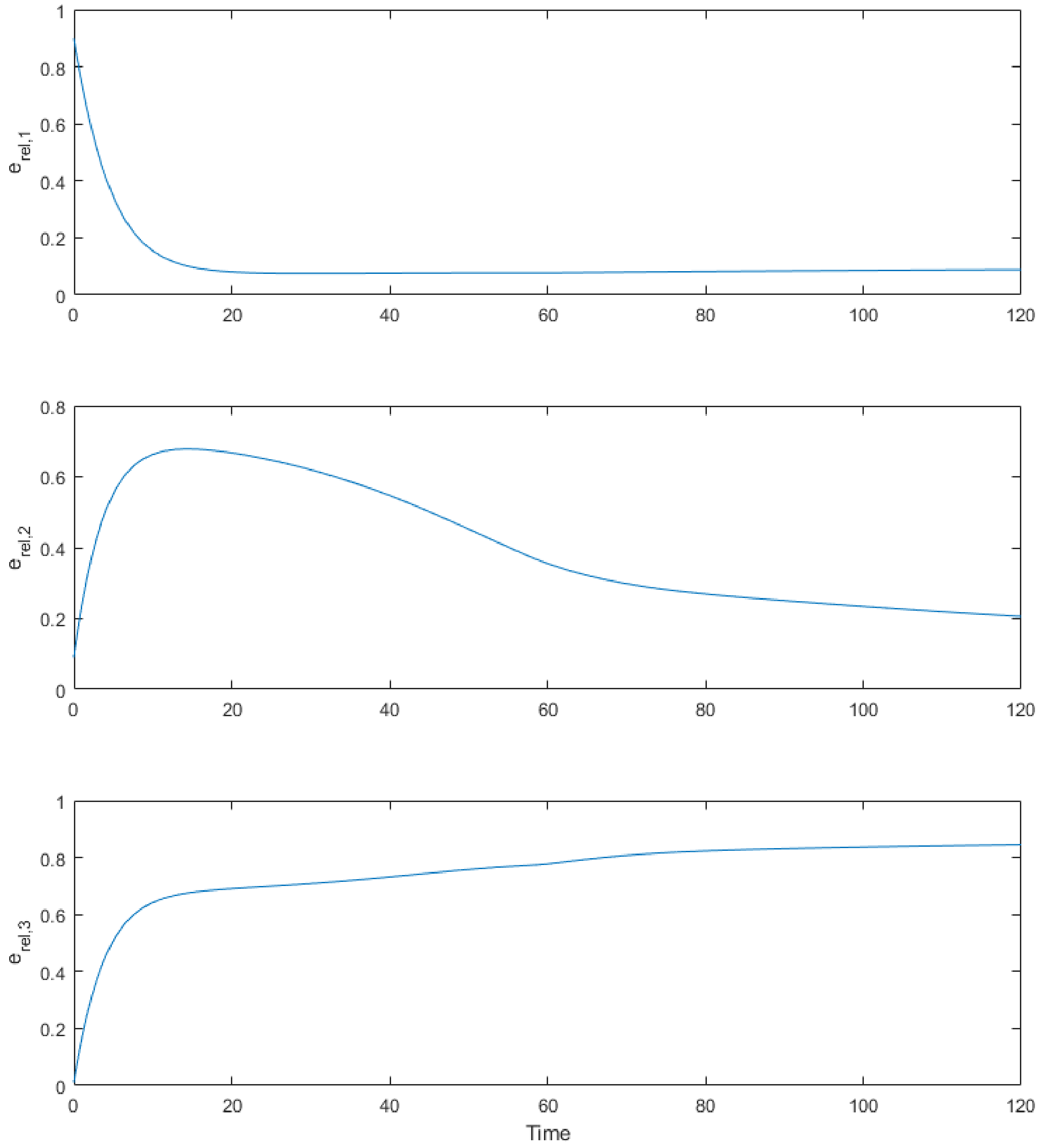

Figure S9. Calculated YSA-HCM model calculated relative enzyme.

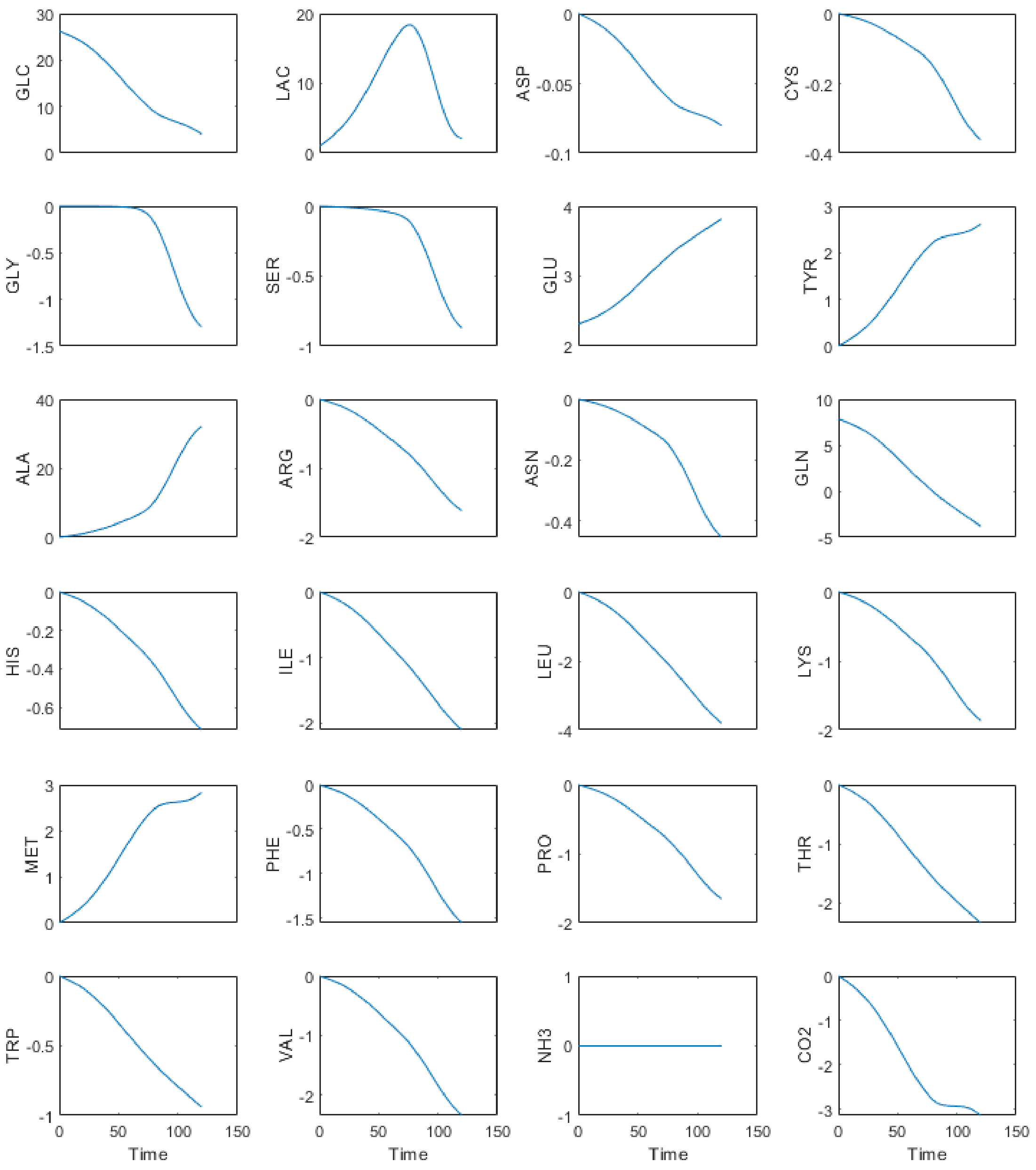

Figure S10. Calculated LYSA-HCM model extracellular metabolites in mM, non measured metabolites present 0 initial value and therefore are understood as ΔM.

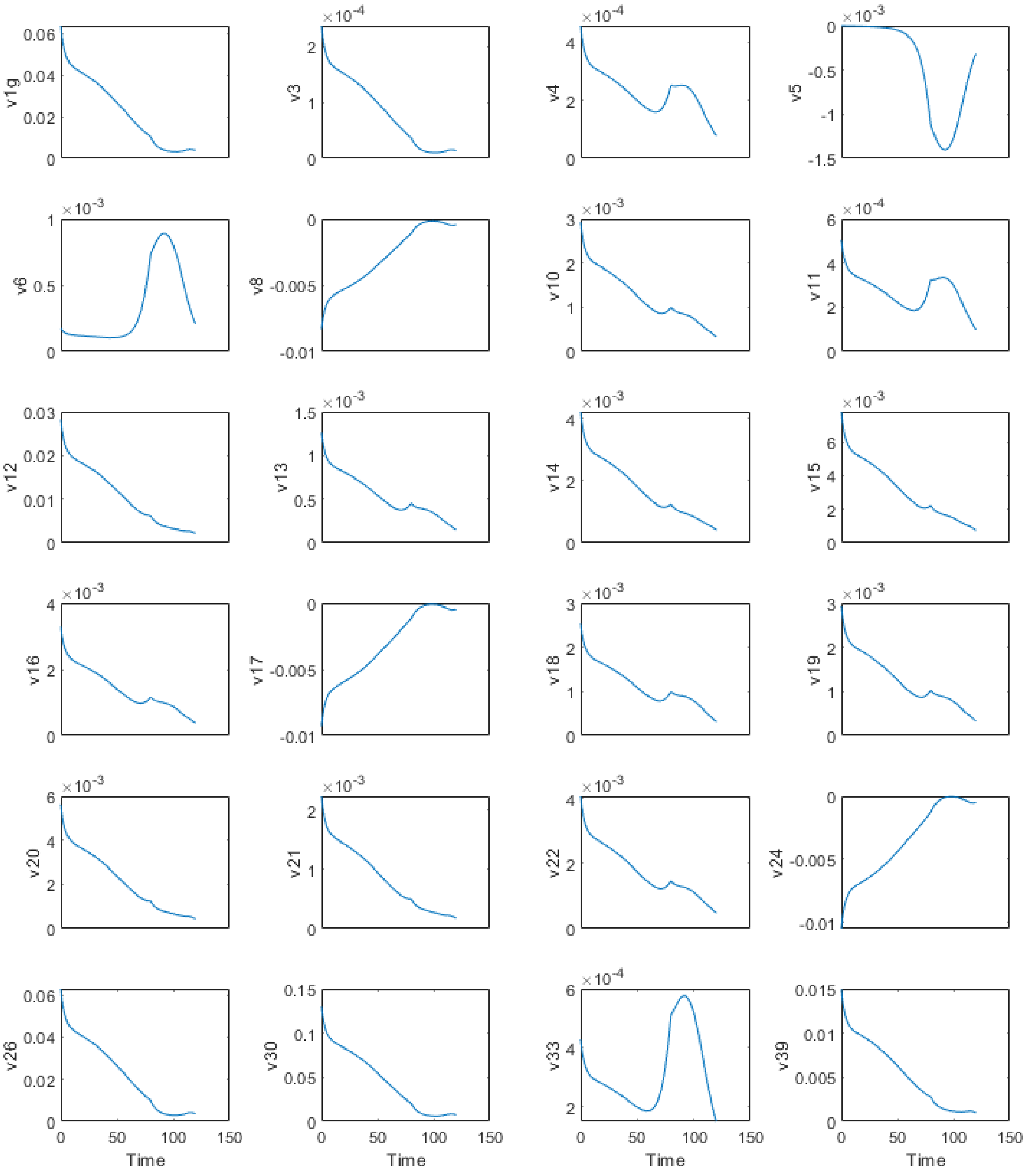

Figure S11. Calculated LYSA-HCM model fluxes in mM/h pt1.

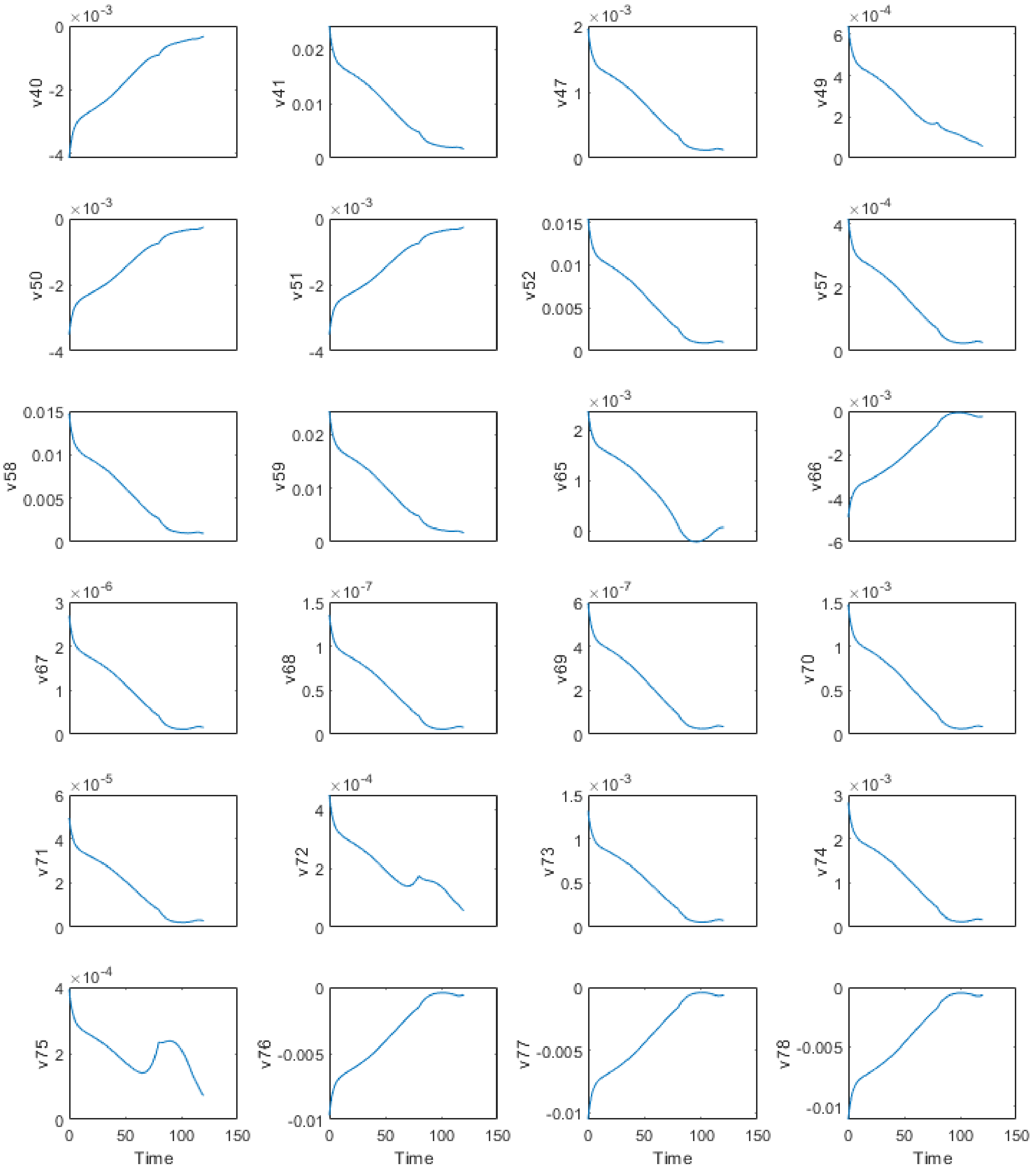

Figure S12. Calculated LYSA-HCM model fluxes in mM/h pt2.

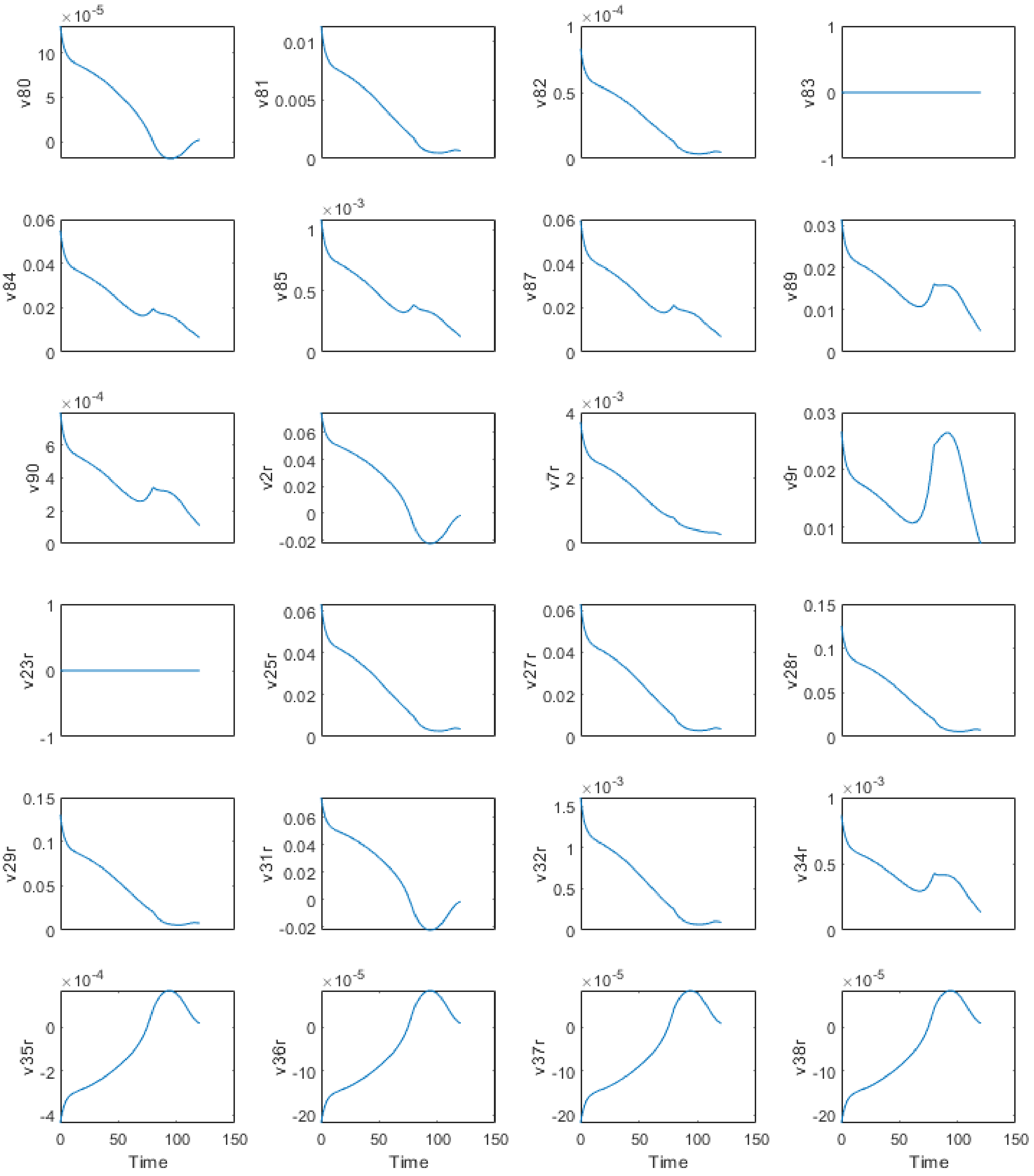

Figure S13. Calculated LYSA-HCM model fluxes in mM/h pt3.

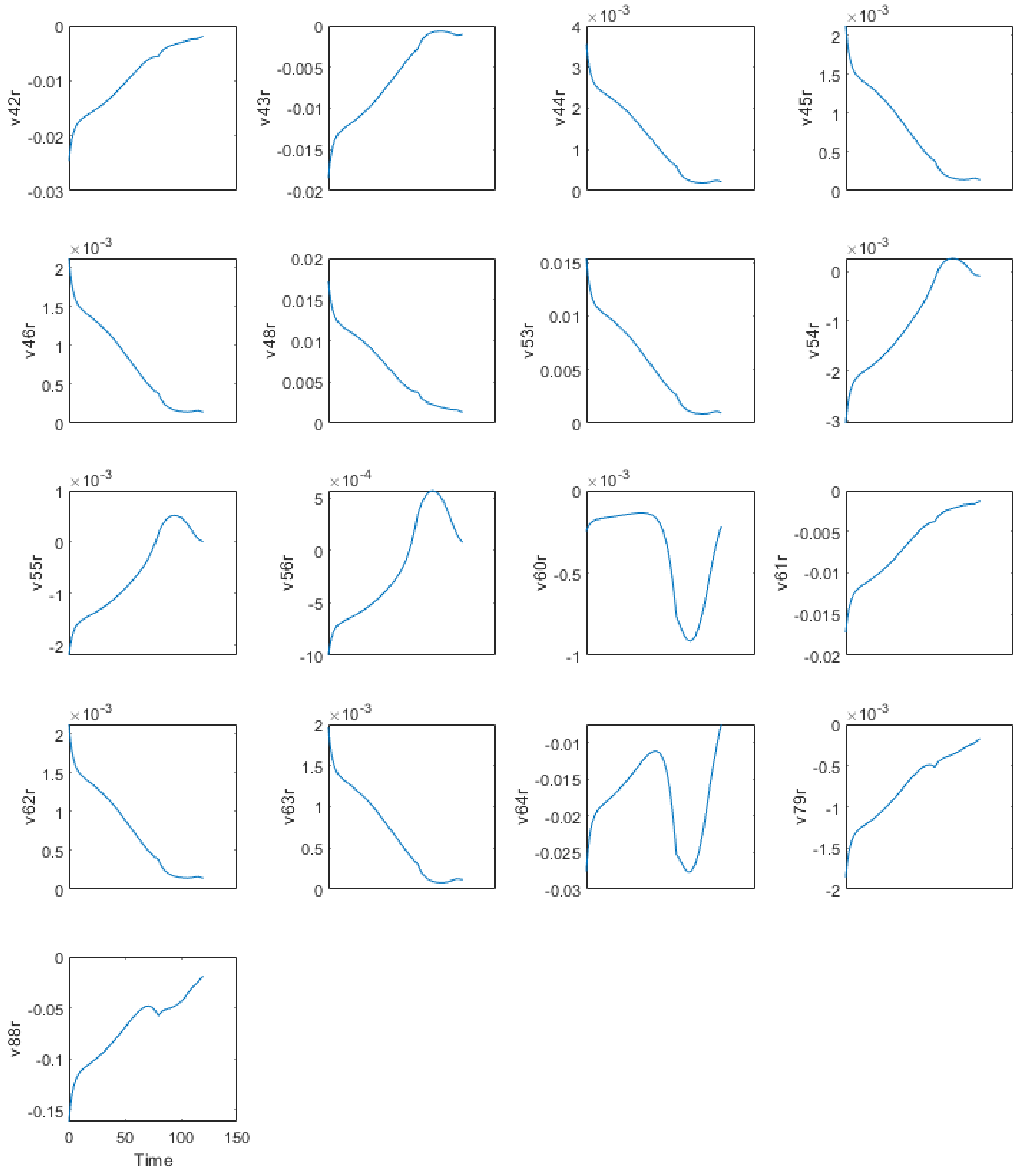

Figure S14. Calculated LYSA-HCM model fluxes in mM/h pt4.

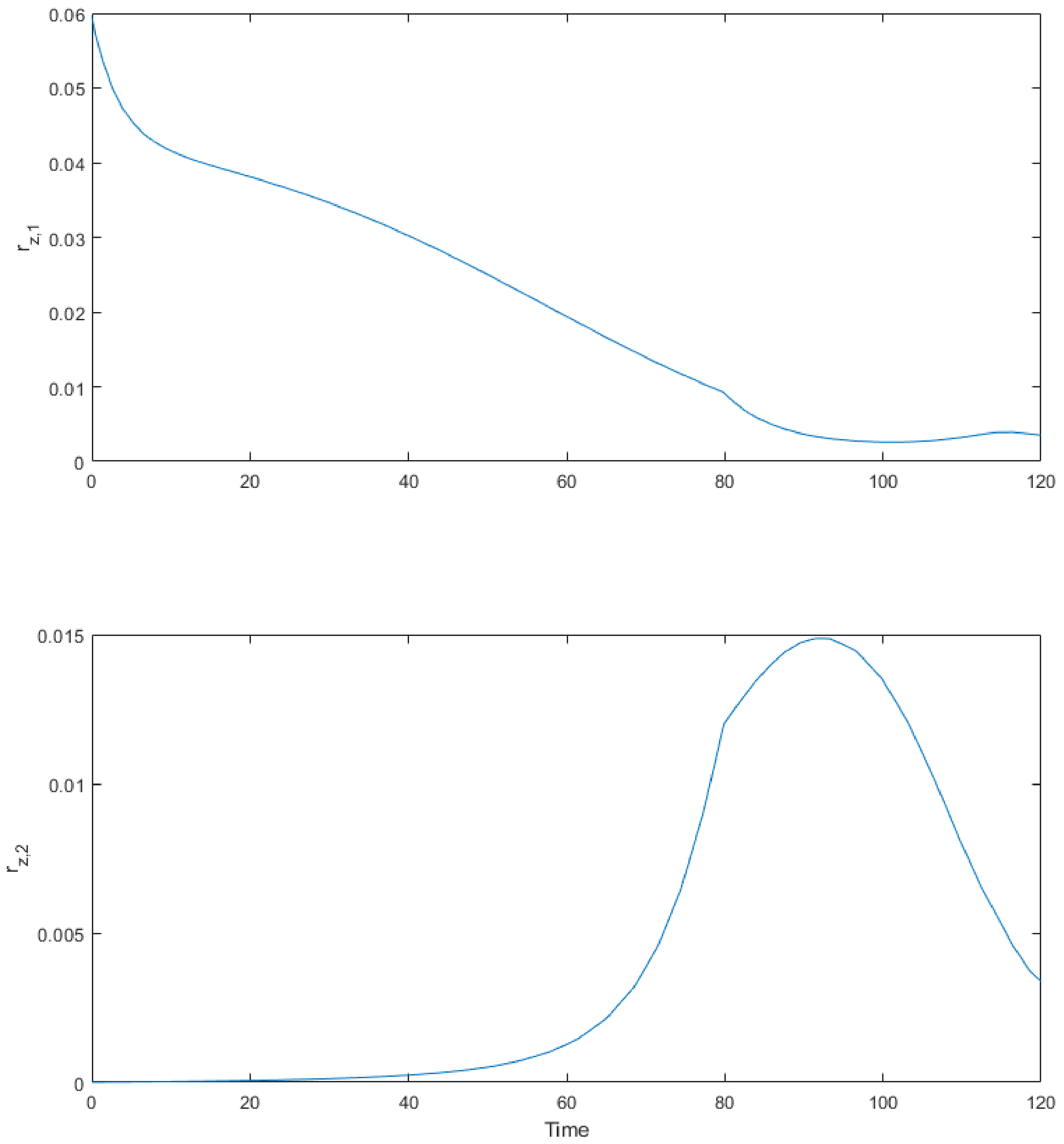

Figure S15. Calculated LYSA-HCM model rates across active EMs in mM/h.

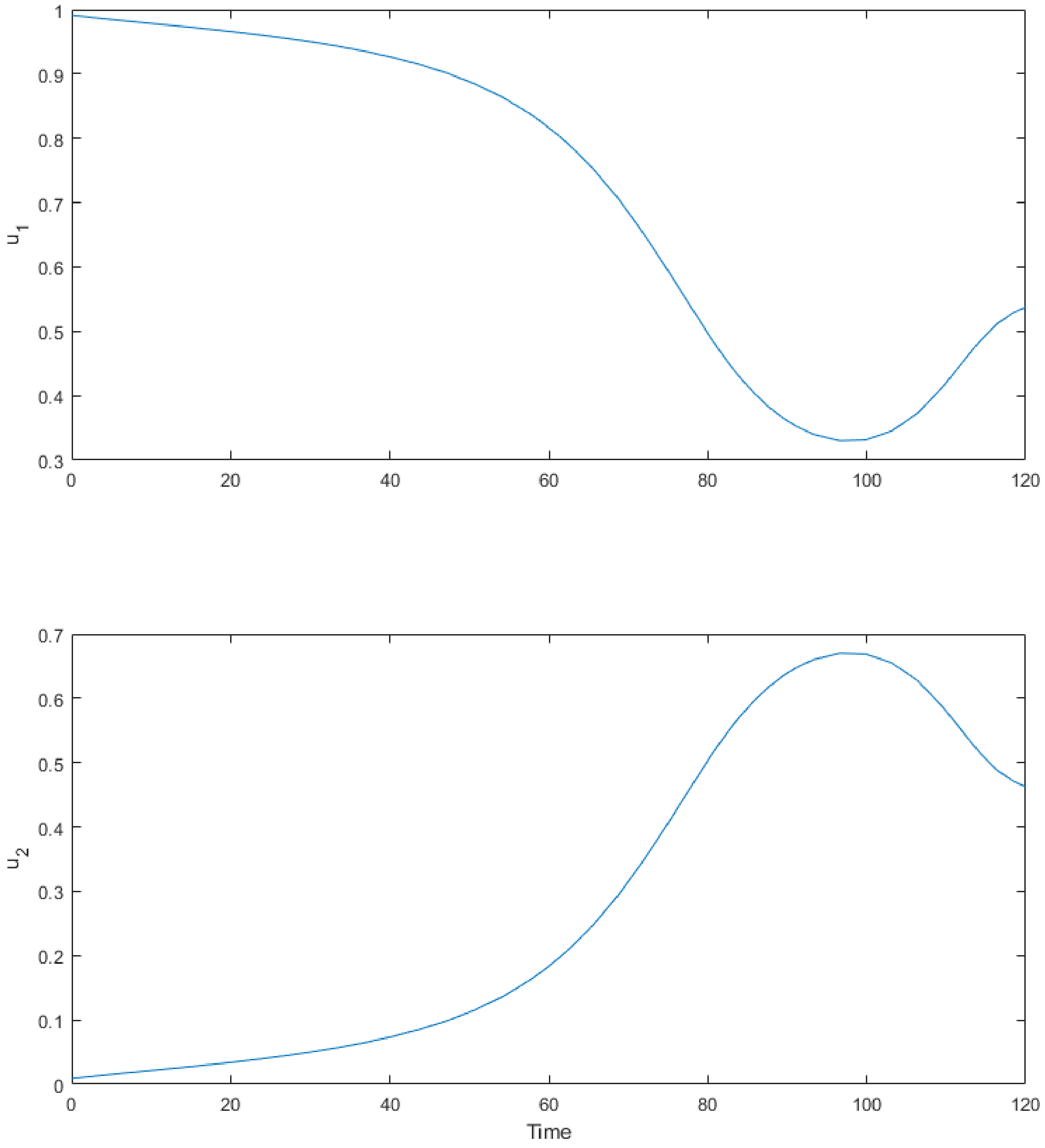

Figure S16. Calculated LYSA-HCM model calculated cybernetic variable u.

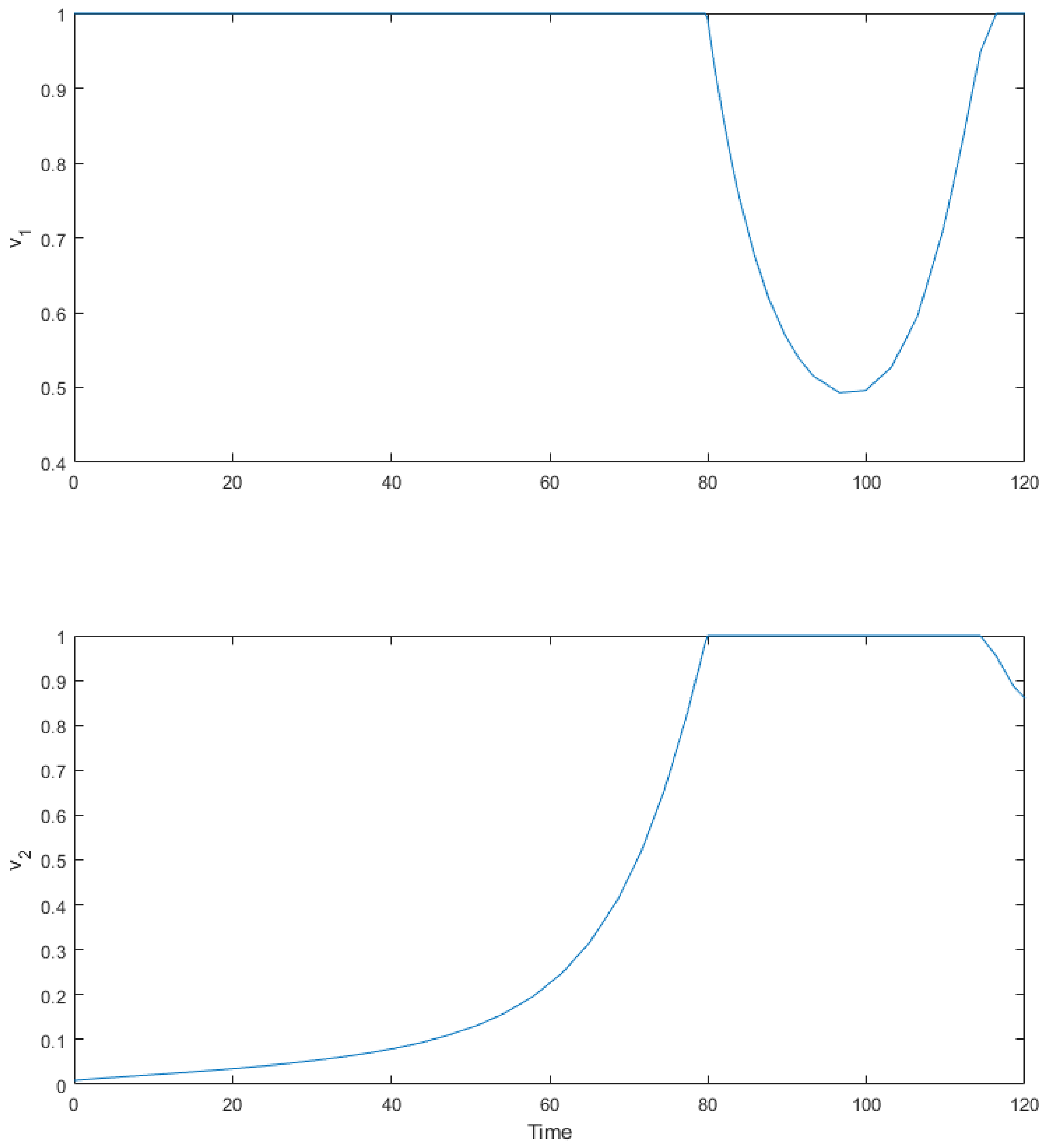

Figure S17. Calculated LYSA-HCM model calculated cybernetic variable v.

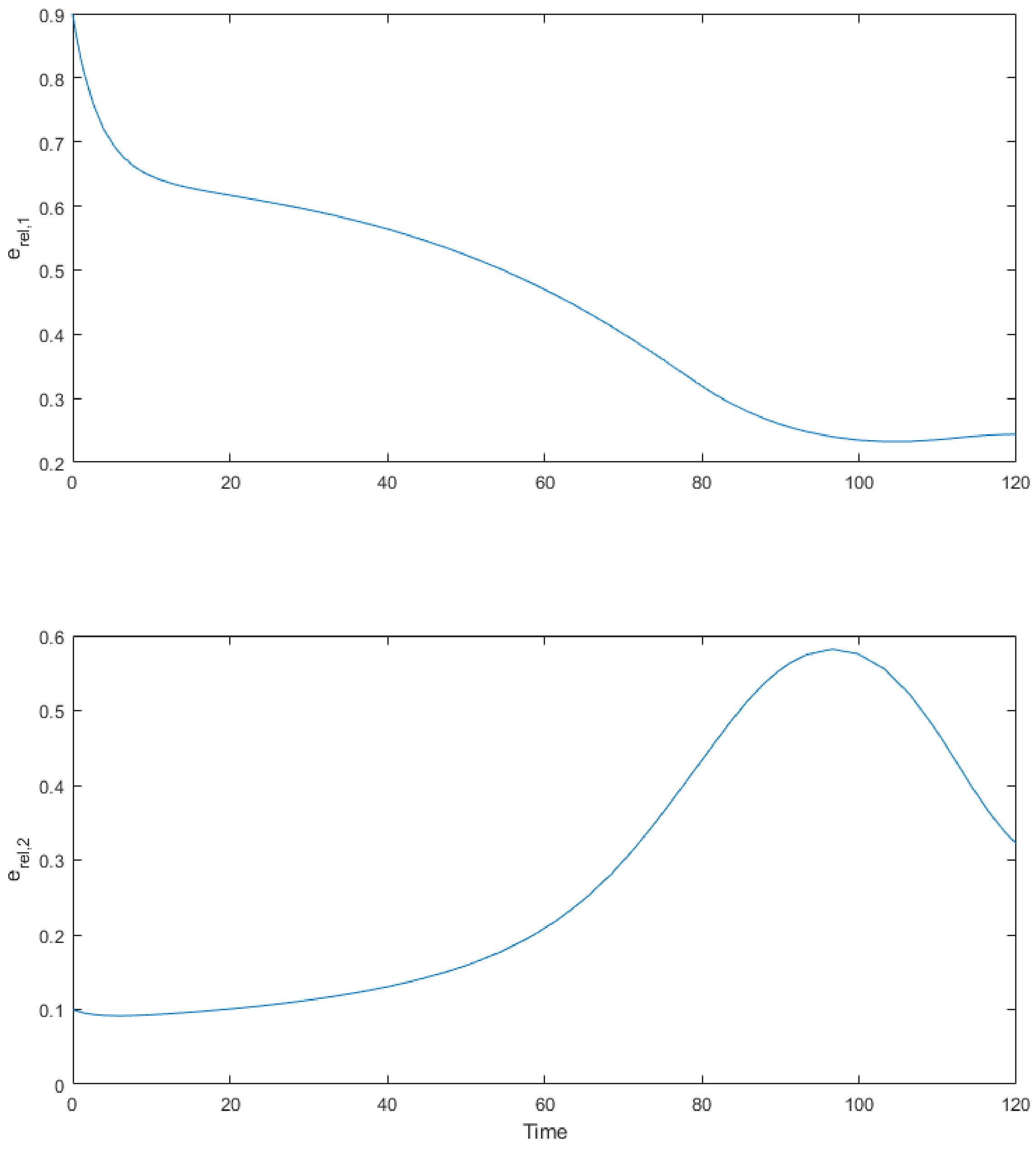

Figure S18. Calculated LYSA-HCM model calculated relative enzyme.

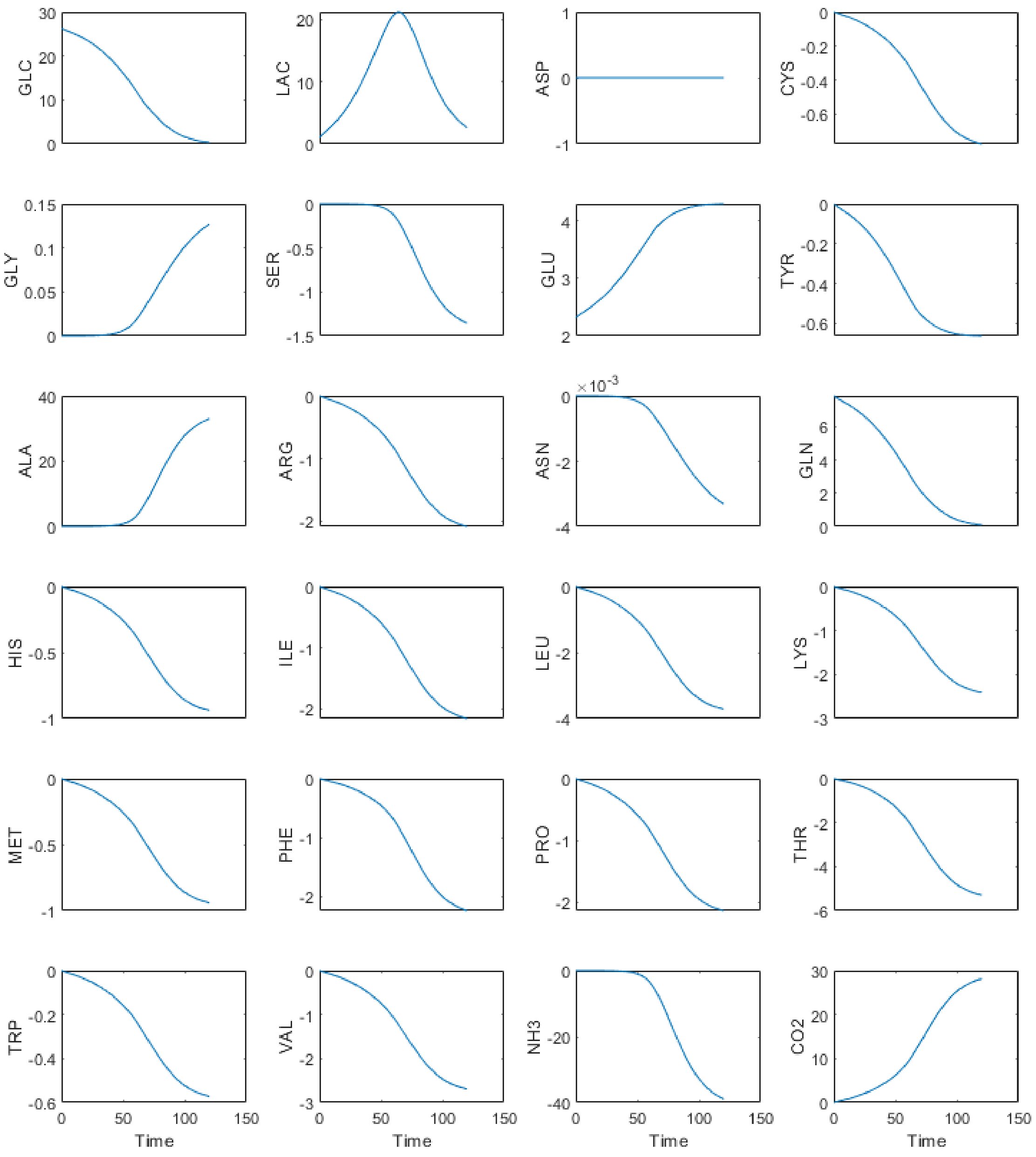

Figure S19. Calculated PSYA-HCM model extracellular metabolites in mM, non measured metabolites present 0 initial value and therefore are understood as ΔM.

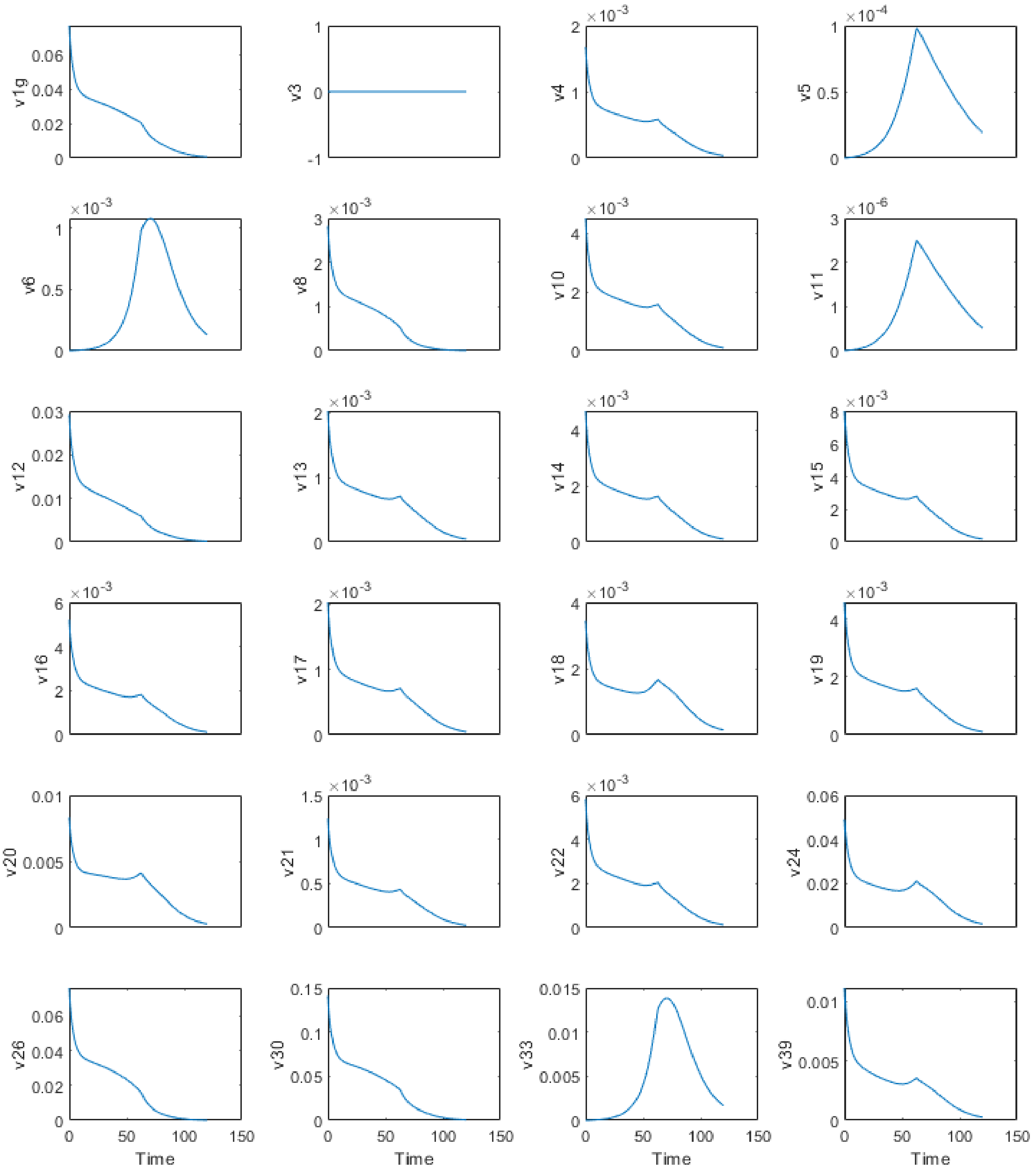

Figure S20. Calculated PSYA-HCM model fluxes in mM/h pt1.

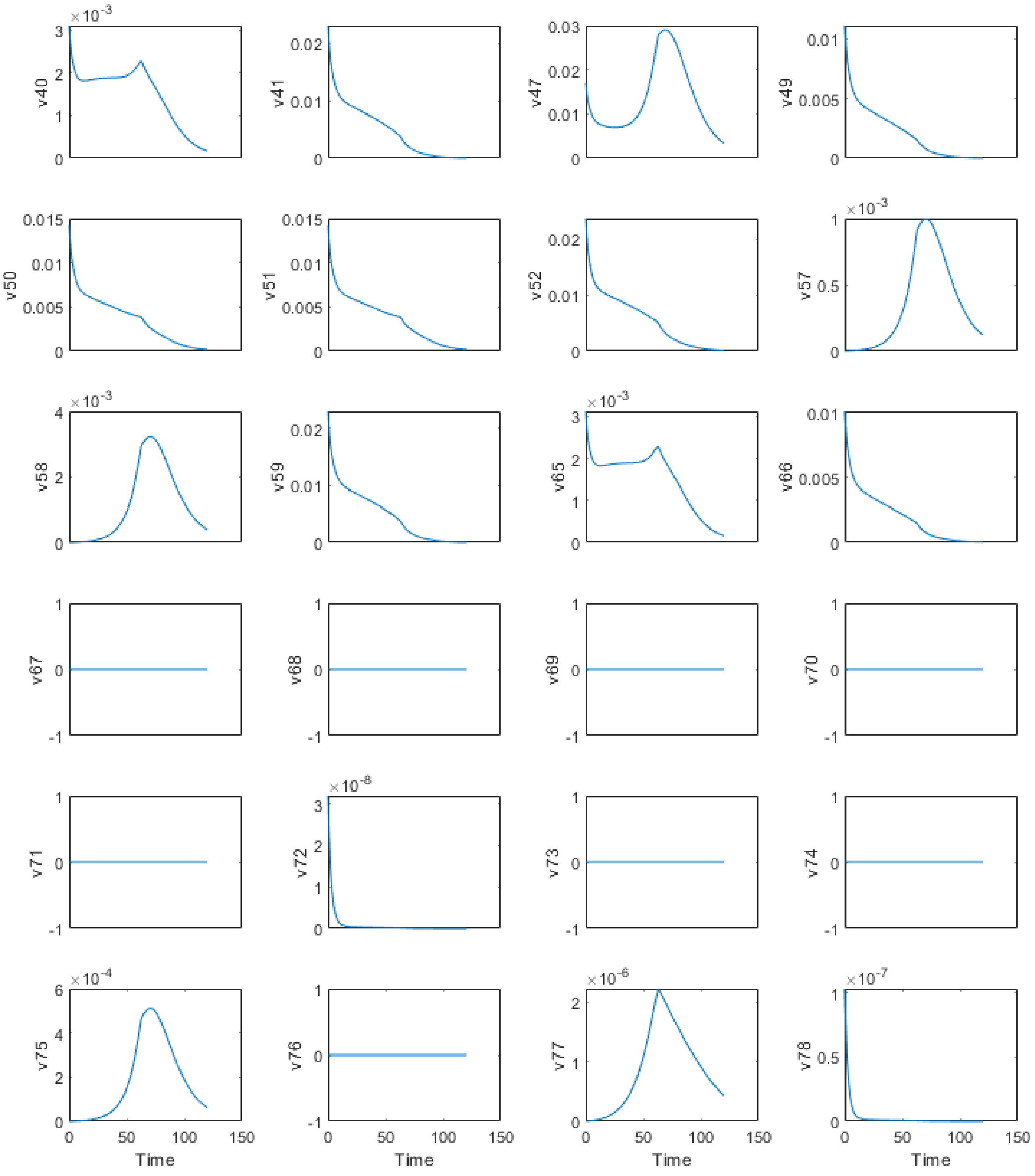

Figure S21. Calculated PSYA-HCM model fluxes in mM/h pt2.

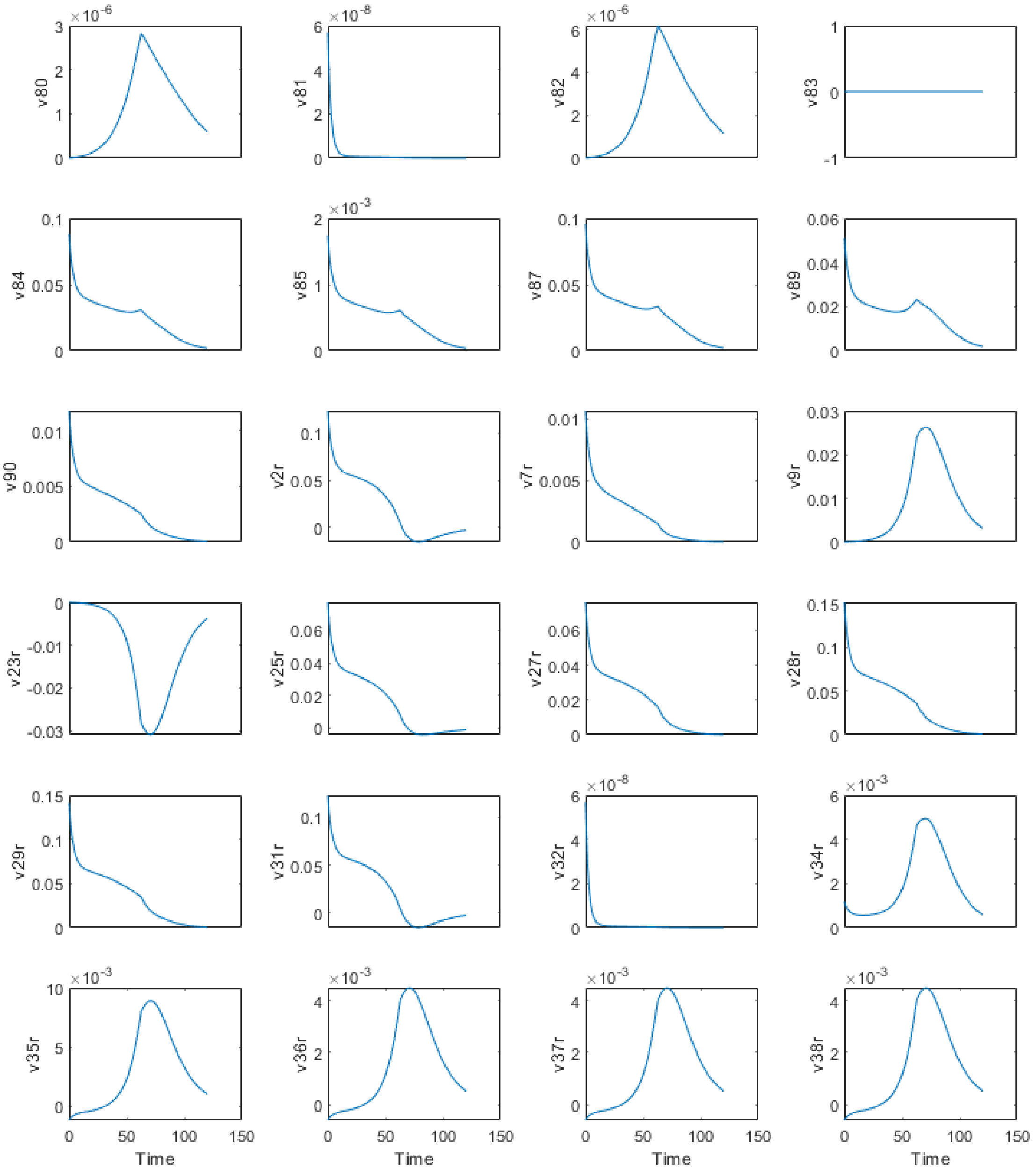

Figure S22. Calculated PSYA-HCM model fluxes in mM/h pt3.

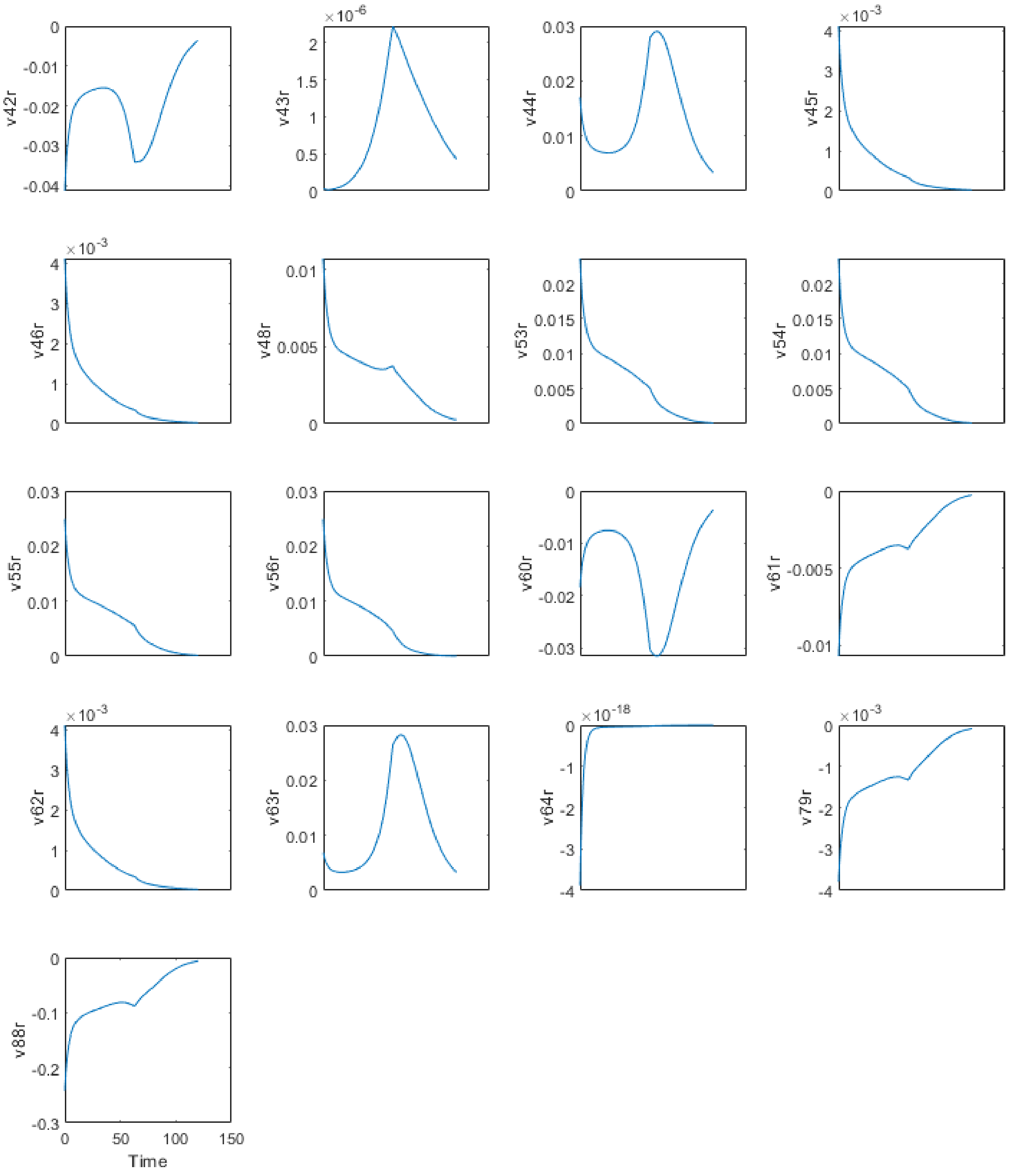

Figure S23. Calculated PSYA-HCM model fluxes in mM/h pt4.

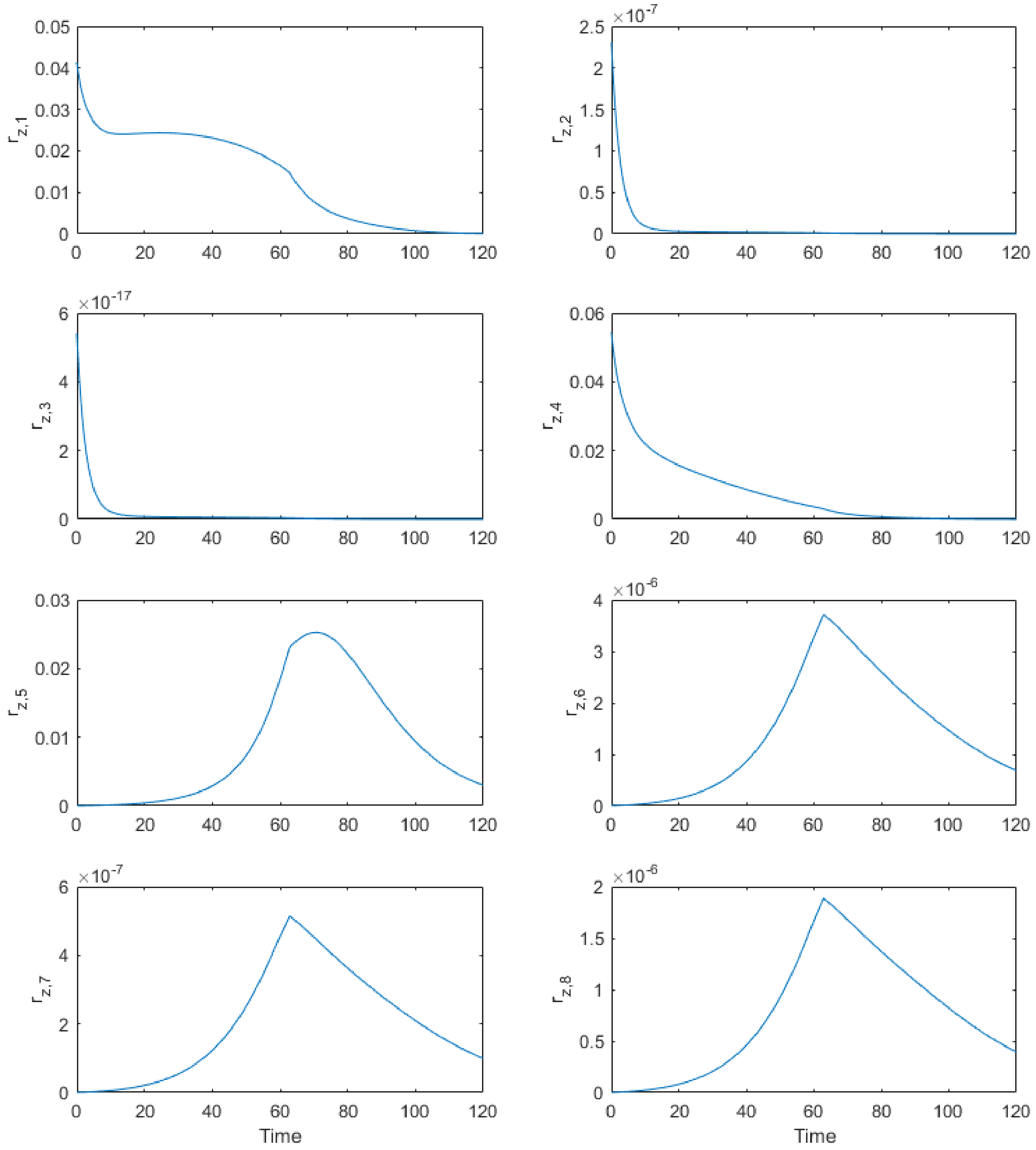

Figure S24. Calculated PSYA-HCM model rates across active EMs in mM/h.

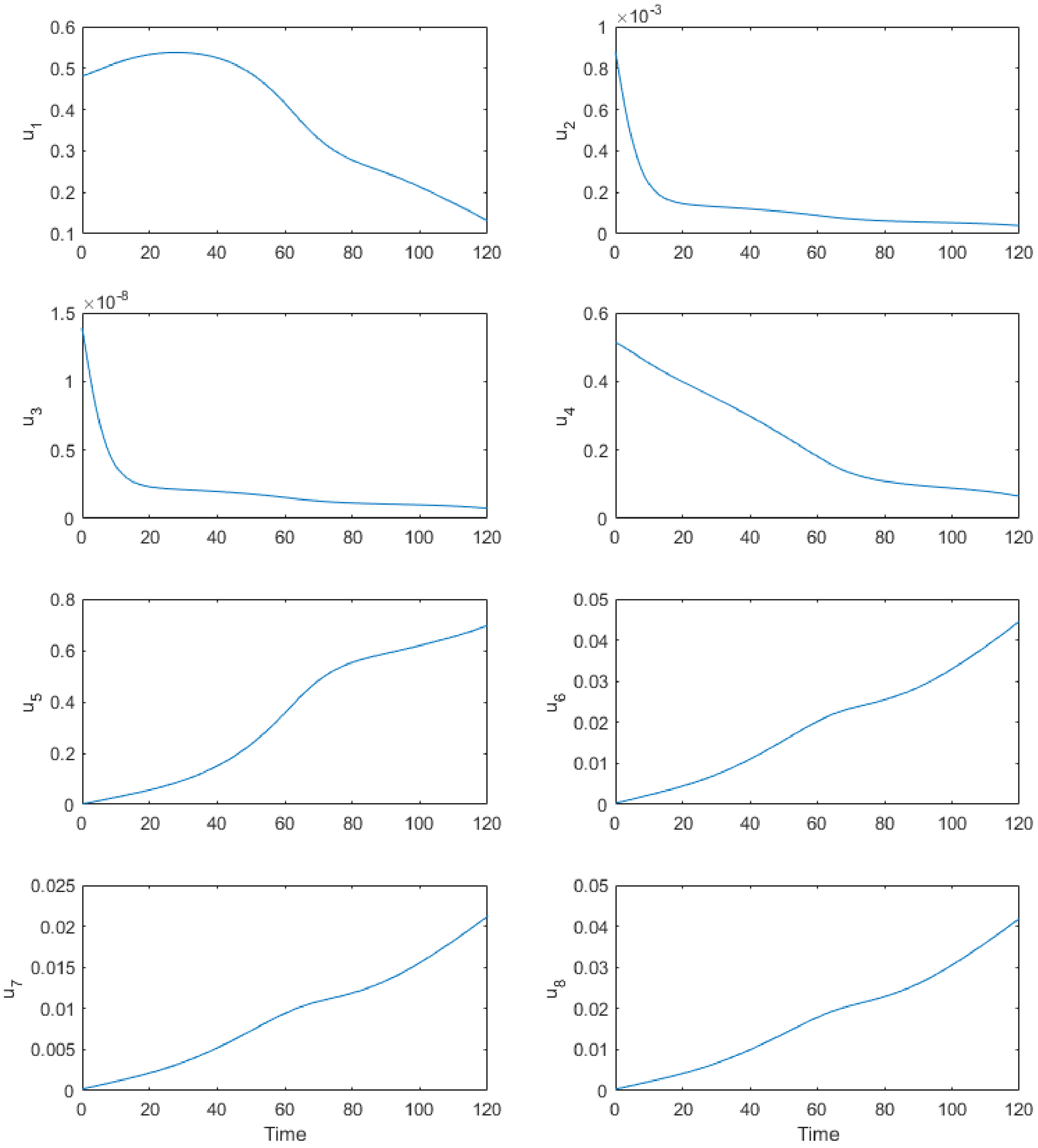

Figure S25. Calculated PSYA-HCM model calculated cybernetic variable u.

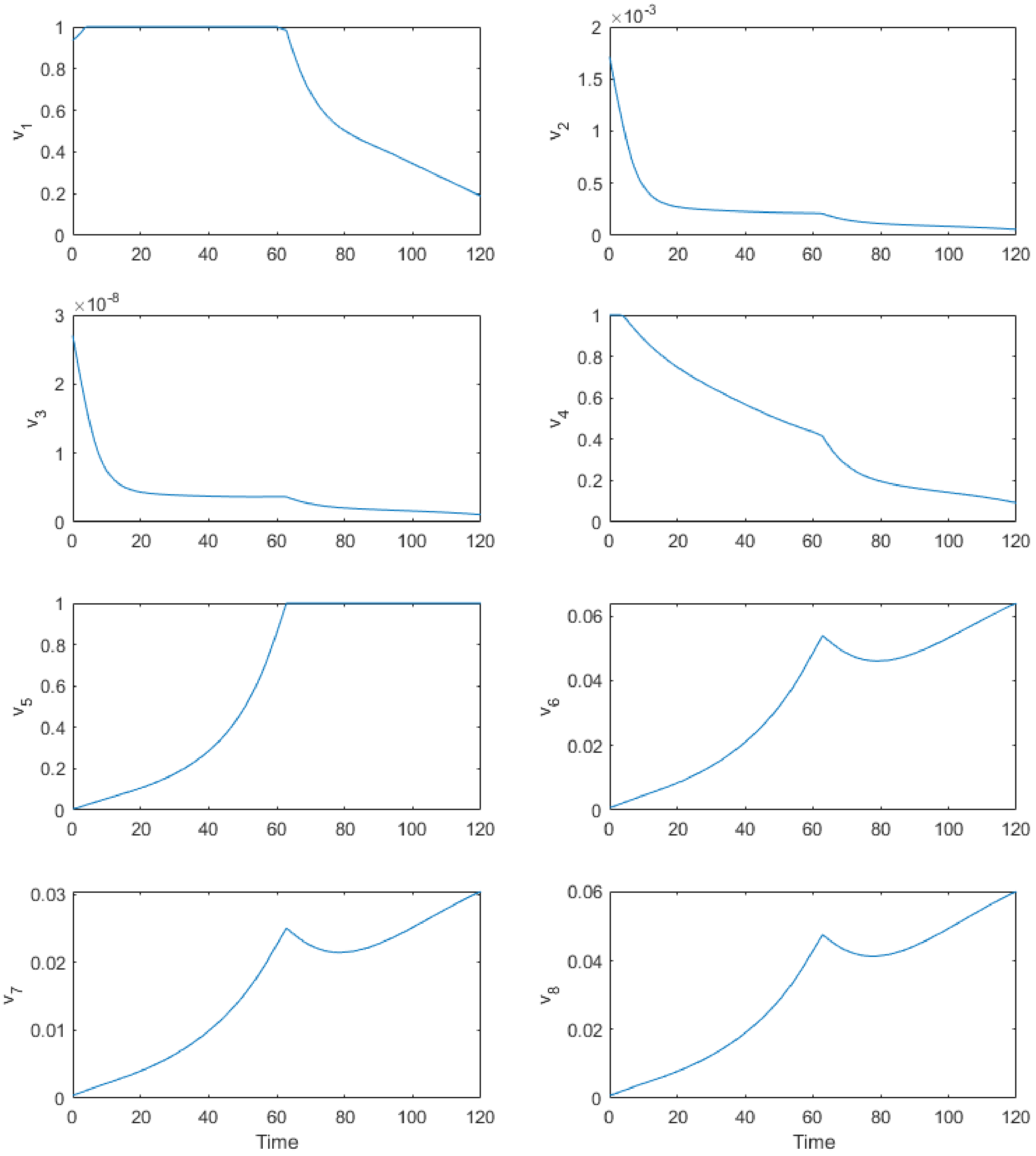

Figure S26. Calculated PSYA-HCM model calculated cybernetic variable v.

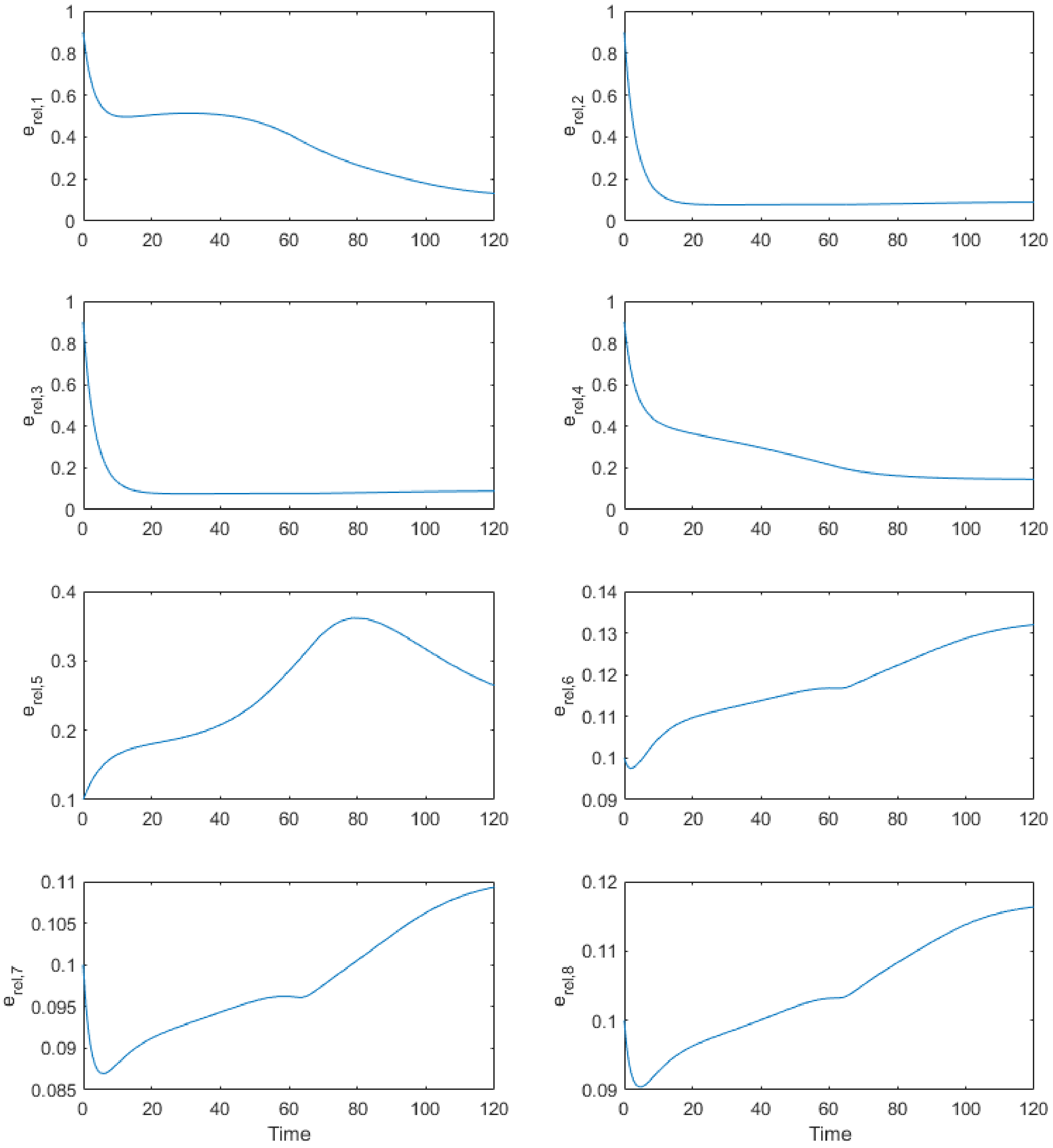

Figure S27. Calculated PSYA-HCM model calculated relative enzyme.

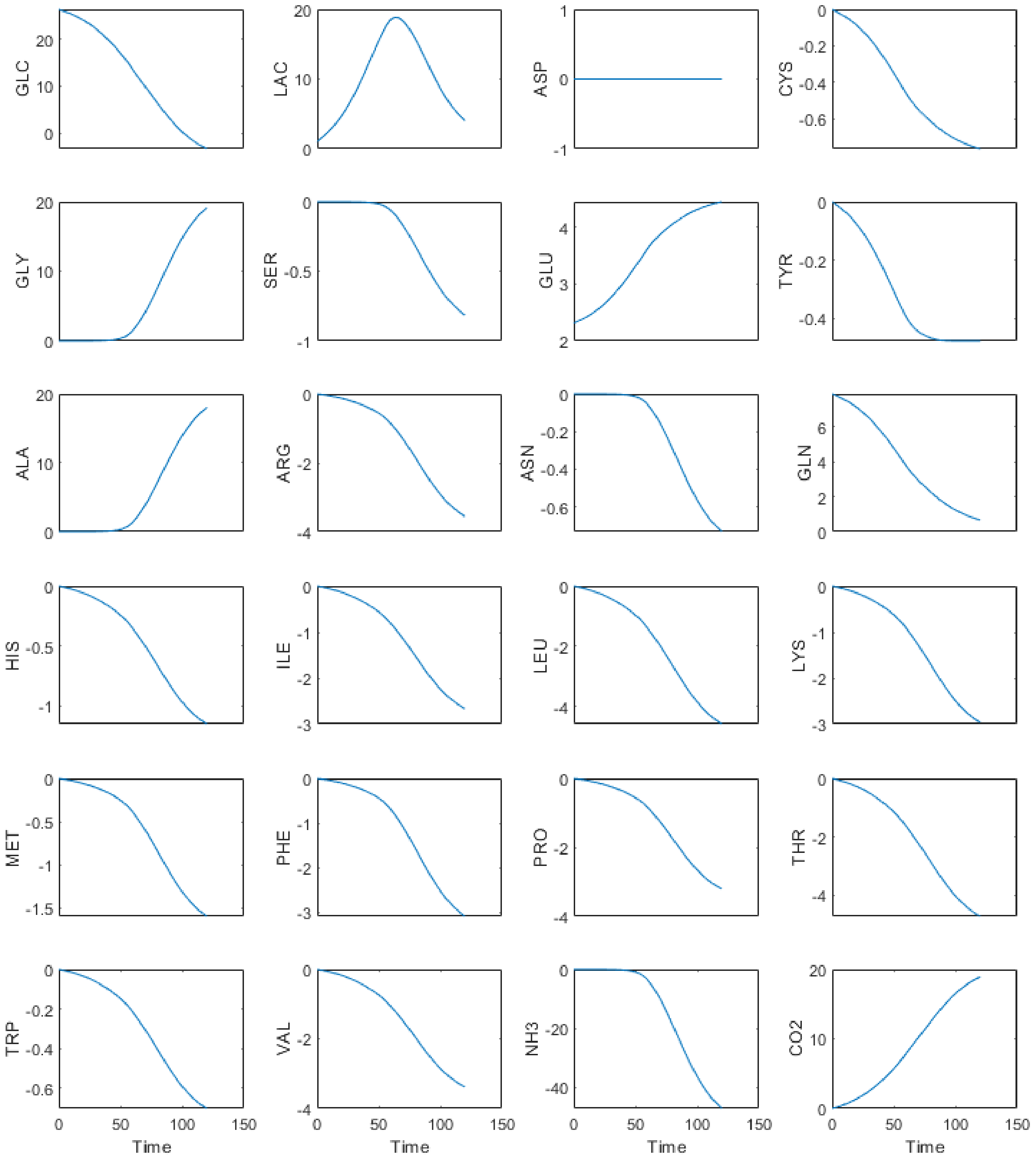

Figure S28. Calculated LPSYA-HCM model extracellular metabolites in mM, non measured metabolites present 0 initial value and therefore are understood as ΔM.

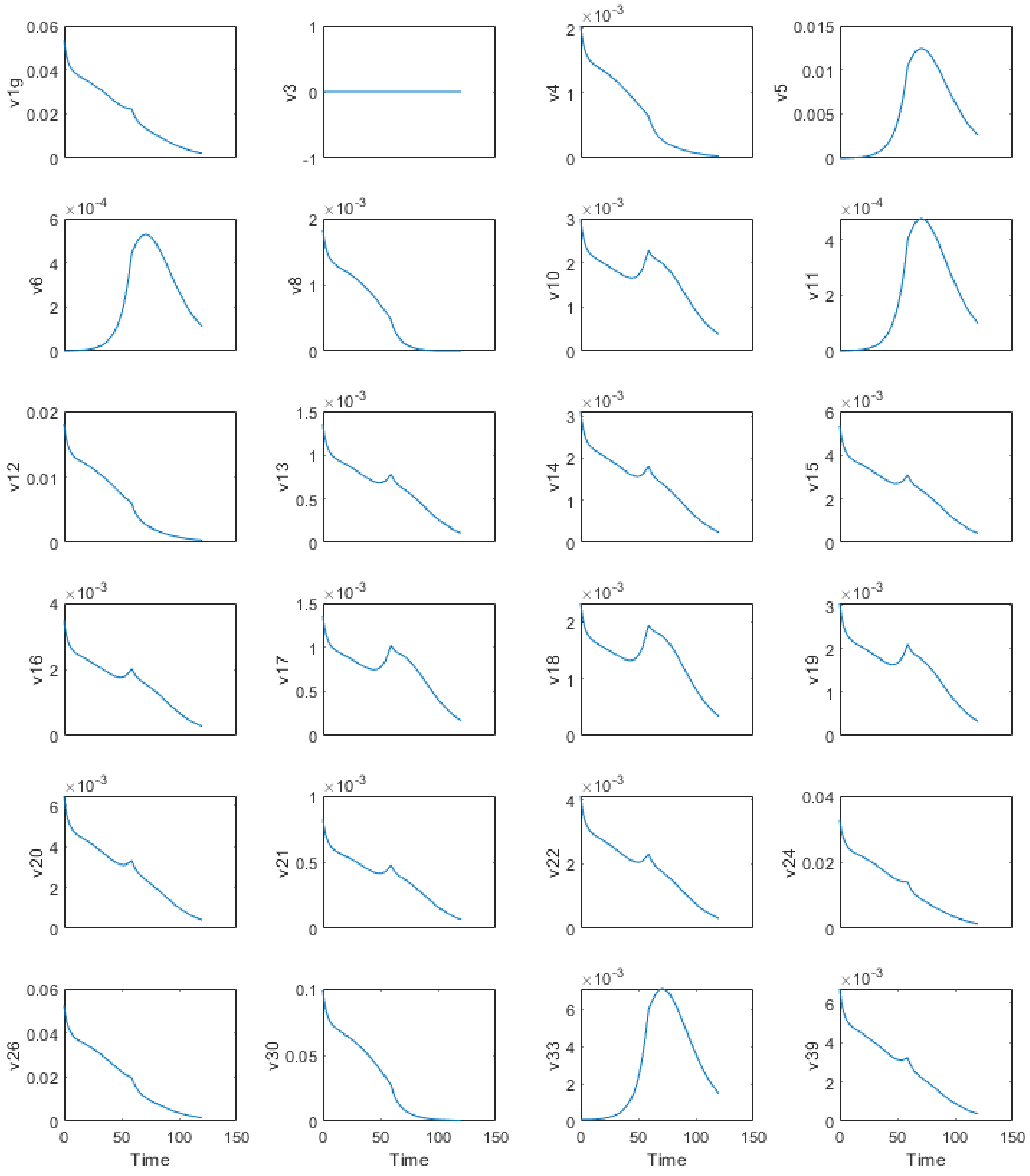

Figure S29. Calculated LPSYA-HCM model fluxes in mM/h pt1.

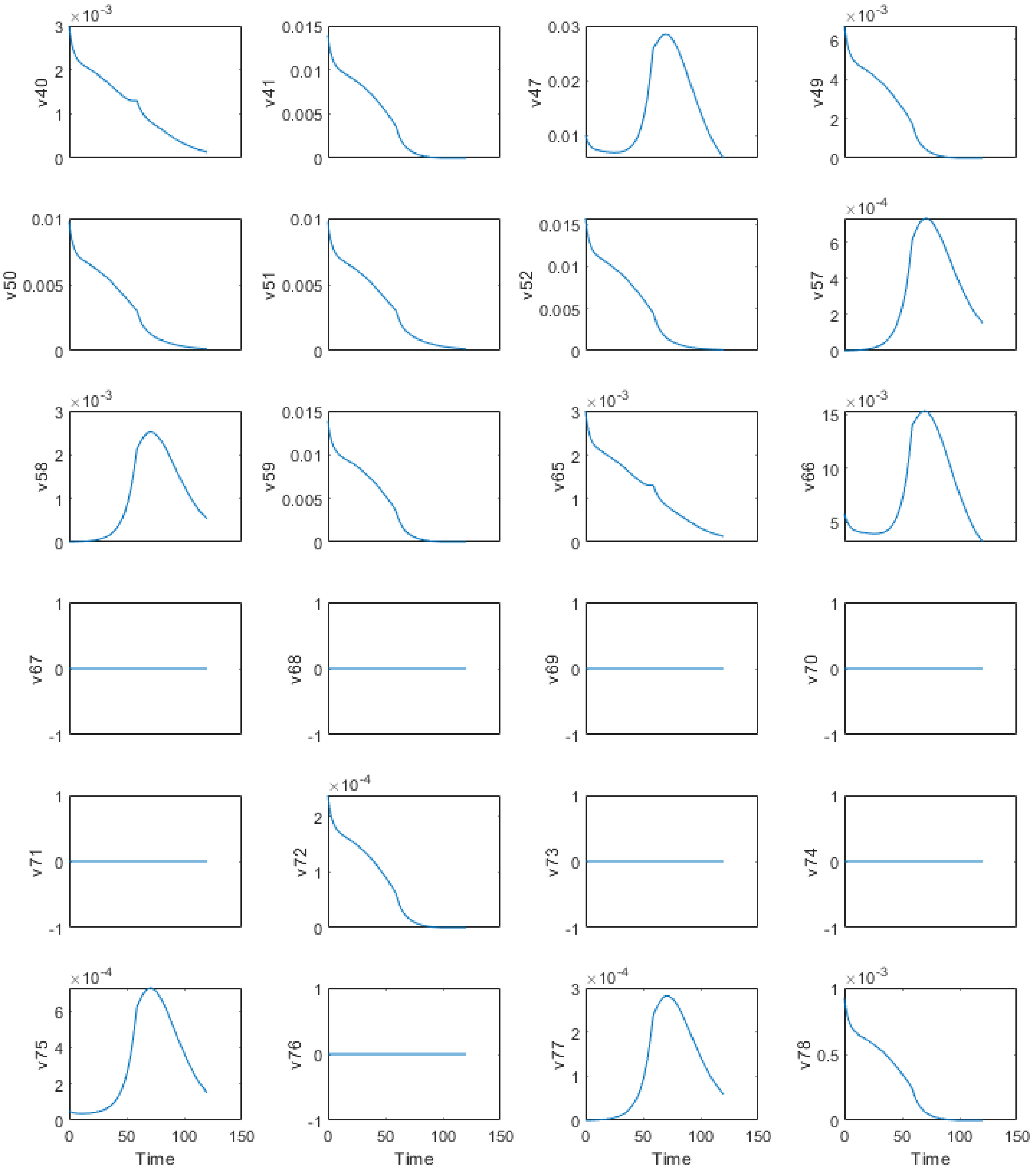

Figure S30. Calculated LPSYA-HCM model fluxes in mM/h pt2.

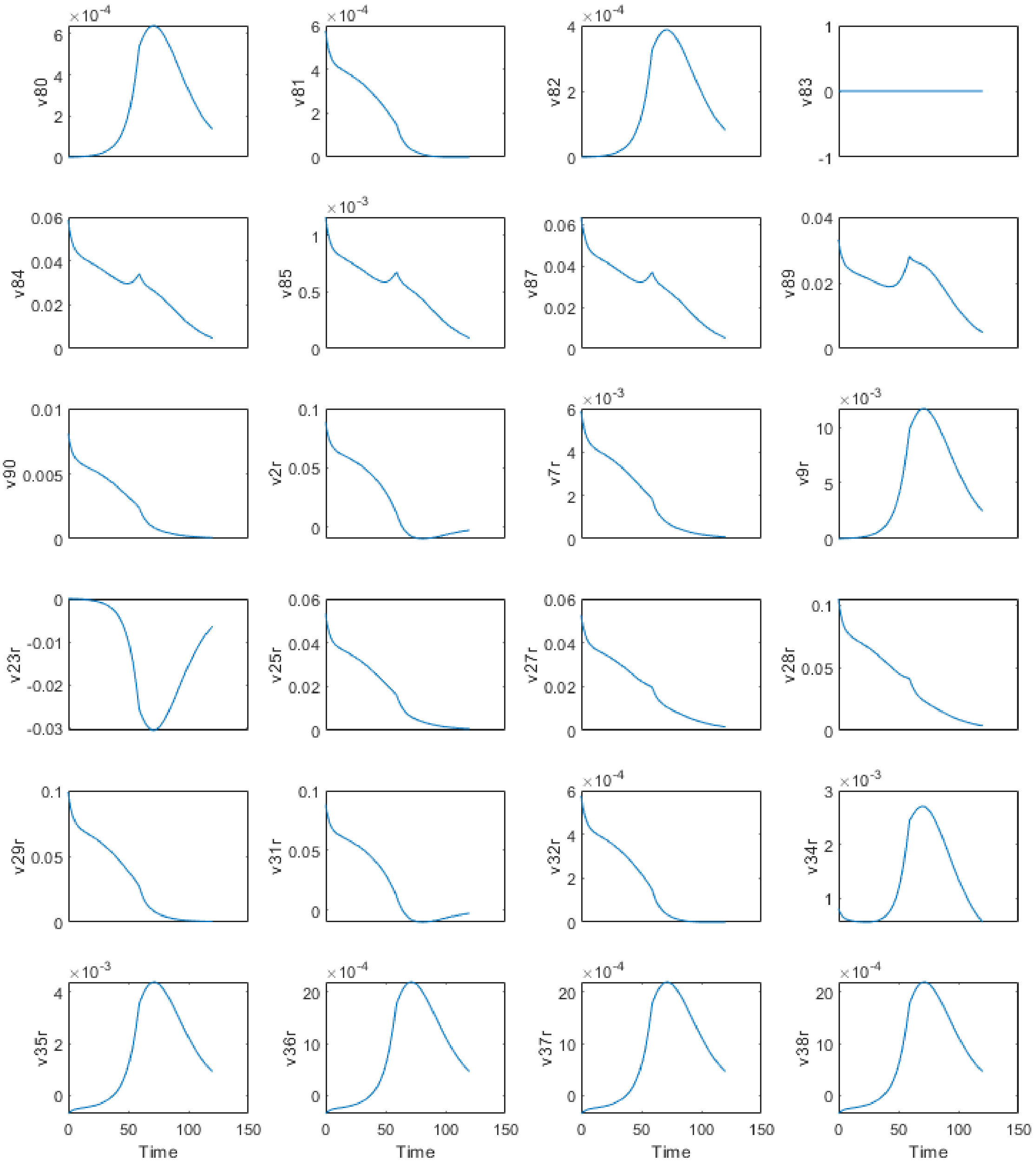

Figure S31. Calculated LPSYA-HCM model fluxes in mM/h pt3.

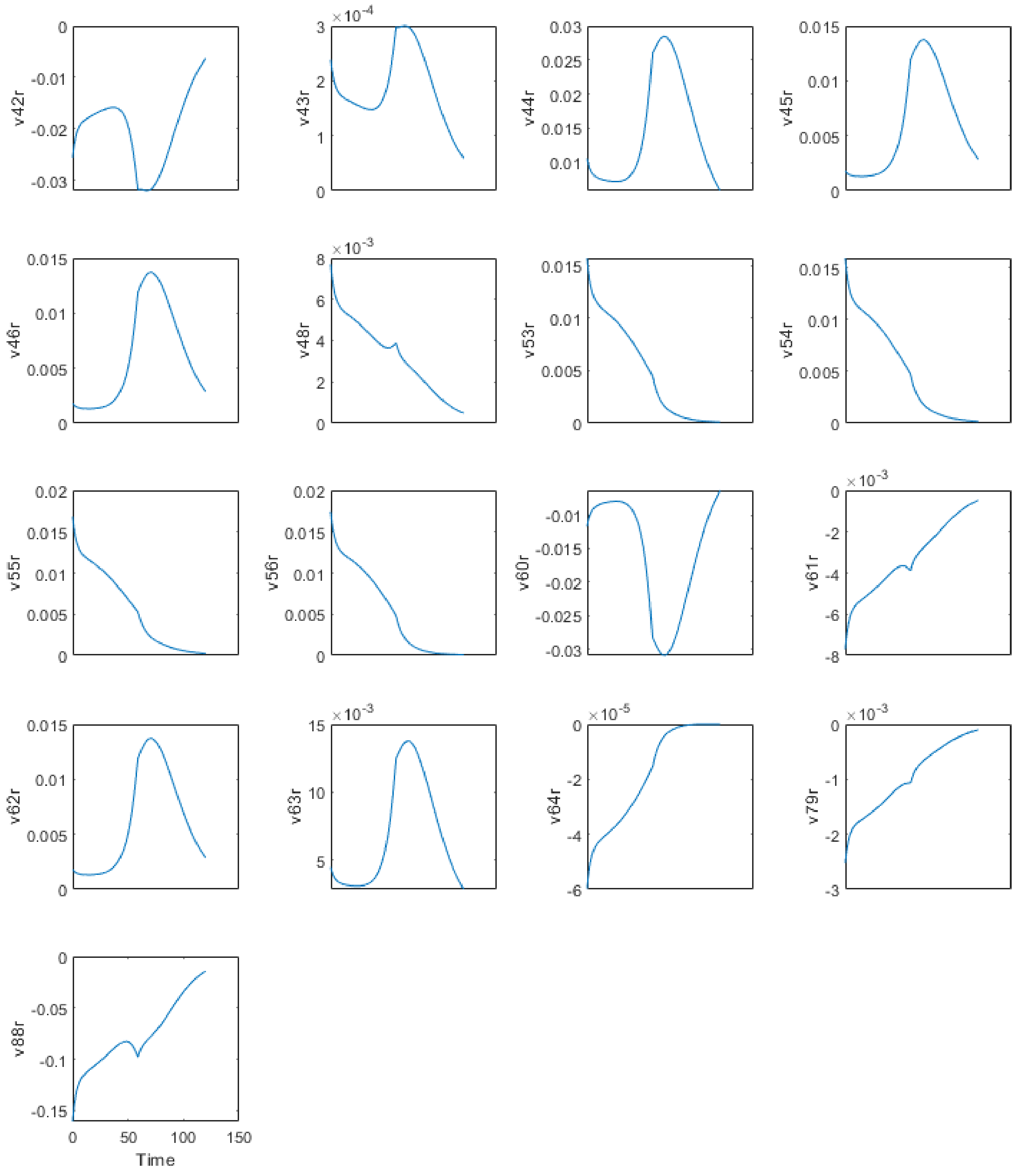

Figure S32. Calculated LPSYA-HCM model fluxes in mM/h pt4.

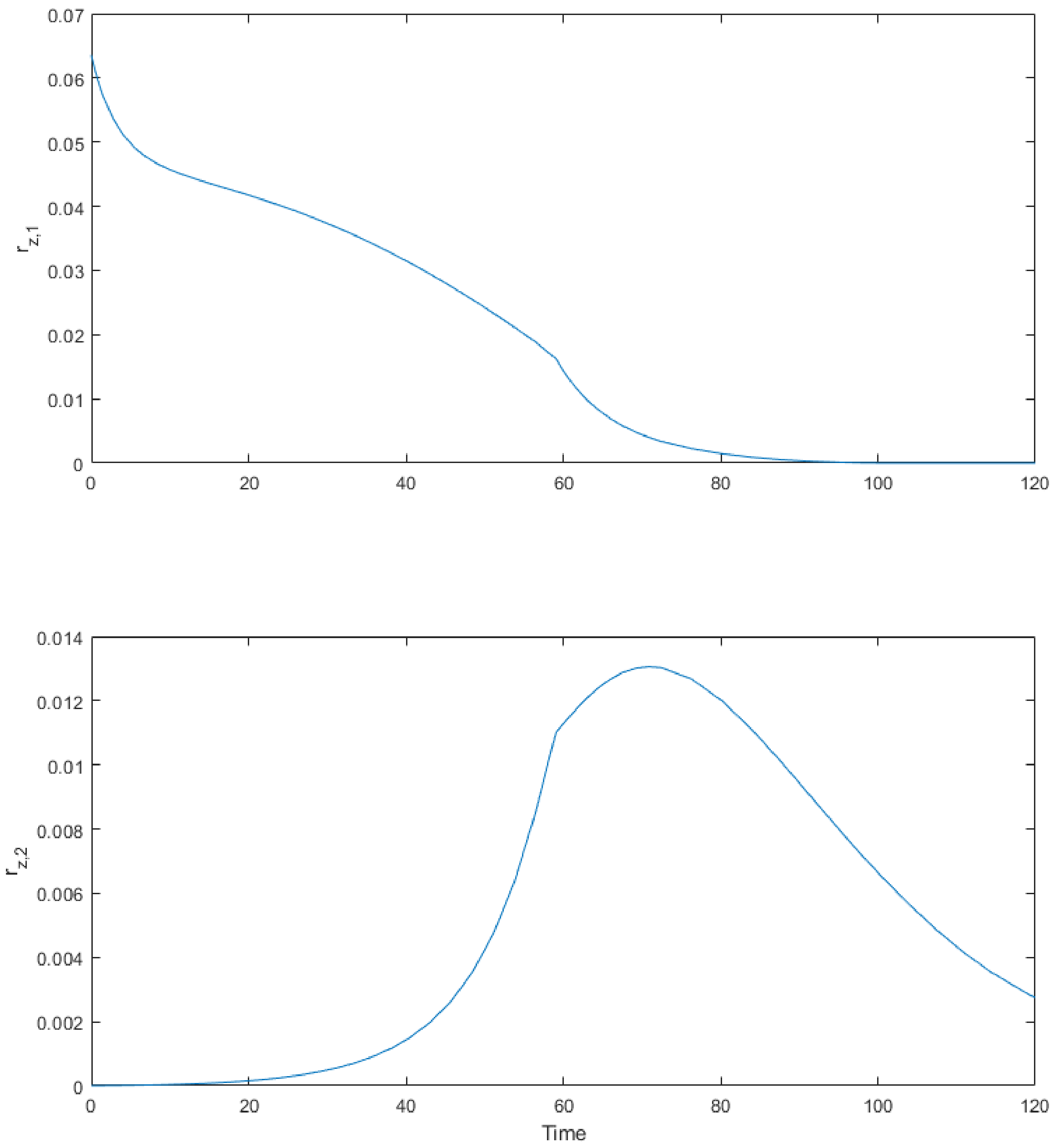

Figure S33. Calculated LPSYA-HCM model rates across active EMs in mM/h.

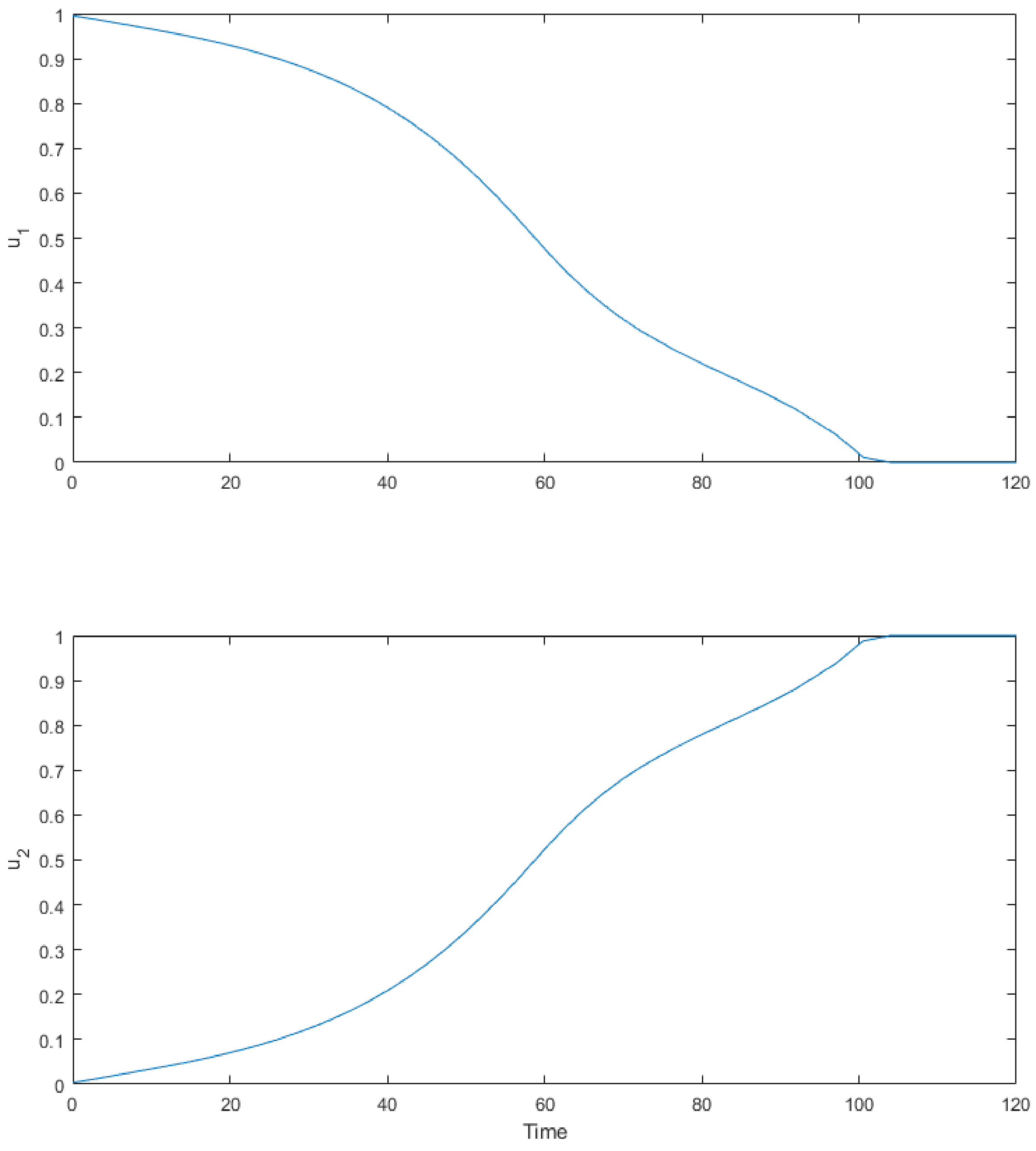

Figure S34. Calculated LPSYA-HCM model calculated cybernetic variable u.

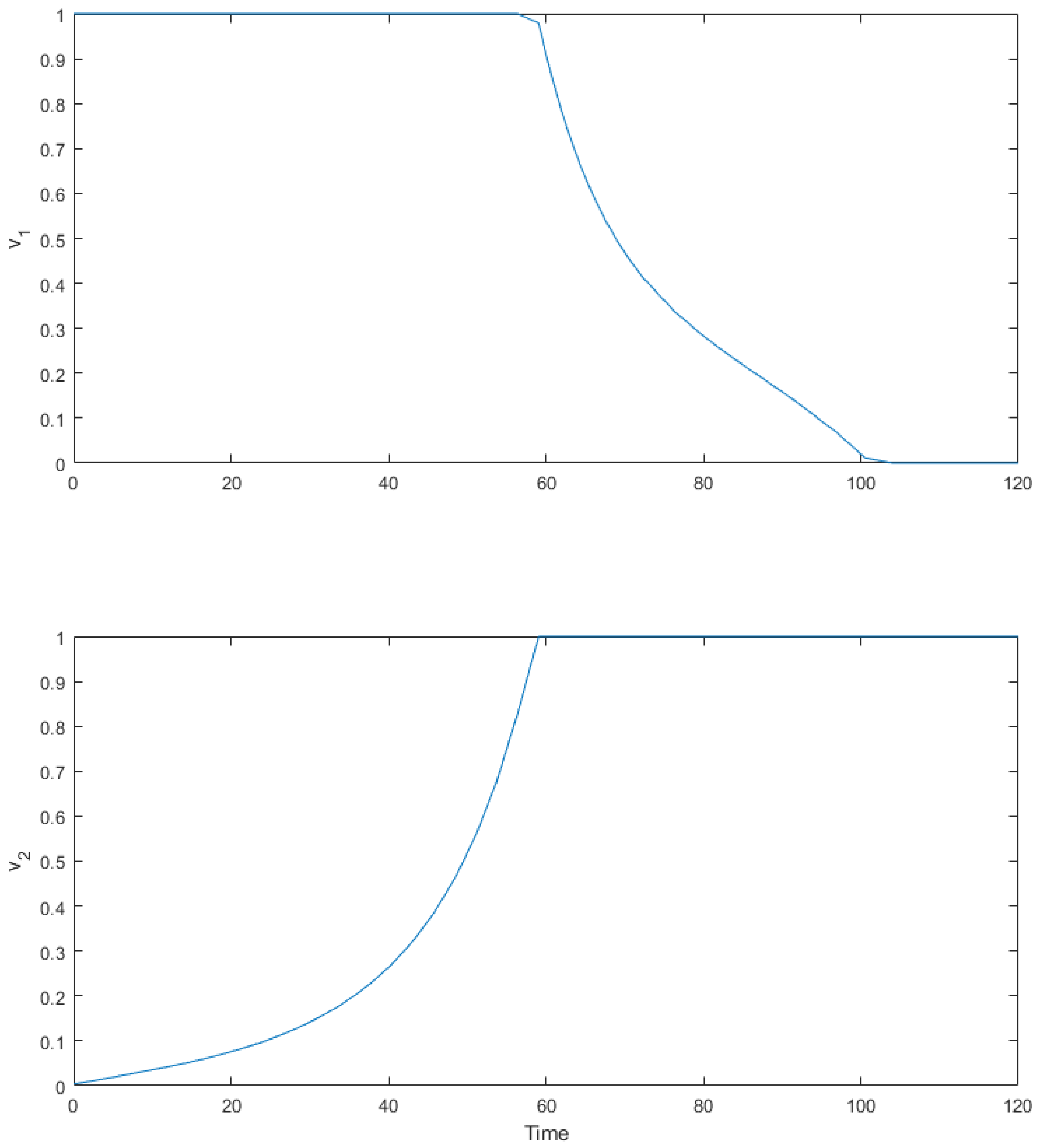

Figure S35. Calculated LPSYA-HCM model calculated cybernetic variable v.

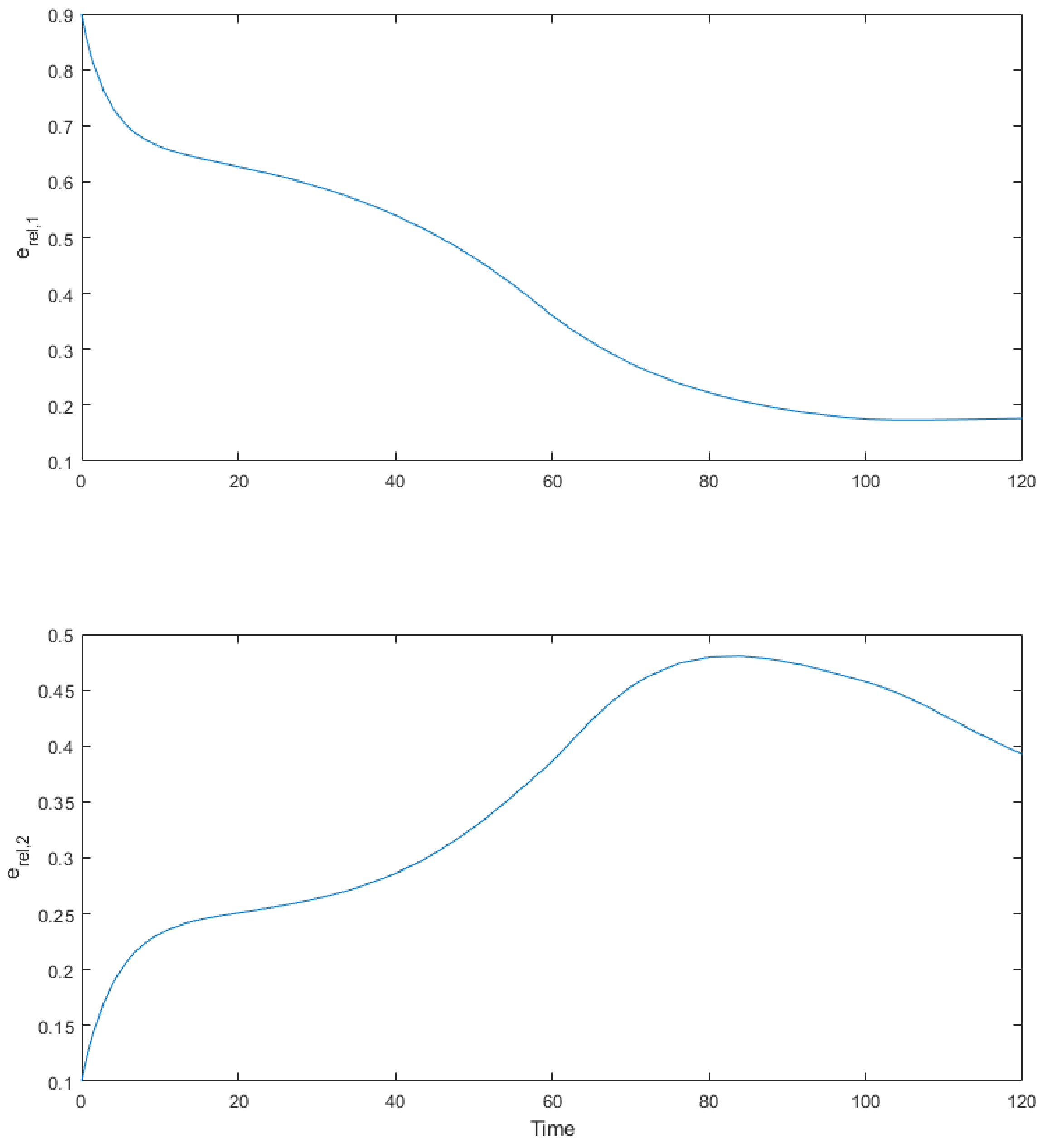

Figure S36. Calculated LPSYA-HCM model calculated relative enzyme.
